# Interactive Session for Residents and Medical Students on Dermatologic Care for Lesbian, Gay, Bisexual, Transgender, and Queer Patients

**DOI:** 10.15766/mep_2374-8265.11148

**Published:** 2021-04-21

**Authors:** Devon L. Barrett, Krittin J. Supapannachart, Ramoncito L. Caleon, Laura Ragmanauskaite, Patrick McCleskey, Howa Yeung

**Affiliations:** 1 Third-Year Medical Student, Department of Dermatology, Emory University School of Medicine; 2 Resident, Department of Dermatology, University of Tennessee Health Science Center; 3 Senior Dermatologist, Kaiser Permanente Oakland Medical Center; 4 Assistant Professor, Department of Dermatology, Emory University School of Medicine; Associate Professor, Regional Telehealth Service, VA Southeast Network VISN 7

**Keywords:** Cultural Competence, Cultural Competency, LGBT Health, LGBTQ+, Sexual and Gender Minorities, Diversity and Inclusion, Dermatology, Online/Distance Learning, Diversity, Inclusion, Health Equity

## Abstract

**Introduction:**

Despite increasing emphasis on LGBTQ health in medical education, evidence-based training on LGBTQ patient care in dermatology is lacking. We designed an interactive online didactic session on dermatologic care of LGBTQ patients for medical students and dermatology residents.

**Methods:**

Session content was based on continuing medical education articles and incorporated preexisting LGBTQ-inclusive policies, environments, and videos. We implemented the session via a web-based videoconferencing platform as part of a preexisting resident lecture series. We began with a 90-minute lecture on LGBTQ health care disparities and dermatologists’ roles, best practices for providing inclusive care, and dermatologic health concerns and screening recommendations in LGBTQ populations. To solidify knowledge and promote practice of learned skills, a 30-minute interactive role-playing session followed where participants acted as observer, patient, or provider in three distinct clinical scenarios pertaining to dermatologic care of LGBTQ patients. Participants completed baseline and follow-up surveys, which included a psychometrically validated clinical skills scale and an ad hoc knowledge assessment.

**Results:**

Baseline and follow-up scores from the clinical skills scale increased overall (0.7; 95% CI, 0.5–0.9; *p* < .001), in self-reported clinical preparedness (1.1; 95% CI, 0.5–1.6; *p* = .001), and in basic knowledge (0.8; 95% CI, 0.3–1.4; *p* = .003).

**Discussion:**

An online interactive didactive session on dermatological care of LGBTQ patients increased participants’ clinical preparedness and basic knowledge. Implementation of similar sessions at other institutions can improve gaps in preparing residents and medical students in dermatological care of LGBTQ patients.

## Educational Objectives

By the end of this educational session, learners will be able to:

1. Identify appropriate terminology and language when caring for LGBTQ patients.
2. Describe common skin concerns in LGBTQ patients and their management.
3. Identify strategies to improve dermatologic care for LGBTQ patients in clinical practices.

## Introduction

LGBTQ persons living in the United States experience discrimination, have limited health care access, and receive lower quality of care.^1,2^ A 2011 Institute of Medicine report highlighted the lack of provider knowledge in caring for the 10 million LGBTQ individuals living in the United States.^3^ Consequently, there is increasing national emphasis on learning how to provide culturally responsive care for LGBTQ patients in undergraduate^4^ and graduate medical education across specialties.^3,5,6^ Doctors renewing their medical licenses are required by law in one jurisdiction to have at least 2 hours of LGBTQ health-related continuing medical education,^5^ and new curricula aimed at training internal medicine practitioners on caring for LGBTQ populations are being developed.^7^

New curricula teaching medical trainees how to care for LGBTQ populations have utilized didactic lectures, case-based interactive sessions, interactive panels, and practice history taking with standardized LGBTQ patients.^7–11^ While these methods have been shown to improve participant knowledge, many of the new curricula teaching medical trainees how to care for LGBTQ populations have been designed for undergraduate medical education,^7^ with none geared specifically toward dermatologists in training. Dermatologists have long been an important provider of health care among LGBTQ populations; during the 1980s AIDS epidemic, dermatologists played key roles in diagnosing and caring for many patients who were men who had sex with men and had skin manifestations of AIDS, as well as establishing the Gay and Lesbian Dermatology Association.^12–14^ Since then, dermatologists have expanded their role in LGBTQ health by significantly contributing to the growing medical literature on LGBTQ health and diseases common to LGBTQ patients.^14–16^ Dermatologic conditions important to the LGBTQ community currently include increased risk of skin cancer and indoor tanning in gay and bisexual men, acne and hair-related changes associated with hormone therapy in transgender persons, and increased risk for oral human papillomavirus (HPV) due to decreased rates of HPV vaccination in individuals who engage in same-sex sexual activity.^17–19^

The Accreditation Council for Graduate Medical Education Competencies recognize the importance of LGBTQ health by requiring dermatology residents to demonstrate responsiveness in caring for LGBTQ populations^6^; however, many dermatology residency programs still lack formal curricula.^20^ Furthermore, no study has examined the efficacy of training in LGBTQ cultural responsiveness in dermatology.^21^ We aimed to examine self-reported clinical preparedness, attitudinal awareness, and basic knowledge of medical students and residents before and after an interactive online didactic session on caring for LGBTQ patients in dermatology.

## Methods

### Setting and Participants

We implemented our work as part of a 2-hour installment of the Emory dermatology resident lecture series, which is attended by dermatology residents, third-year medical students, and other trainees within the department of dermatology. Basic knowledge of sexually transmitted infections and descriptive language specific to dermatology was assumed. This session was presented online on April 9, 2020, via a web-based videoconferencing platform. The session was exempted from review by the Emory University Institutional Review Board. Informed consent was obtained from all survey participants.

### Session Development

The curriculum was developed by faculty of the department of dermatology and medical students; the session was predominantly lecture based, with an interactive portion to solidify knowledge and promote practice of learned skills. Session content was developed based on continuing medical education articles on dermatologic care for LGBTQ populations as applied to local practice.^14,22^

We delivered a 90-minute lecture (Appendix A) discussing (1) an overview of LGBTQ health care disparities and dermatologists’ roles, (2) best practices for providing inclusive care, and (3) dermatologic health concerns and screening recommendations in LGBTQ populations. Aimed to engage participants and promote knowledge retention,^23^ the lecture featured LGBTQ-inclusive policies, environments, and electronic medical record use at local clinical sites, as well as preexisting videos on improving cultural responsiveness that included voices from LGBTQ community members.^24,25^

A 30-minute interactive role-play session, adapted from a prior American Academy of Dermatology meeting educational session,^14,22^ was used to reinforce educational concepts via simulation-based learning.^23^ At the end of the didactic session, instructions were explained to all participants by the lecture facilitator. We assigned participants in groups of three to breakout rooms, where they role-played as observer, patient, or provider in three different scenarios: (1) a 58-year-old male in sexual relationships with multiple male partners with a chief complaint of redness and irritation under his foreskin, (2) a 36-year-old gay male with an anal wart, and (3) a 28-year-old transgender male with suspected pityriasis rosea (Appendices B–D). While encouraged to rotate across roles in each scenario, participants could choose to observe across all three scenarios if they felt uncomfortable participating. We emailed case scenarios needed to carry out the interactive session to all participants during the didactic portion of the session based on attendance. Role-play was facilitated by the lecturer and four trained medical students, all of whom had co-host capabilities on the videoconferencing platform and were responsible for assigning attendees to breakout rooms at the start of the session. The medical students and lecturer had practiced assigning co-host permission and breakout room functionality prior to the final presentation.

### Evaluation

To assess the efficacy of the curriculum, we created anonymous baseline (Appendix E) and follow-up (Appendix F) surveys on Google Forms and distributed them via email to residents, medical students, and associated personnel invited to attend the resident lecture series. Three emails were sent within 1 week of the educational session notifying and reminding invitees and attendees to complete baseline and follow-up surveys, respectively.

The baseline survey included (1) participant demographics; (2) questions from the Lesbian, Gay, Bisexual, and Transgender Development of Clinical Skills Scale (LGBT-DOCSS)^26^; (3) questions regarding comfort levels, attitudes, and practice in LGBT care from a survey utilized in a separate study^27^; and (4) an ad hoc knowledge assessment on LGBTQ health in dermatology. The LGBT-DOCSS is a psychometrically validated 18-item survey of 7-point Likert scales designed to assess self-reported clinical preparedness, attitudinal awareness, and basic knowledge related to caring for LGBT patients.^26^ Items were reversed (if appropriate) and averaged overall and across three domains. LGBT-DOCSS scores ranged from 1 to 7, with higher scores denoting higher knowledge and self-reported clinical preparedness and lower attitudinal prejudice. Comfort levels and behavioral practices when caring for LGBTQ patients were assessed using a 22-question survey-of-frequency scale (1 = *Never,* 2 = *Rarely,* 3 = *Sometimes,* 4 = *Often,* 5 = *Always*) from a separate assessment tool.^27^ A seven-item ad hoc knowledge assessment on LGBTQ health in dermatology was created based on the educational objectives and reflected lecture content, with scores ranging from 0 to 22.^14,22^ Two participant questions without protected identifiers were used to link baseline and follow-up survey results. The follow-up survey after the session contained the LGBT-DOCSS, knowledge assessment, and feedback on the educational session. Feedback on session effectiveness and value was collected to assess session impact and participants’ reaction to the lecture material and was scored on 5-point Likert scales, with additional free-text comments elicited.

### Analysis

Variables were summarized using descriptive statistics and compared using independent-sample *t* tests or Fisher's exact tests, as appropriate. Changes in mean LGBT-DOCSS domain scores and knowledge test scores before and after the session were compared using paired *t* tests. Missing data were excluded. Two medical students (Devon L. Barrett and Krittin J. Supapannachart) independently coded the feedback comments; discrepancies in qualitative coding were resolved by consensus. All analyses were conducted using IBM SPSS Statistics, version 26, with two-sided tests with 95% confidence intervals and *p* < .05 considered significant.

## Results

Forty-three participants (24 residents and 19 medical students) were invited to the session, and 31 attended (72%; 19 residents, 12 medical students). Twenty-nine attendees completed the baseline survey (15 residents [one PGY 1, three PGY 2s, five PGY 3s, and six PGY 4s] and 14 medical students), and 18 completed the follow-up survey (eight residents and 10 medical students). Response rate for the baseline survey was 91% of attendees, while for the follow-up survey, it was 56% of attendees. Fifteen baseline survey respondents (52%) were female, and six (21%) identified as LGBTQ (Table 1). Baseline self-reported practices, LGBT-DOCSS scores, and knowledge assessment scores did not differ between participants who completed the baseline survey only and those who completed both baseline and follow-up surveys (each *p* > .05, data not shown). LGBT-DOCSS mean baseline scores did not differ based on training level or respondents’ gender identity.

**Table 1.**
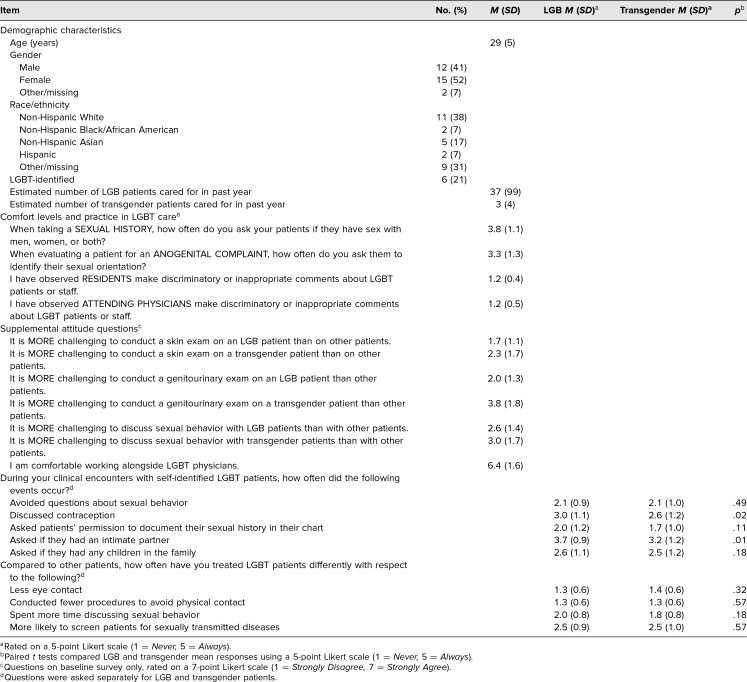
Participants’ Demographic Characteristics, Comfort Levels, and Practice in LGBT Patient Care (*N* = 29)

Our results show that the mean LGBT-DOCSS overall scores and the self-reported clinical preparedness and basic knowledge domain scores increased by 0.7 (95% CI, 0.5–0.9; *p* < .001), 1.1 (95% CI, 0.5–1.6; *p* = .001), and 0.8 (95% CI, 0.3–1.4; *p* = .003) points, respectively, from baseline to follow-up survey (Table 2). Mean number of correct responses on the knowledge assessment increased by 1.6 points (95% CI, 0.02–3.10; *p* < .05; Table 2). Mean baseline, follow-up, and difference scores for each item on the LGBT-DOCSS are shown in Table 3. Six survey questions assessing participants’ attitudes regarding conducting physical examinations of the skin and genitourinary tract of LGBTQ individuals and discussing sexual behavior are not reported.

**Table 2.**
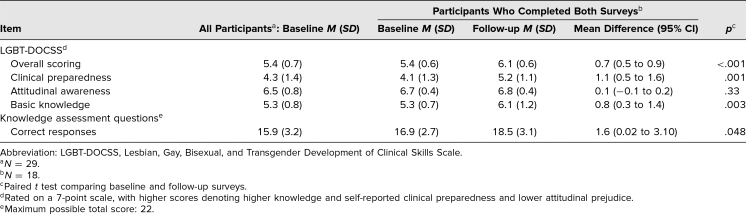
LGBT-DOCSS and Ad Hoc Dermatology Knowledge Assessment on Baseline and Follow-up Surveys

**Table 3.**
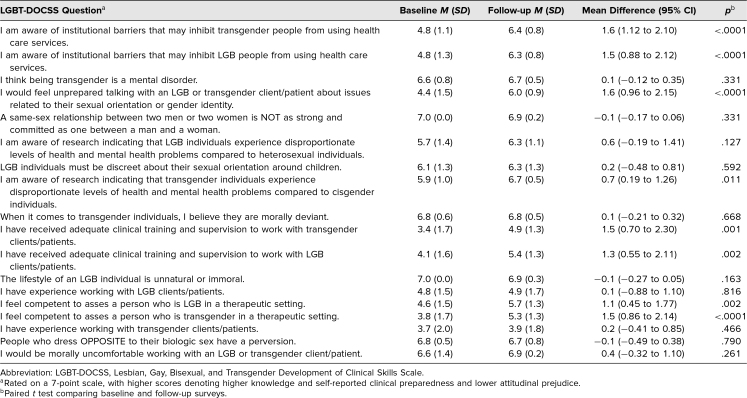
LGBT-DOCCS Question Breakdown

A majority of attendees found the educational content valuable to their practice and found the education materials effective (Table 4); feedback from respondents showed that 14 respondents (78%) noted the lecture was very good or excellent in effectiveness and that 13 (72%) found the session valuable. Participants highlighted five main themes as useful aspects of the didactic session: (1) the overview of how to conduct a comprehensive sexual history, (2) sample questions to use in a sexual history, (3) practicing a sexual history, (4) algorithms for laboratory tests and screenings for patients depending on past medical and sexual history, and (5) information on health disparities in the LGBTQ population (Table 4). Suggestions for improving the lecture included either elongating or shortening the breakout session, clarifying the breakout session instructions, increasing the interactivity of lecture, and condensing lecture information (Table 4). Representative quotes assessing lecture usefulness and lecture suggestions are included in Table 4.

**Table 4.**
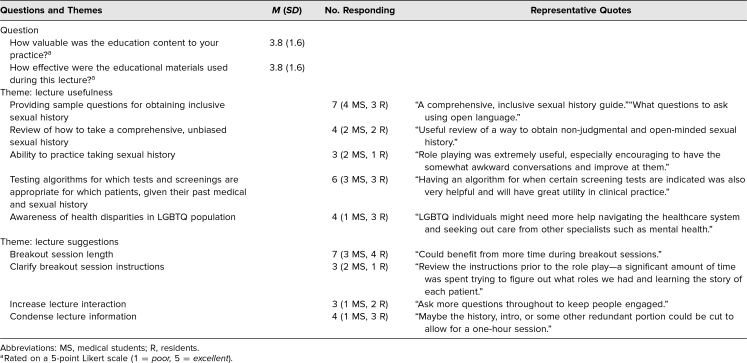
Didactic Session Feedback

## Discussion

A 2-hour online interactive didactic session can add to existing dermatology curricula to improve self-reported clinical preparedness and basic knowledge when caring for members of the LGBTQ community, as measured by the LGBT-DOCSS. Participants’ mean score from a knowledge assessment in dermatology increased, further supporting the efficacy of this training. No change in the attitudinal awareness subscale was noted, which may be due to a ceiling effect from high baseline attitude scores.

Despite new curricula developed to teach providers about LGBT health, training for residents and medical students in the context of dermatology practices is limited.^28^ A survey of 123 residency programs highlighted that 20% had no curricular topics relevant to sexual and gender minorities.^20^ Furthermore, only one of 293 (0.3%) of the American Academy of Dermatology and Society of Pediatric Dermatology's online case modules for medical students mentions LGBT patients.^29^ Insufficient time and lack of faculty expertise were reported as the most common barriers to integrating LGBT health content in dermatology residency curricula.^20^ A short, online, interactive session has the potential to overcome these educational barriers for dermatology trainees across institutions due to its virtual nature, allowing it to be disseminated to a broad audience outside of clinic hours.

While we ultimately recognize the utility of having this session presented in online format, the original concept was to create an educational session with both didactic and interactive components for in-person presentation. However, given the social distancing requirements during the COVID-19 pandemic, the educational session was designed for presentation online. This ultimately led to less administrative hassle, including eliminating the need for reserving a presentation space and audiovisual requirements, and enabled the presentation to be appropriate for distance learning. Training in videoconferencing functionality, including assigning co-host capabilities and practicing assigning individuals to breakout rooms, was paramount to a successful presentation.

Limitations include small sample size in this single-center workshop and lack of psychometric validation of the dermatology knowledge assessment. Terminologies in existing surveys may not be inclusive of all sexual and gender minority persons, such as nonbinary and gender-nonconforming people. Attitudinal awareness scores may have been inflated due to social desirability bias.^30^ The presentation is limited by lack of photographs; future iterations should include photographs added at the discretion of the presenter. Importantly, future presenters should take care to include all skin tones without including stereotyping or stigmatizing images. The presentation does not include a role-play case with a woman who has sex with women; we suggest subsequent iterations include such a case. In-person role-play sessions and iterative refinement of role-play material might improve participant engagement. Observation of clinical responsiveness in caring for standardized or real-life LGBTQ patients in future research could further assess participants’ knowledge retention, application, and associated behavioral changes in practice. Despite these limitations, initial assessment of participants’ receptiveness to and satisfaction with educational materials in the session was needed prior to development of a more robust LGBTQ curriculum. Adapting this didactic session for practicing clinicians and assessing the outcome could expand the generalizability of the results. Future implementation of the session among a larger group of participants could be utilized to confirm assessment score improvement.

After a 2-hour, online, interactive, didactic session on caring for LGBTQ patients in dermatologic settings, participants self-reported higher clinical preparedness and knowledge as measured by a validated survey. This curriculum has the potential to improve education gaps in dermatological care and overcome existing barriers to training.

## Appendices

LGBTQ Curriculum Presentation.pptxCase 1.docxCase 2.docxCase 3.docxBaseline Survey.docxFollow-up Survey.docx
All appendices are peer reviewed as integral parts of the Original Publication.
