# Supplementary material for: Interactive Session for Residents and Medical Students on Dermatologic Care for Lesbian, Gay, Bisexual, Transgender, and Queer Patients
Source: MedEdPORTAL. 2021 Apr 21;17:11148. doi: 10.15766/mep_2374-8265.11148 (PMC8063631; doi:10.15766/mep_2374-8265.11148)
Supplement: Supplementary file 1 — LGBTQ Curriculum Presentation.pptxCase 1.docxCase 2.docxCase 3.docxBaseline Survey.docxFollow-up Survey.docx [file mep_2374-8265.11148-s001.zip › A. LGBTQ Curriculum Presentation.pptx]

## Slide 1
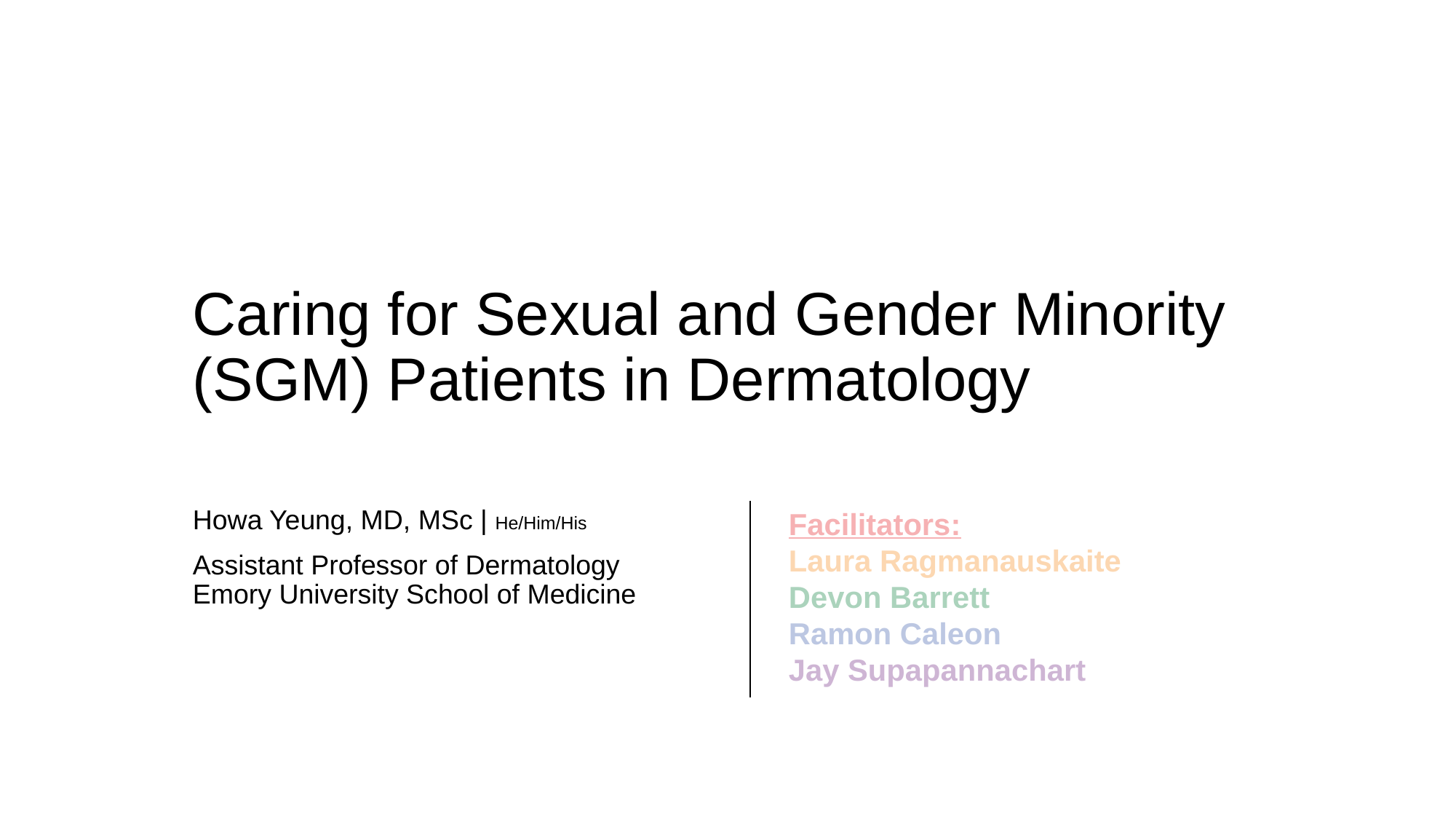

# Caring for Sexual and Gender Minority (SGM) Patients in Dermatology
Facilitators:
Laura Ragmanauskaite
Devon Barrett
Ramon Caleon
Jay Supapannachart
Howa Yeung, MD, MSc | He/Him/His
Assistant Professor of Dermatology
Emory University School of Medicine

## Slide 2
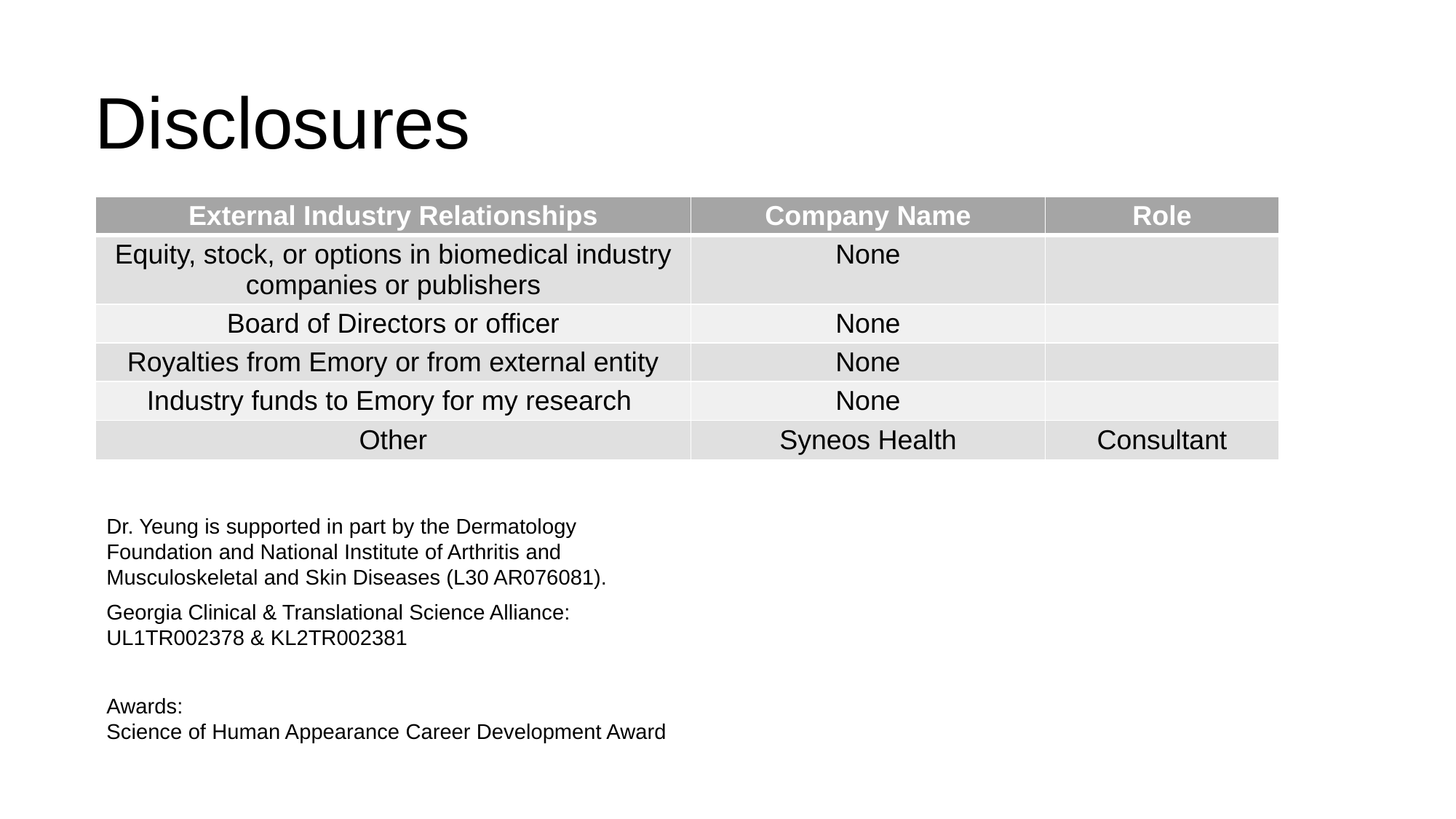

# Disclosures
| External Industry Relationships | Company Name | Role |
| --- | --- | --- |
| Equity, stock, or options in biomedical industry companies or publishers | None | |
| Board of Directors or officer | None | |
| Royalties from Emory or from external entity | None | |
| Industry funds to Emory for my research | None | |
| Other | Syneos Health | Consultant |
Dr. Yeung is supported in part by the Dermatology Foundation and National Institute of Arthritis and Musculoskeletal and Skin Diseases (L30 AR076081).
Georgia Clinical & Translational Science Alliance: UL1TR002378 & KL2TR002381
Awards:
Science of Human Appearance Career Development Award

## Slide 3
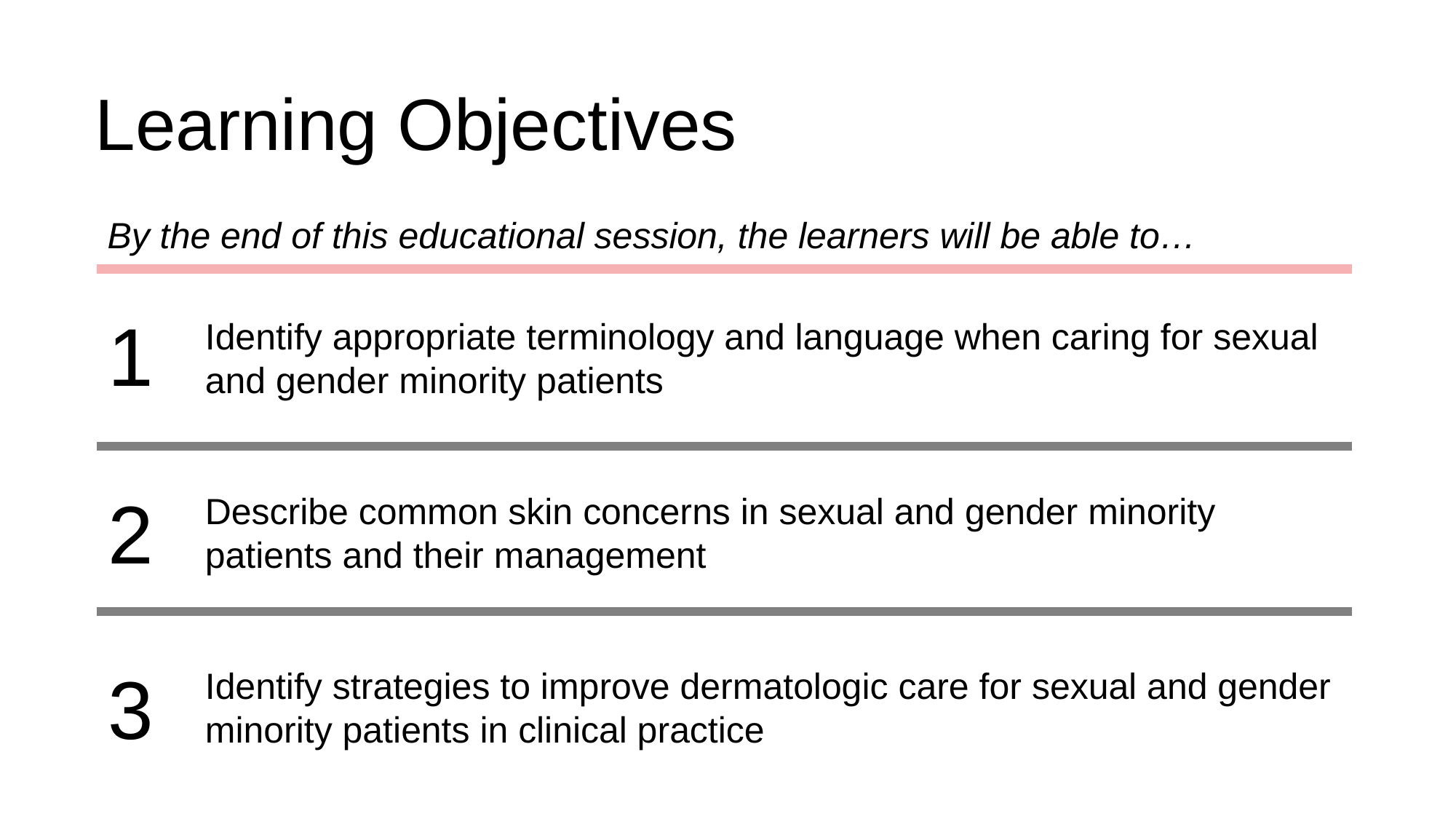

# Learning Objectives
By the end of this educational session, the learners will be able to…
1
Identify appropriate terminology and language when caring for sexual and gender minority patients
Describe common skin concerns in sexual and gender minority patients and their management
Identify strategies to improve dermatologic care for sexual and gender minority patients in clinical practice
2
3

## Slide 4
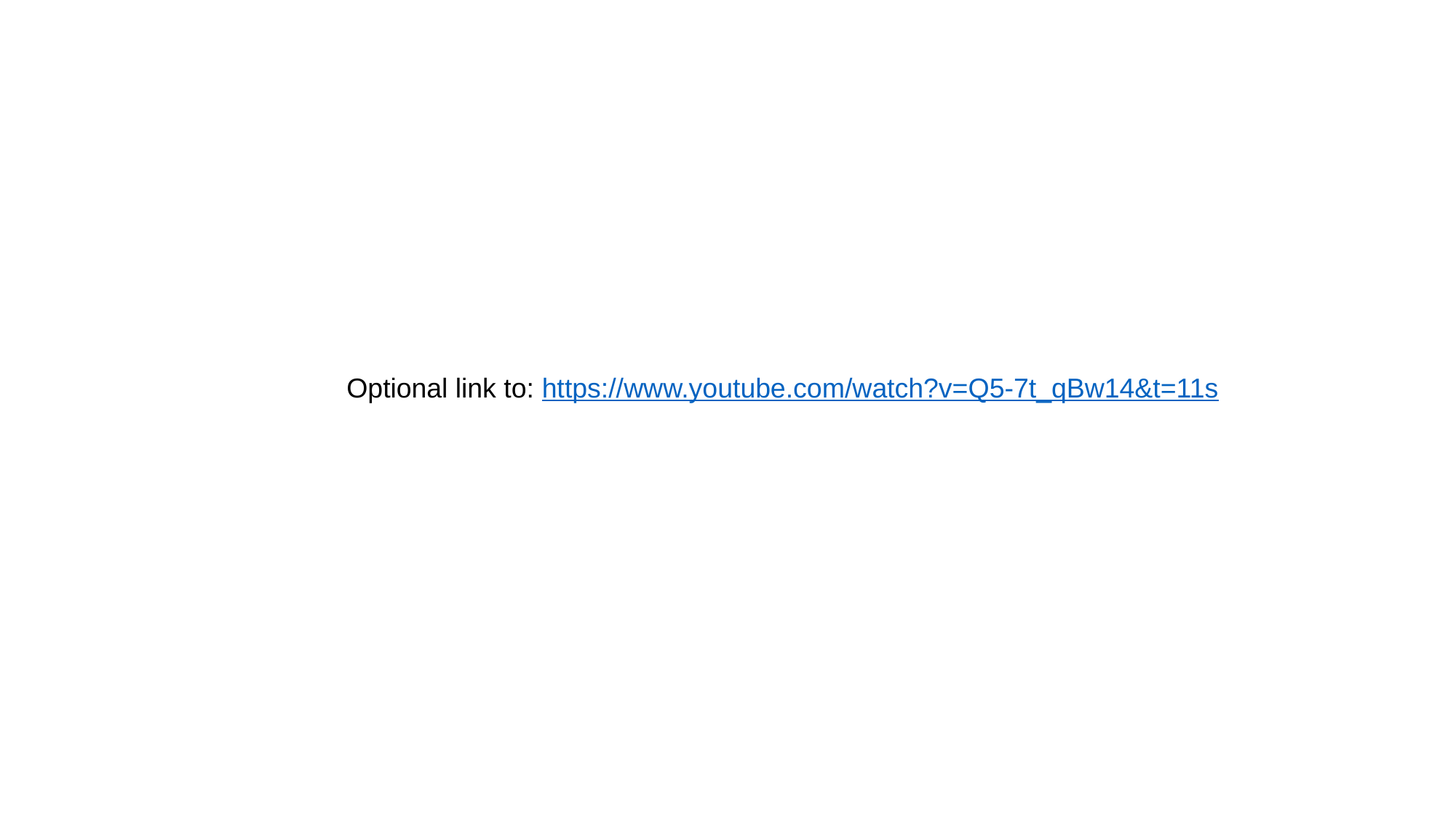

Optional link to: https://www.youtube.com/watch?v=Q5-7t_qBw14&t=11s

## Slide 5
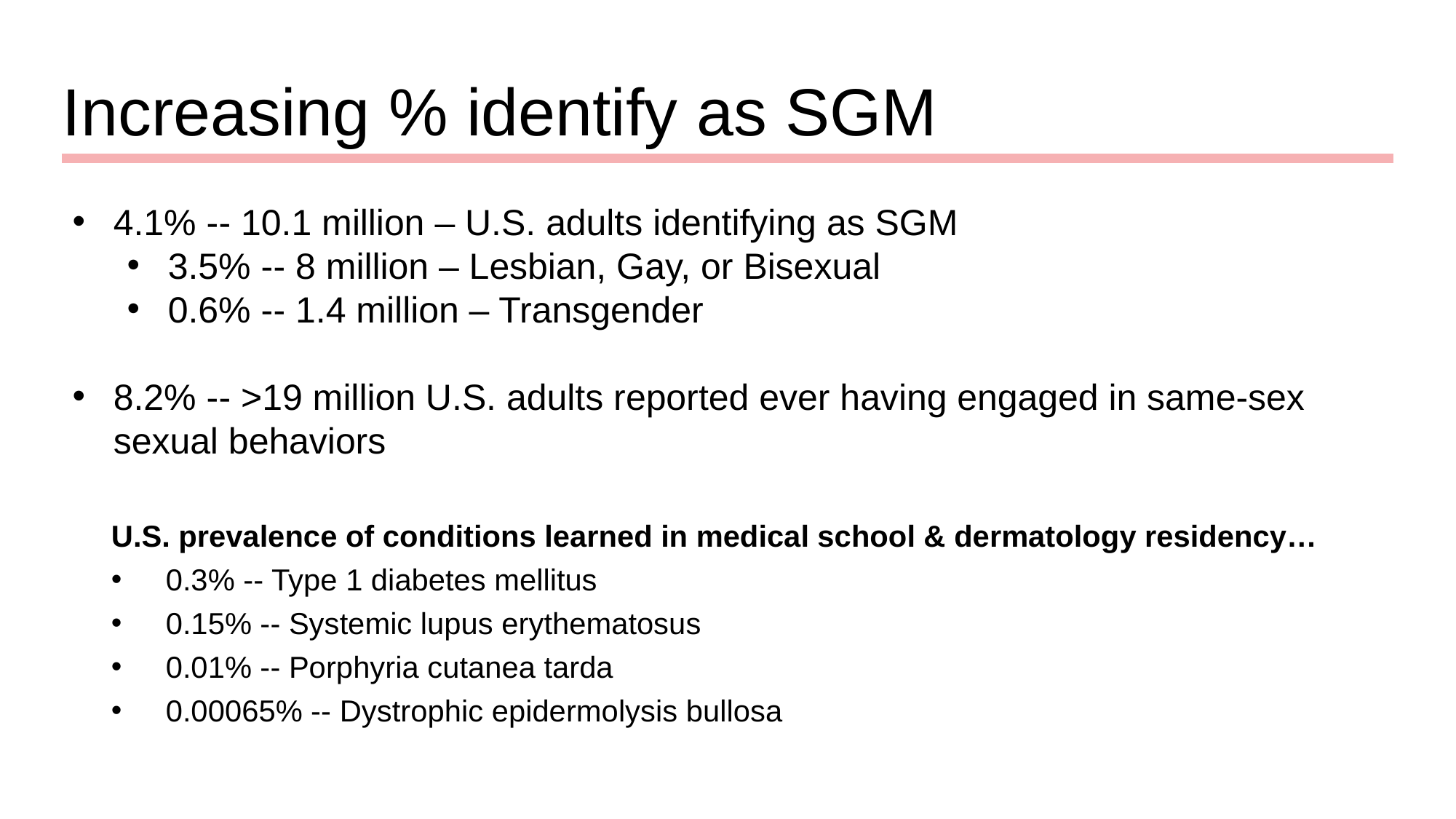

Increasing % identify as SGM
4.1% -- 10.1 million – U.S. adults identifying as SGM
3.5% -- 8 million – Lesbian, Gay, or Bisexual
0.6% -- 1.4 million – Transgender
8.2% -- >19 million U.S. adults reported ever having engaged in same-sex sexual behaviors
U.S. prevalence of conditions learned in medical school & dermatology residency…
0.3% -- Type 1 diabetes mellitus
0.15% -- Systemic lupus erythematosus
0.01% -- Porphyria cutanea tarda
0.00065% -- Dystrophic epidermolysis bullosa

## Slide 6
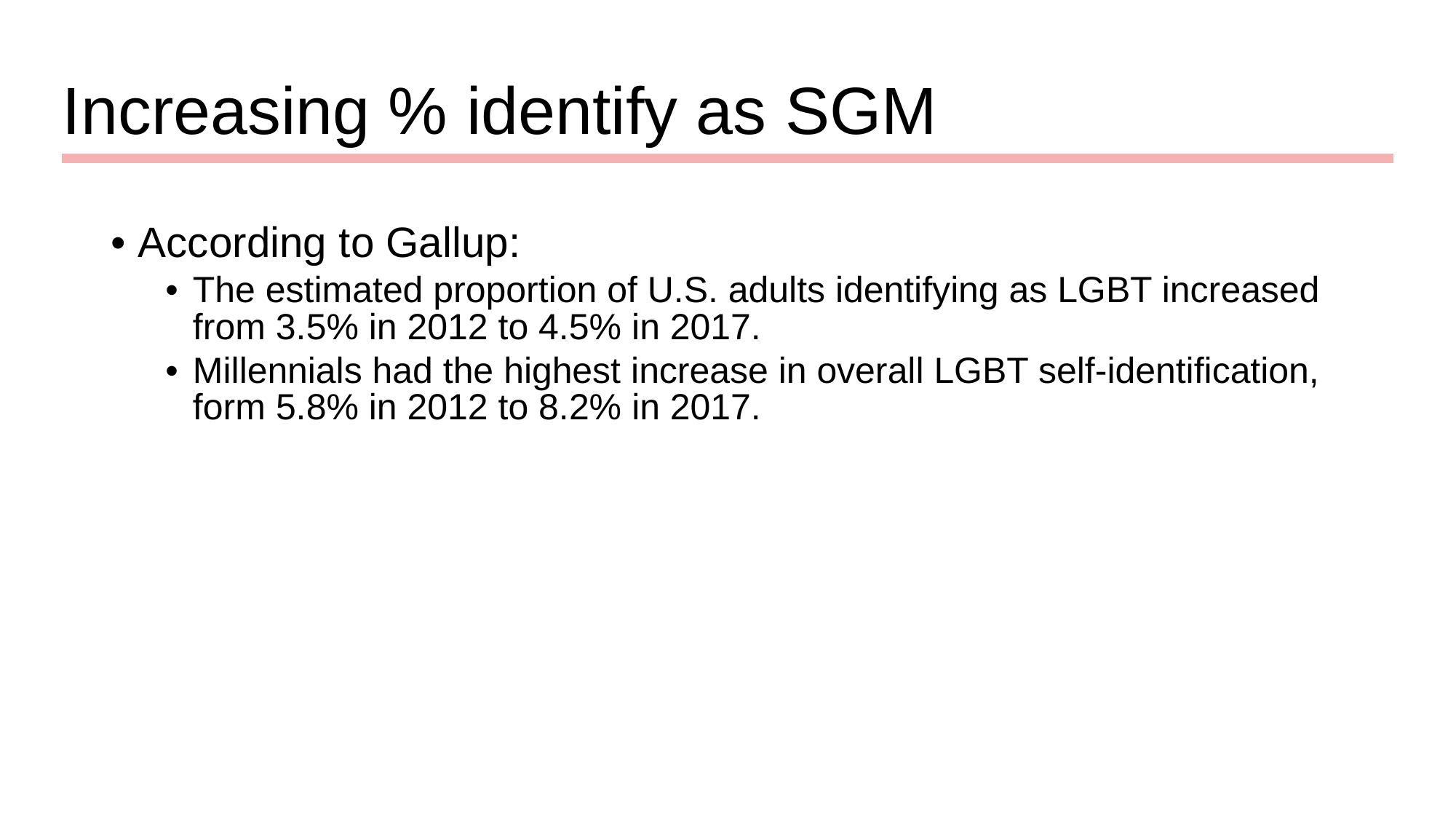

Increasing % identify as SGM
According to Gallup:
The estimated proportion of U.S. adults identifying as LGBT increased from 3.5% in 2012 to 4.5% in 2017.
Millennials had the highest increase in overall LGBT self-identification, form 5.8% in 2012 to 8.2% in 2017.

## Slide 7
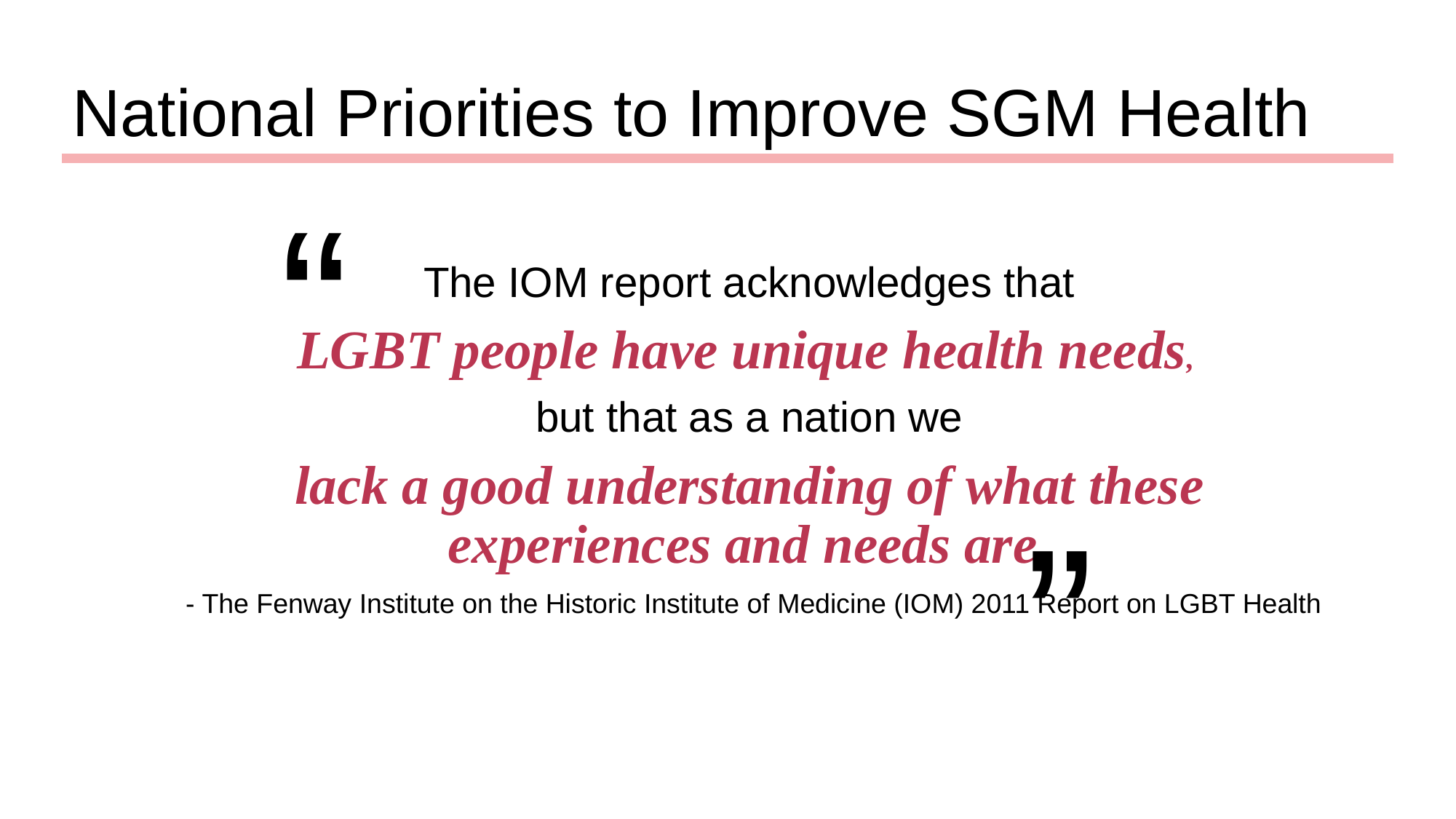

National Priorities to Improve SGM Health
“
The IOM report acknowledges that
LGBT people have unique health needs,
but that as a nation we
lack a good understanding of what these experiences and needs are.
- The Fenway Institute on the Historic Institute of Medicine (IOM) 2011 Report on LGBT Health
”

## Slide 8
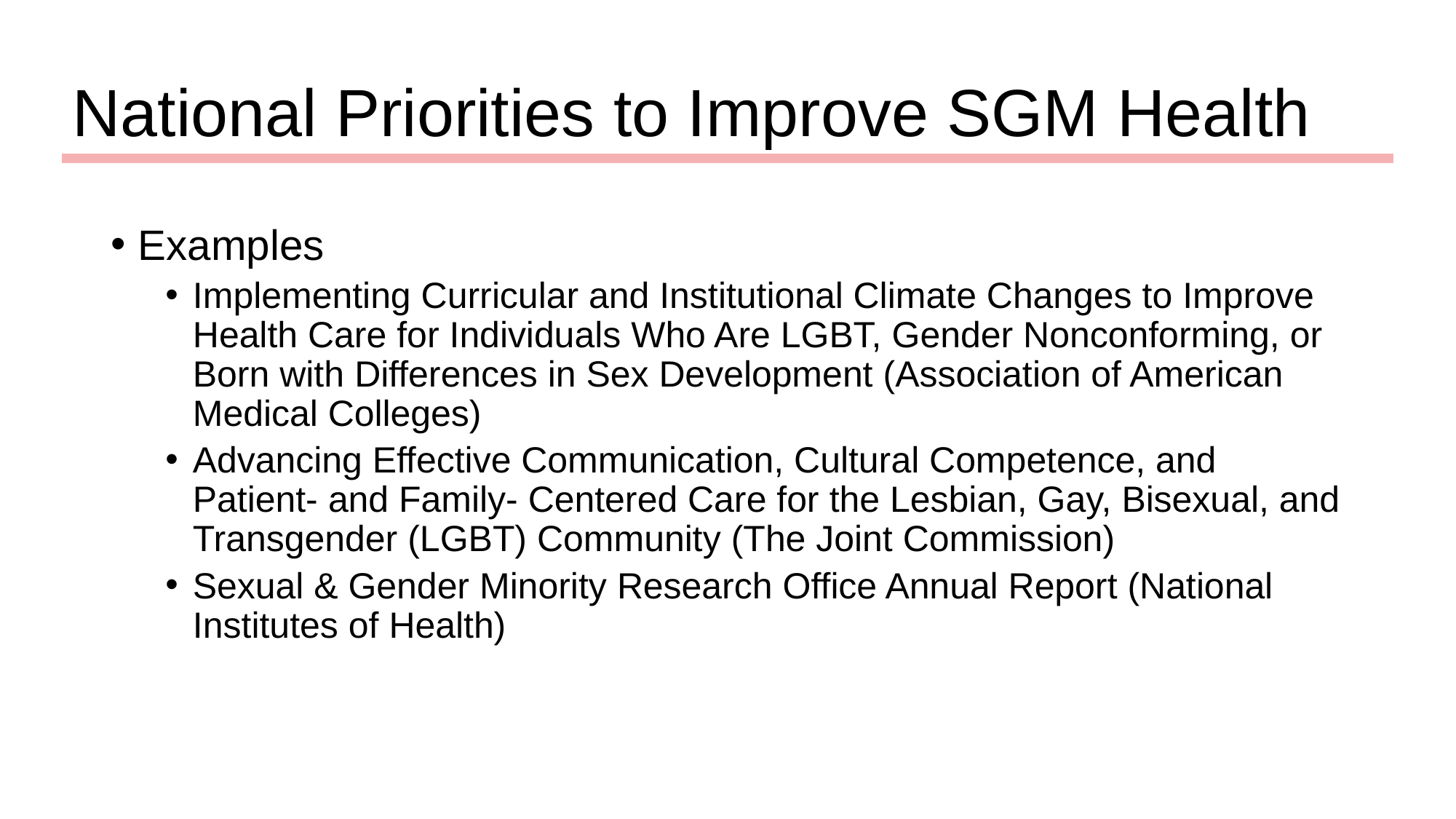

National Priorities to Improve SGM Health
Examples
Implementing Curricular and Institutional Climate Changes to Improve Health Care for Individuals Who Are LGBT, Gender Nonconforming, or Born with Differences in Sex Development (Association of American Medical Colleges)
Advancing Effective Communication, Cultural Competence, and Patient- and Family- Centered Care for the Lesbian, Gay, Bisexual, and Transgender (LGBT) Community (The Joint Commission)
Sexual & Gender Minority Research Office Annual Report (National Institutes of Health)

## Slide 9
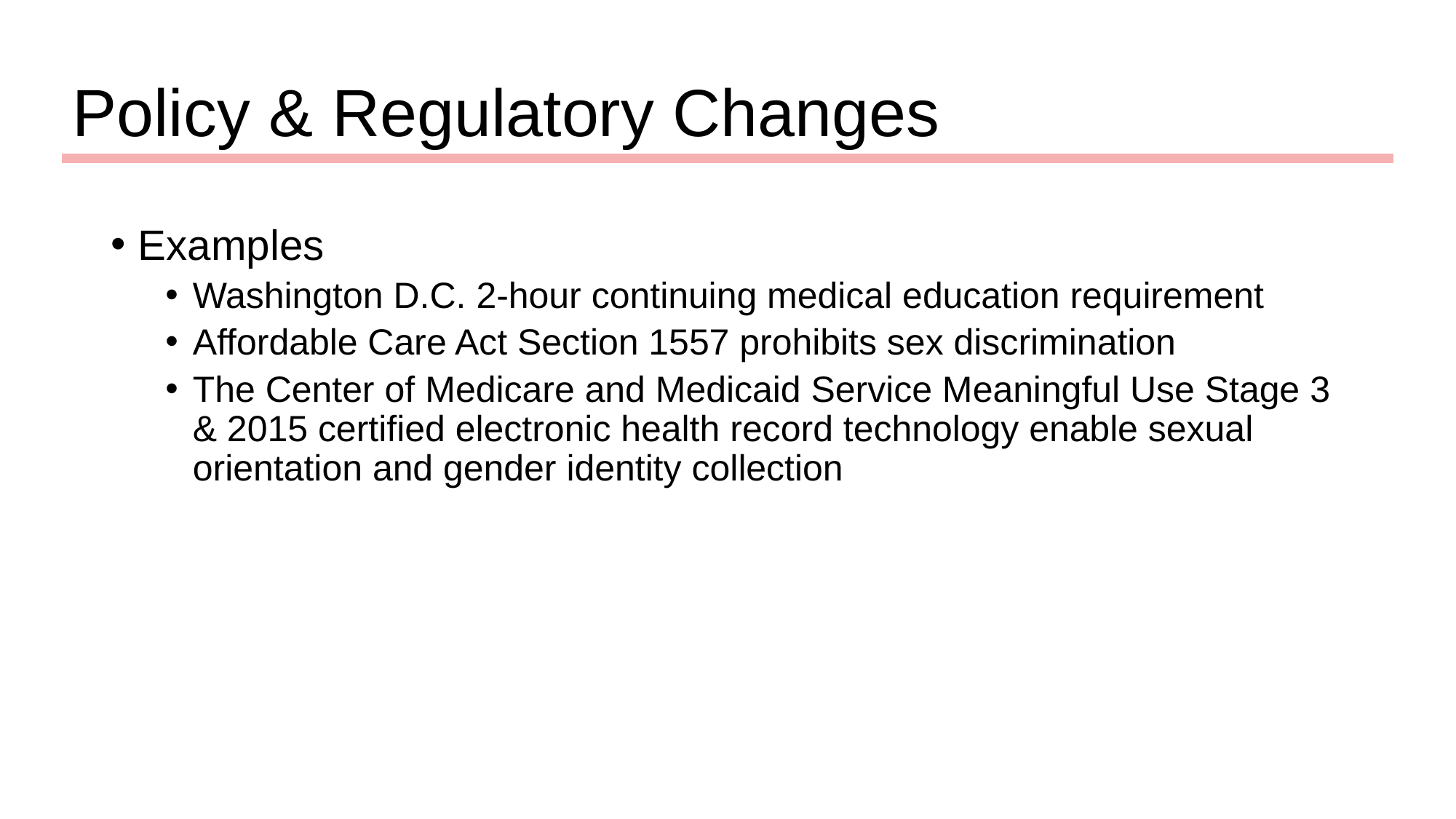

Policy & Regulatory Changes
Examples
Washington D.C. 2-hour continuing medical education requirement
Affordable Care Act Section 1557 prohibits sex discrimination
The Center of Medicare and Medicaid Service Meaningful Use Stage 3 & 2015 certified electronic health record technology enable sexual orientation and gender identity collection

## Slide 10
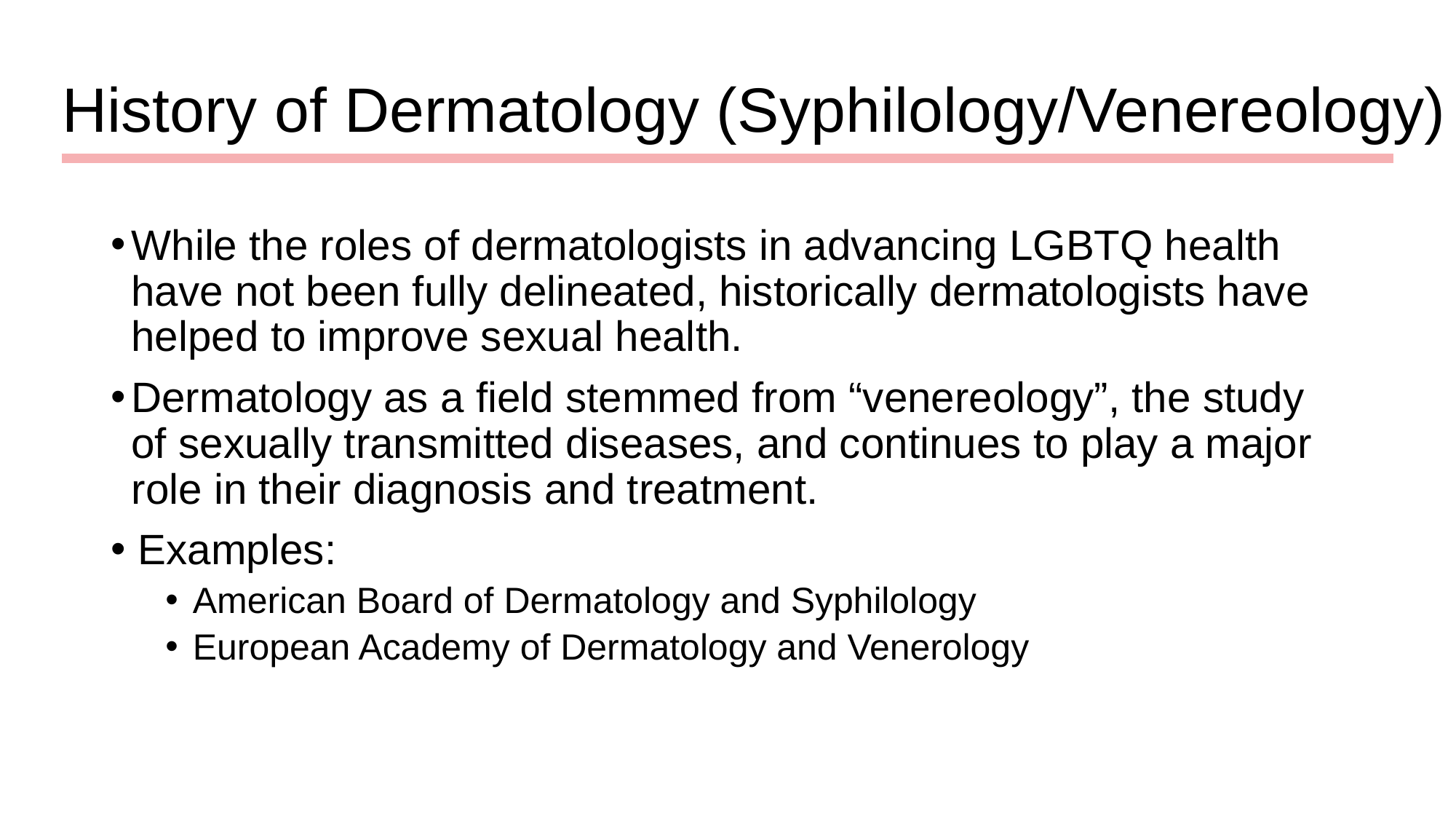

History of Dermatology (Syphilology/Venereology)
While the roles of dermatologists in advancing LGBTQ health have not been fully delineated, historically dermatologists have helped to improve sexual health.
Dermatology as a field stemmed from “venereology”, the study of sexually transmitted diseases, and continues to play a major role in their diagnosis and treatment.
Examples:
American Board of Dermatology and Syphilology
European Academy of Dermatology and Venerology

## Slide 11
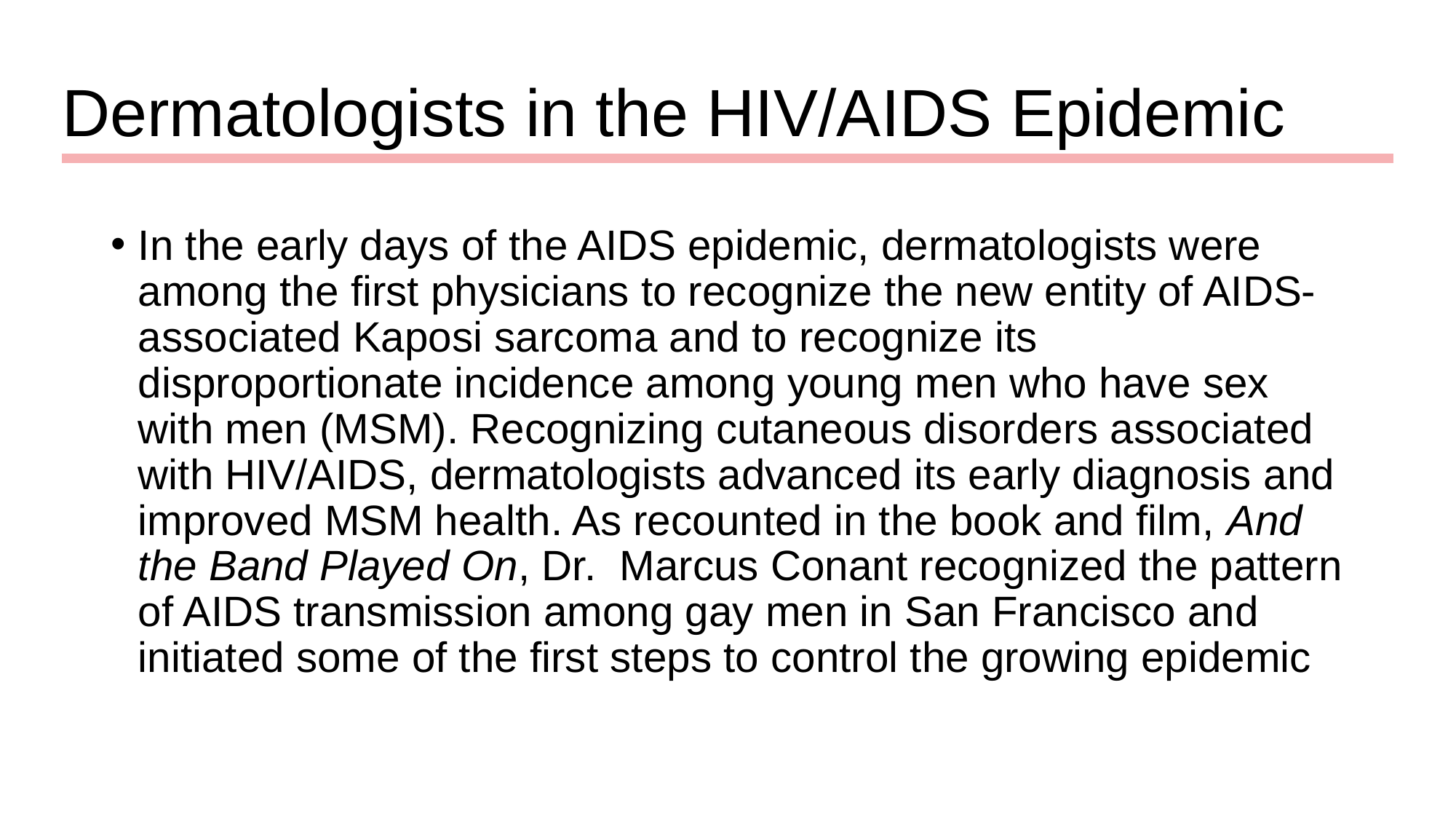

Dermatologists in the HIV/AIDS Epidemic
In the early days of the AIDS epidemic, dermatologists were among the first physicians to recognize the new entity of AIDS-associated Kaposi sarcoma and to recognize its disproportionate incidence among young men who have sex with men (MSM). Recognizing cutaneous disorders associated with HIV/AIDS, dermatologists advanced its early diagnosis and improved MSM health. As recounted in the book and film, And the Band Played On, Dr. Marcus Conant recognized the pattern of AIDS transmission among gay men in San Francisco and initiated some of the first steps to control the growing epidemic

## Slide 12
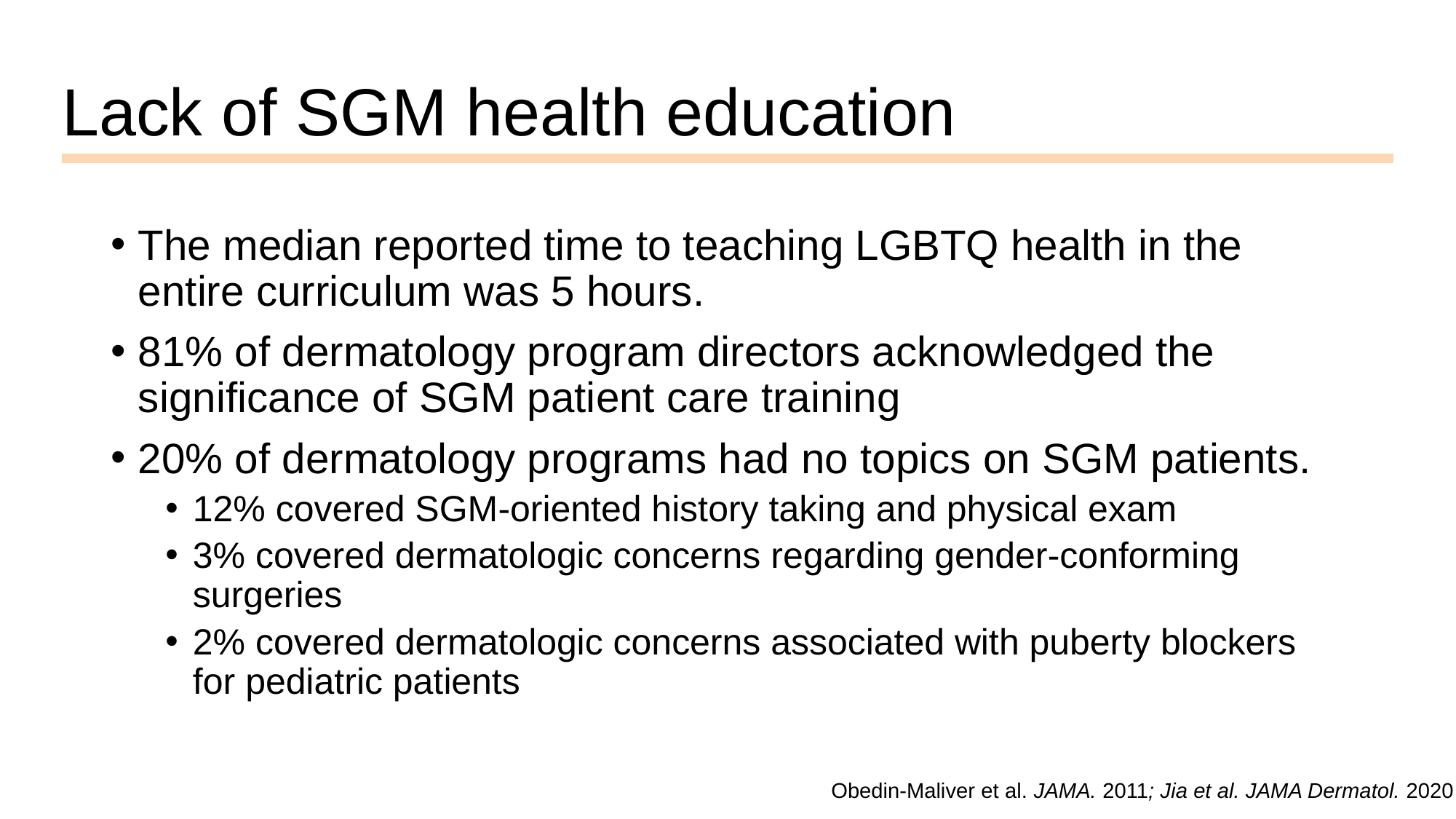

Lack of SGM health education
The median reported time to teaching LGBTQ health in the entire curriculum was 5 hours.
81% of dermatology program directors acknowledged the significance of SGM patient care training
20% of dermatology programs had no topics on SGM patients.
12% covered SGM-oriented history taking and physical exam
3% covered dermatologic concerns regarding gender-conforming surgeries
2% covered dermatologic concerns associated with puberty blockers for pediatric patients
Obedin-Maliver et al. JAMA. 2011; Jia et al. JAMA Dermatol. 2020

## Slide 13
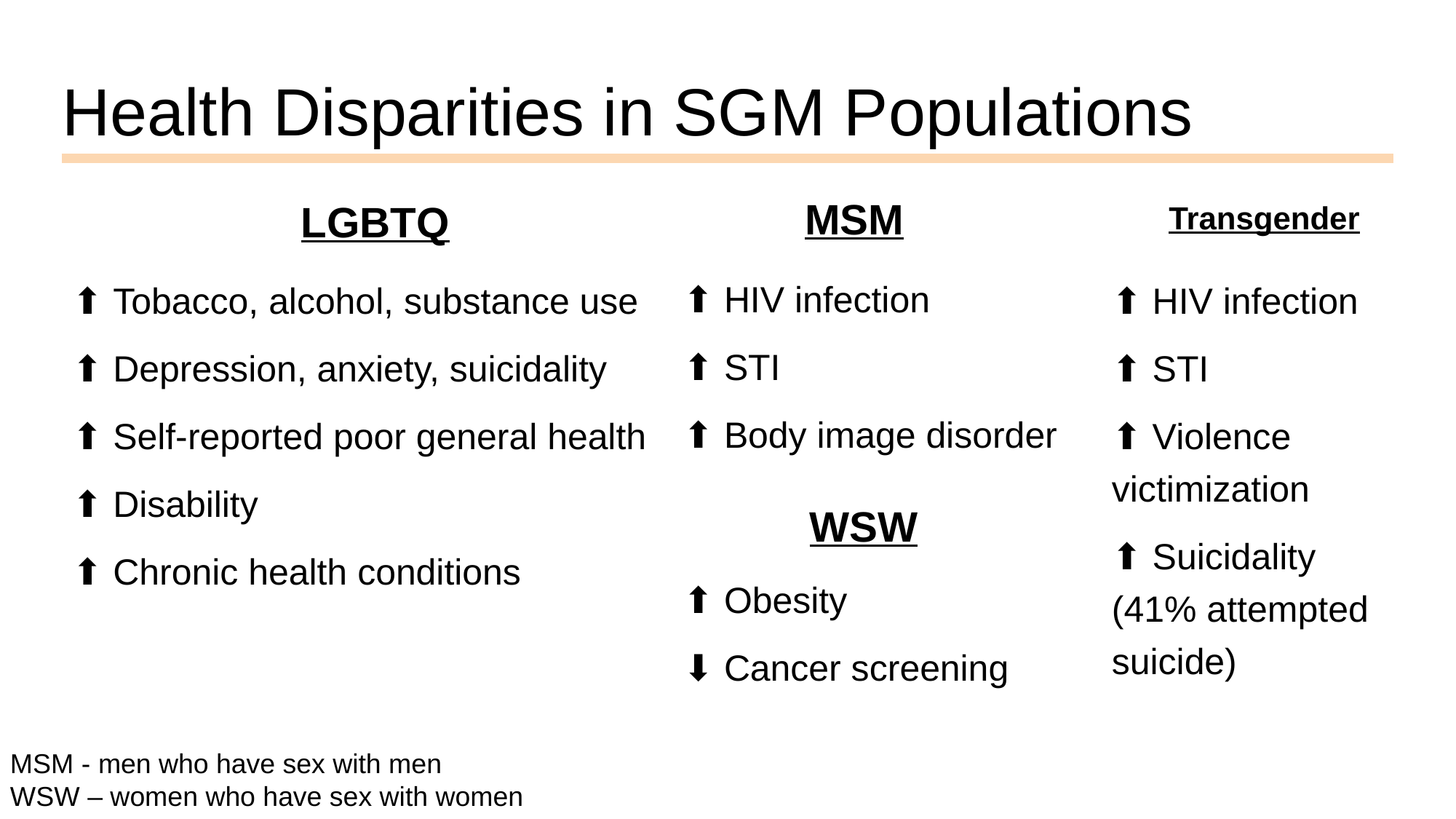

Health Disparities in SGM Populations
LGBTQ
MSM
Transgender
⬆️ HIV infection
⬆️ STI
⬆️ Body image disorder
⬆️ Tobacco, alcohol, substance use
⬆️ Depression, anxiety, suicidality
⬆️ Self-reported poor general health
⬆️ Disability
⬆️ Chronic health conditions
⬆️ HIV infection
⬆️ STI
⬆️ Violence victimization
⬆️ Suicidality
(41% attempted suicide)
WSW
⬆️ Obesity
⬇️ Cancer screening
MSM - men who have sex with men
WSW – women who have sex with women

## Slide 14
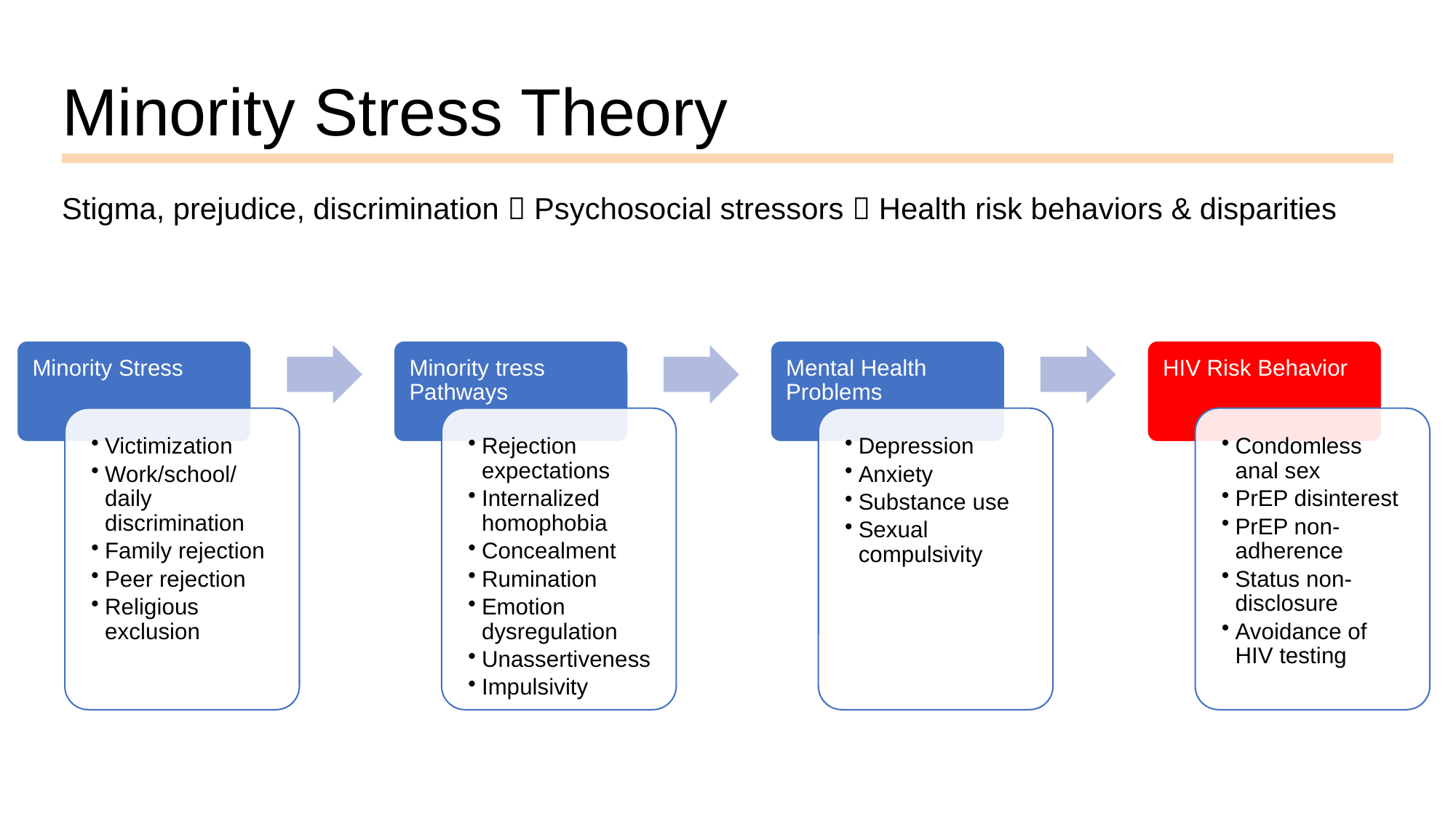

Minority Stress Theory
Stigma, prejudice, discrimination  Psychosocial stressors  Health risk behaviors & disparities

## Slide 15
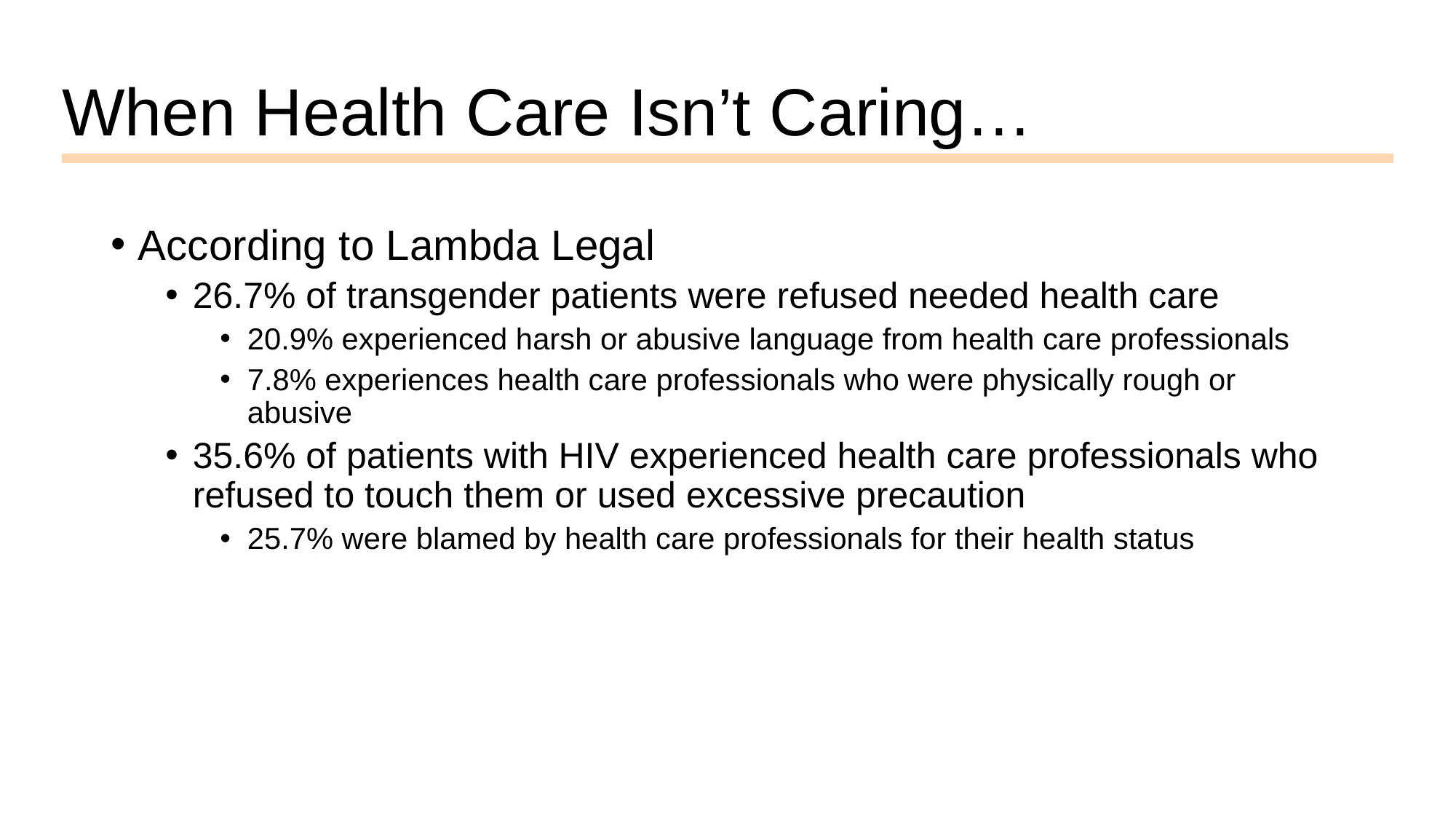

When Health Care Isn’t Caring…
According to Lambda Legal
26.7% of transgender patients were refused needed health care
20.9% experienced harsh or abusive language from health care professionals
7.8% experiences health care professionals who were physically rough or abusive
35.6% of patients with HIV experienced health care professionals who refused to touch them or used excessive precaution
25.7% were blamed by health care professionals for their health status

## Slide 16
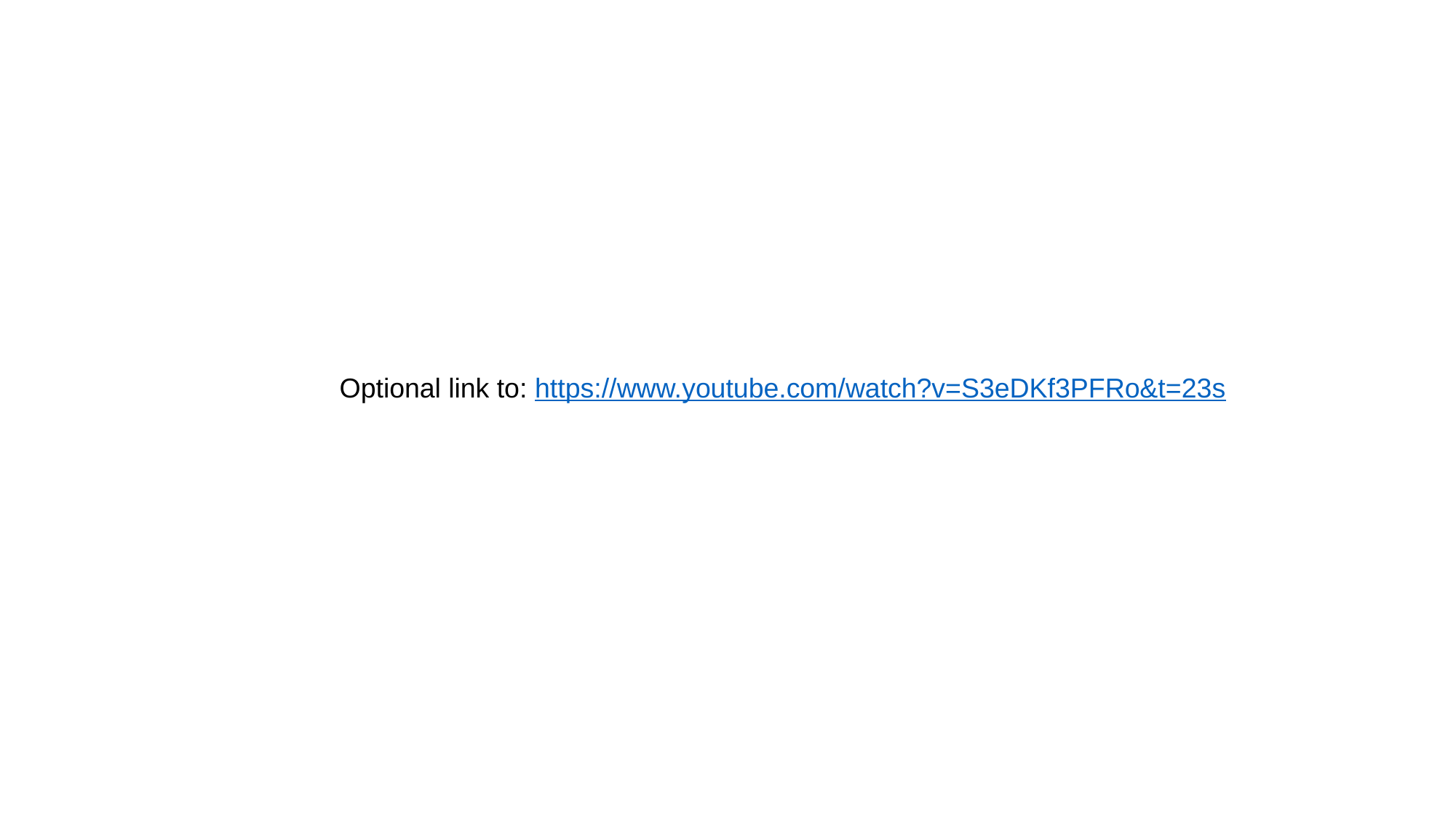

Optional link to: https://www.youtube.com/watch?v=S3eDKf3PFRo&t=23s

## Slide 17
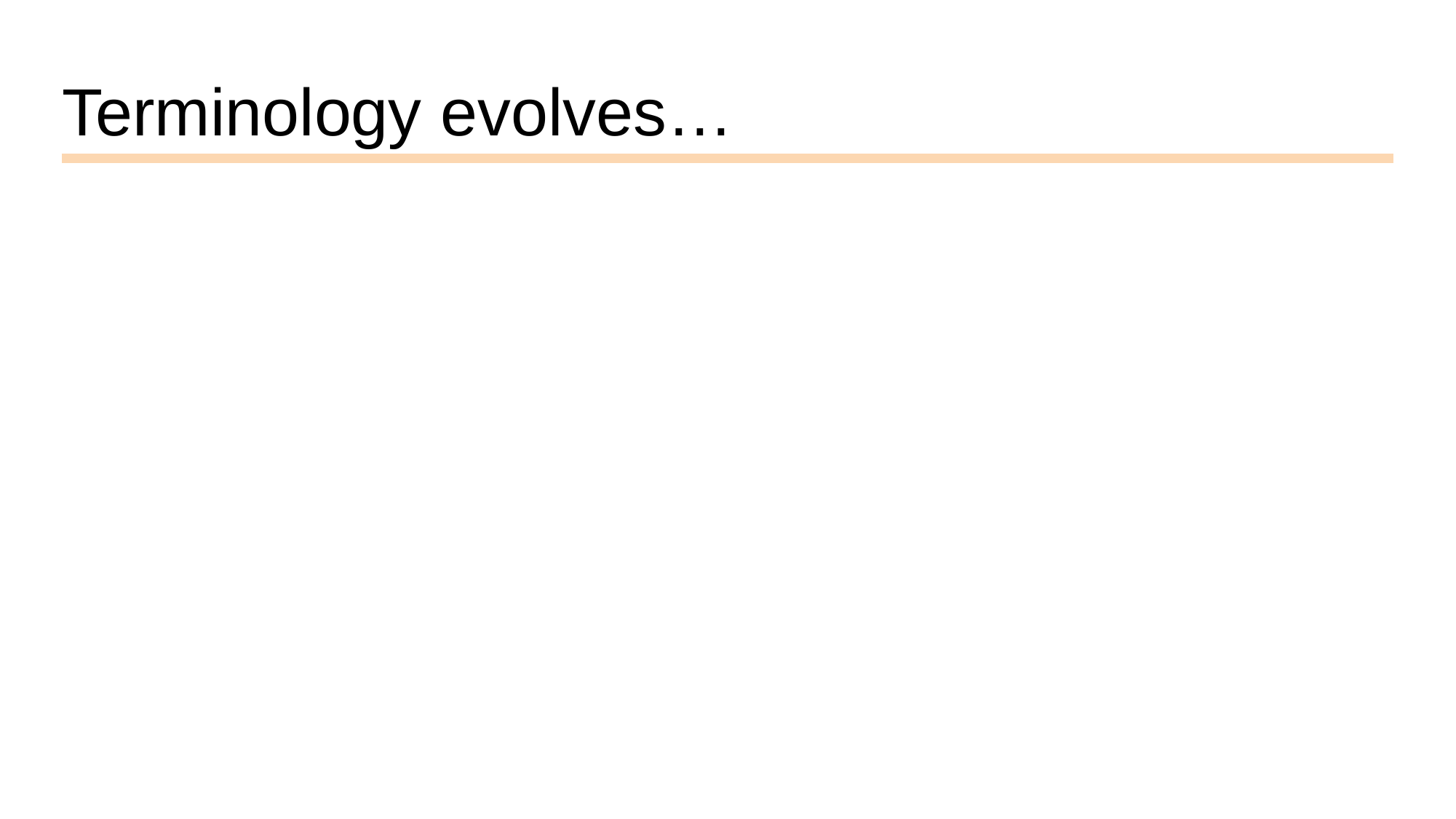

Terminology evolves…

## Slide 18
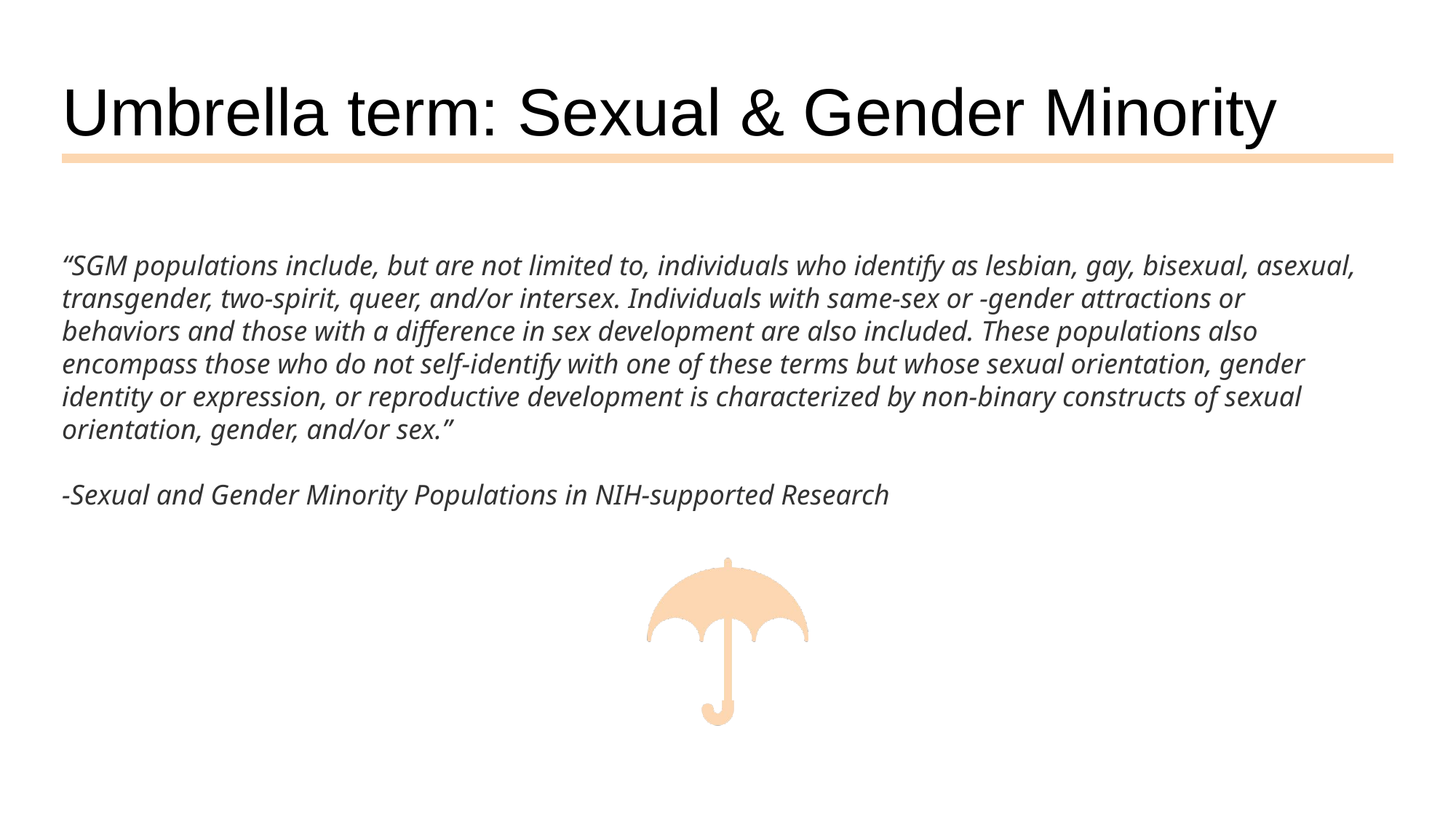

Umbrella term: Sexual & Gender Minority
“SGM populations include, but are not limited to, individuals who identify as lesbian, gay, bisexual, asexual, transgender, two-spirit, queer, and/or intersex. Individuals with same-sex or -gender attractions or behaviors and those with a difference in sex development are also included. These populations also encompass those who do not self-identify with one of these terms but whose sexual orientation, gender identity or expression, or reproductive development is characterized by non-binary constructs of sexual orientation, gender, and/or sex.”
-Sexual and Gender Minority Populations in NIH-supported Research

## Slide 19
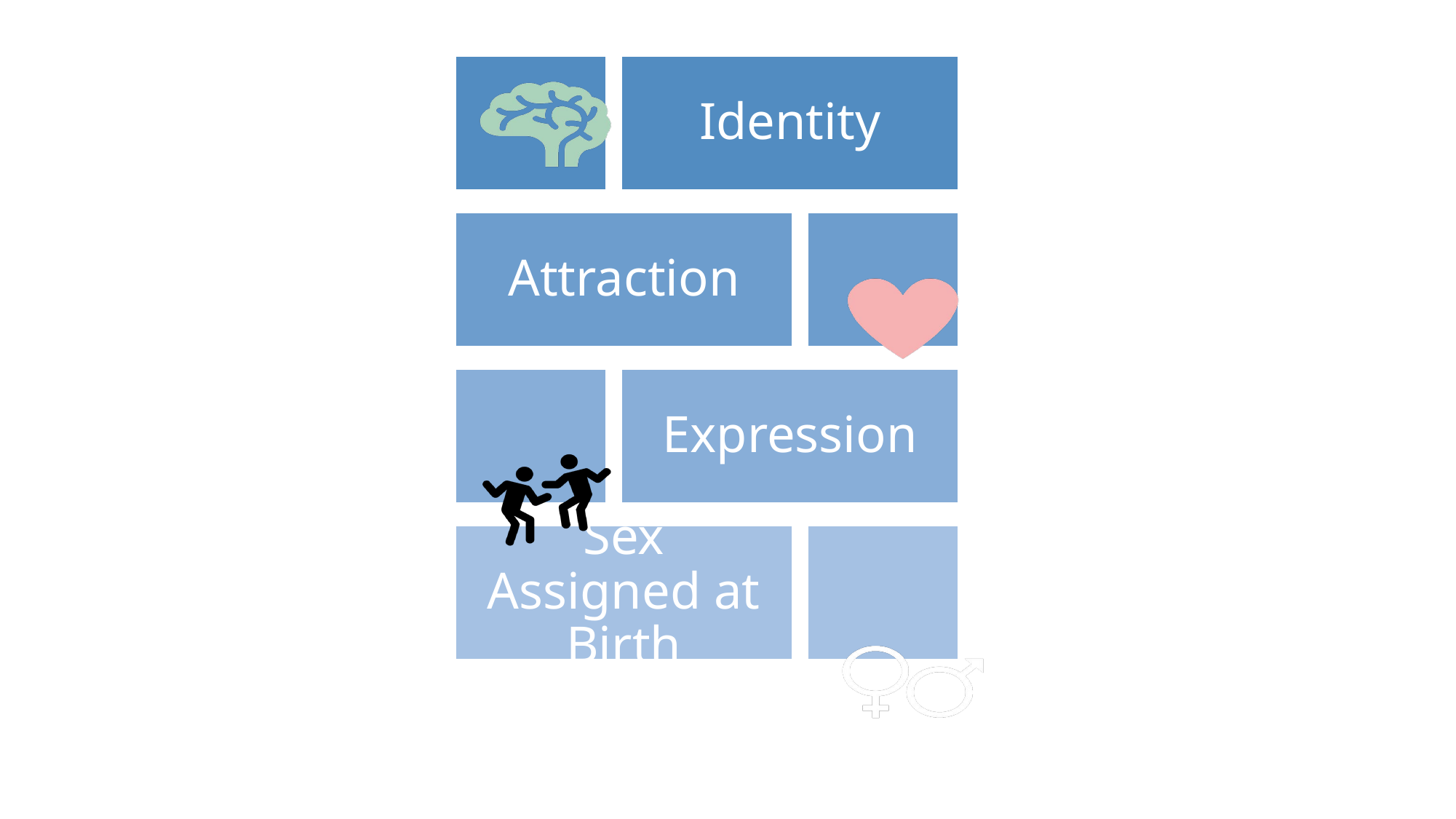

## Slide 20
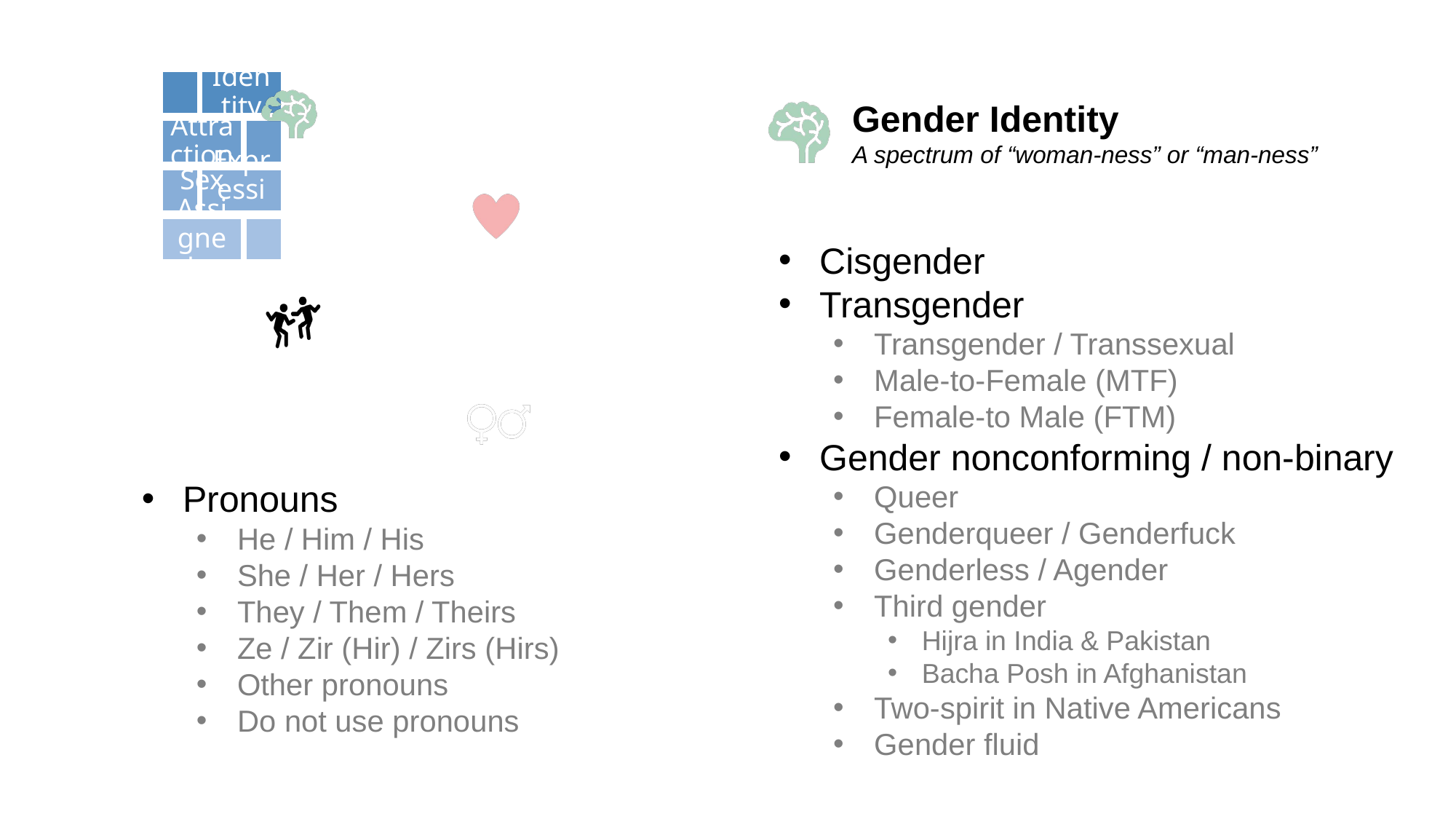

Gender Identity
A spectrum of “woman-ness” or “man-ness”
Cisgender
Transgender
Transgender / Transsexual
Male-to-Female (MTF)
Female-to Male (FTM)
Gender nonconforming / non-binary
Queer
Genderqueer / Genderfuck
Genderless / Agender
Third gender
Hijra in India & Pakistan
Bacha Posh in Afghanistan
Two-spirit in Native Americans
Gender fluid
Pronouns
He / Him / His
She / Her / Hers
They / Them / Theirs
Ze / Zir (Hir) / Zirs (Hirs)
Other pronouns
Do not use pronouns

## Slide 21
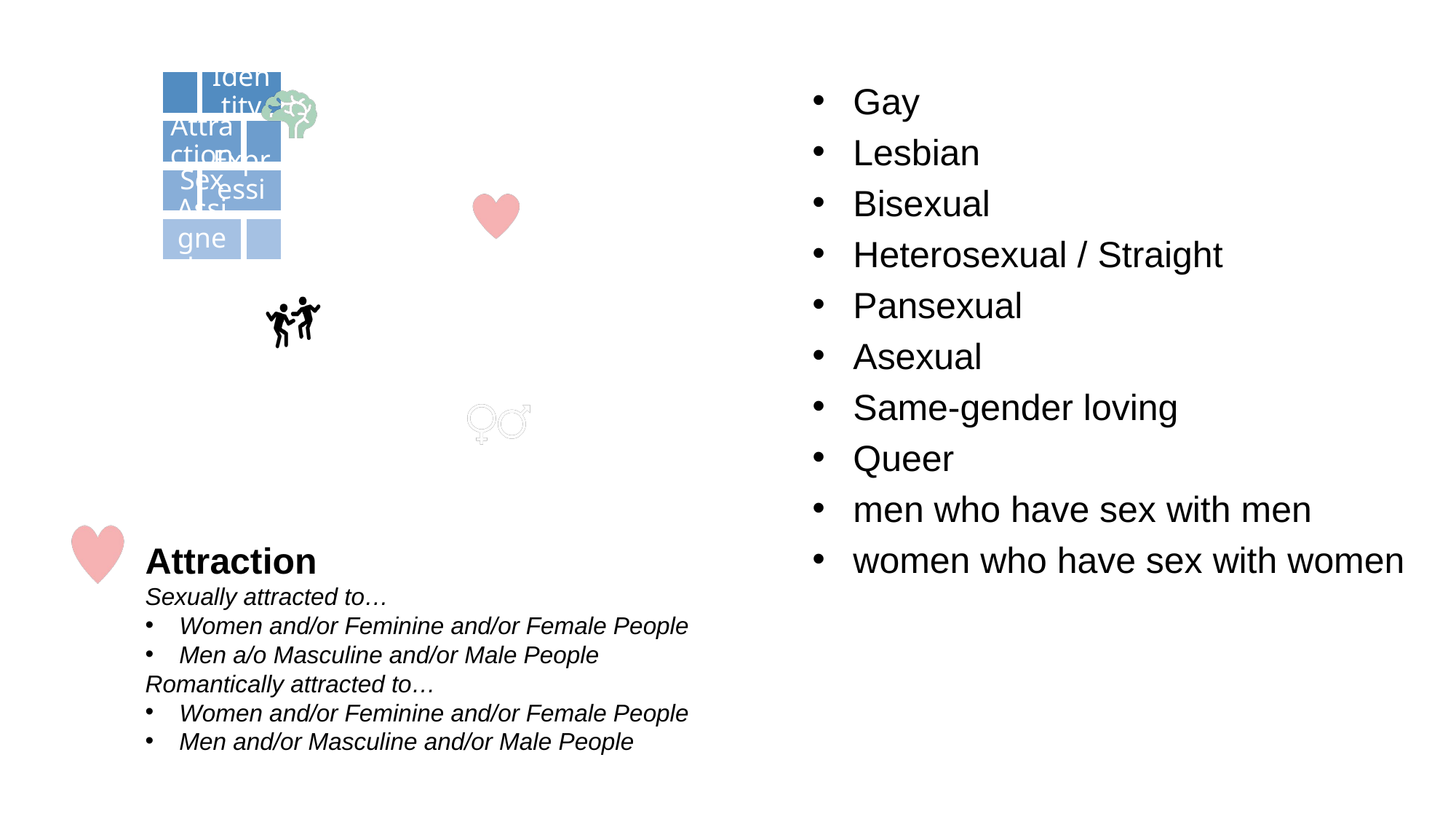

Gay
Lesbian
Bisexual
Heterosexual / Straight
Pansexual
Asexual
Same-gender loving
Queer
men who have sex with men
women who have sex with women
Attraction
Sexually attracted to…
Women and/or Feminine and/or Female People
Men a/o Masculine and/or Male People
Romantically attracted to…
Women and/or Feminine and/or Female People
Men and/or Masculine and/or Male People

## Slide 22
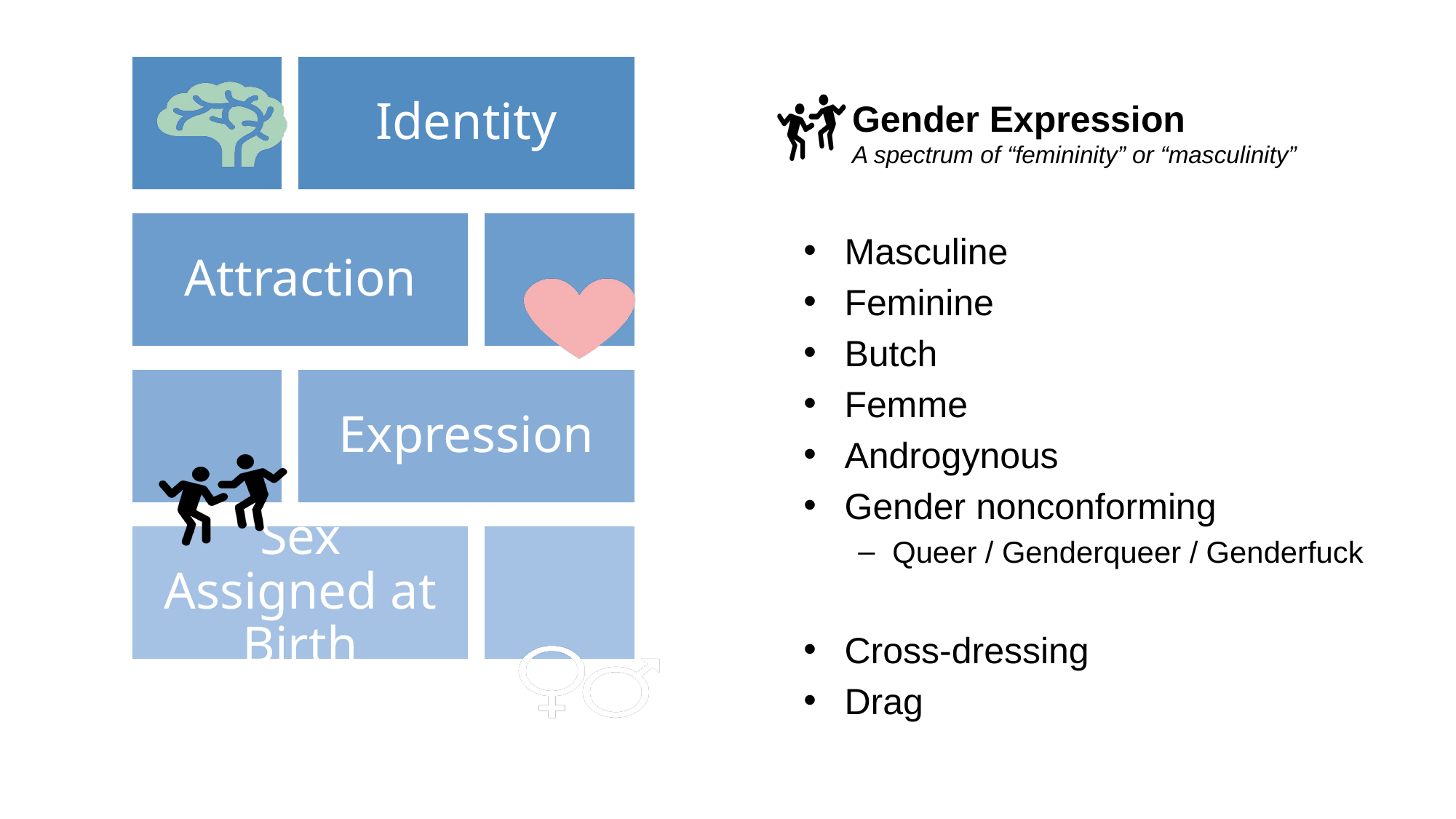

Gender Expression
A spectrum of “femininity” or “masculinity”
Masculine
Feminine
Butch
Femme
Androgynous
Gender nonconforming
Queer / Genderqueer / Genderfuck
Cross-dressing
Drag

## Slide 23
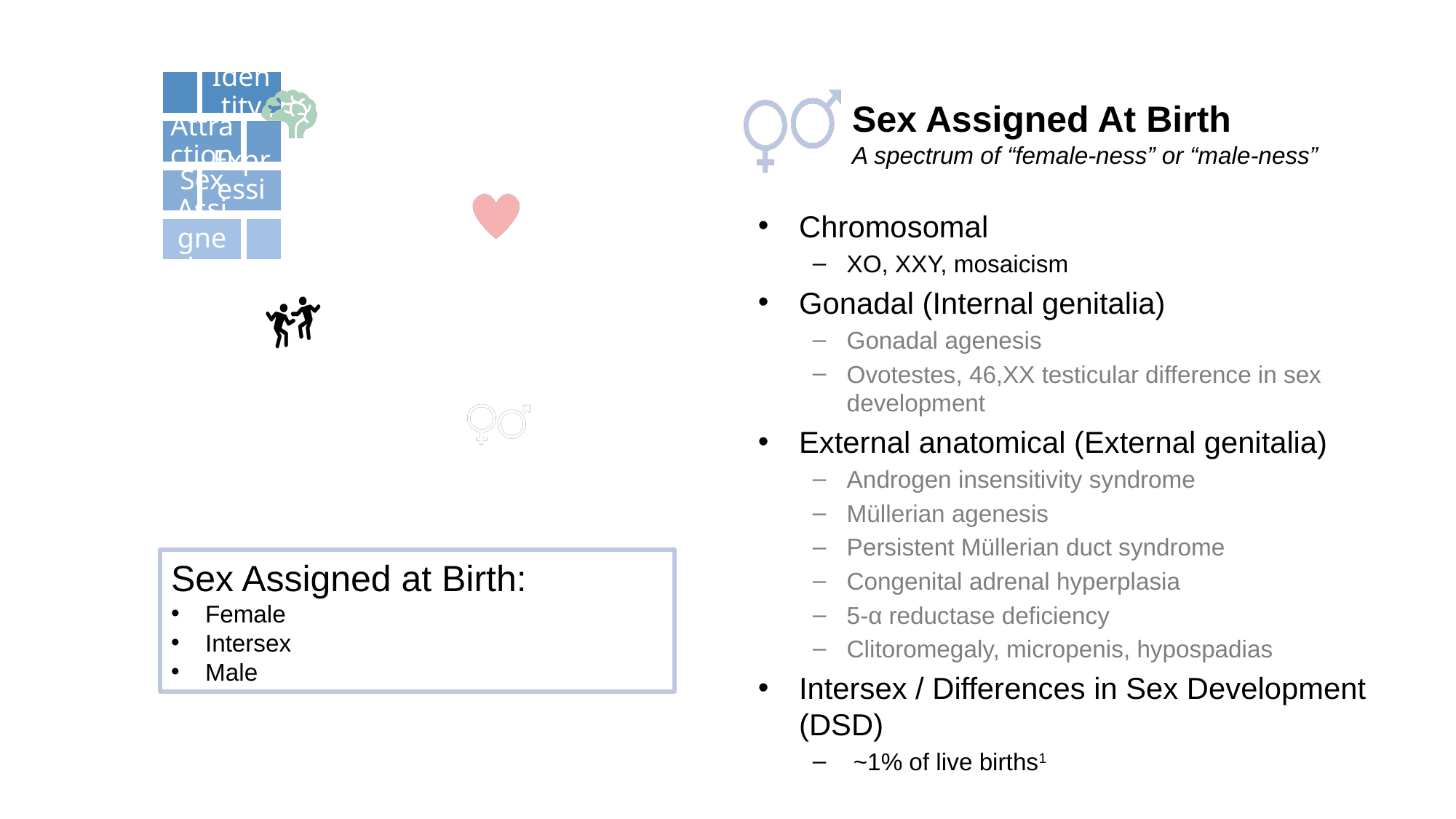

Sex Assigned At Birth
A spectrum of “female-ness” or “male-ness”
Chromosomal
XO, XXY, mosaicism
Gonadal (Internal genitalia)
Gonadal agenesis
Ovotestes, 46,XX testicular difference in sex development
External anatomical (External genitalia)
Androgen insensitivity syndrome
Müllerian agenesis
Persistent Müllerian duct syndrome
Congenital adrenal hyperplasia
5-α reductase deficiency
Clitoromegaly, micropenis, hypospadias
Intersex / Differences in Sex Development (DSD)
 ~1% of live births1
Sex Assigned at Birth:
Female
Intersex
Male

## Slide 24
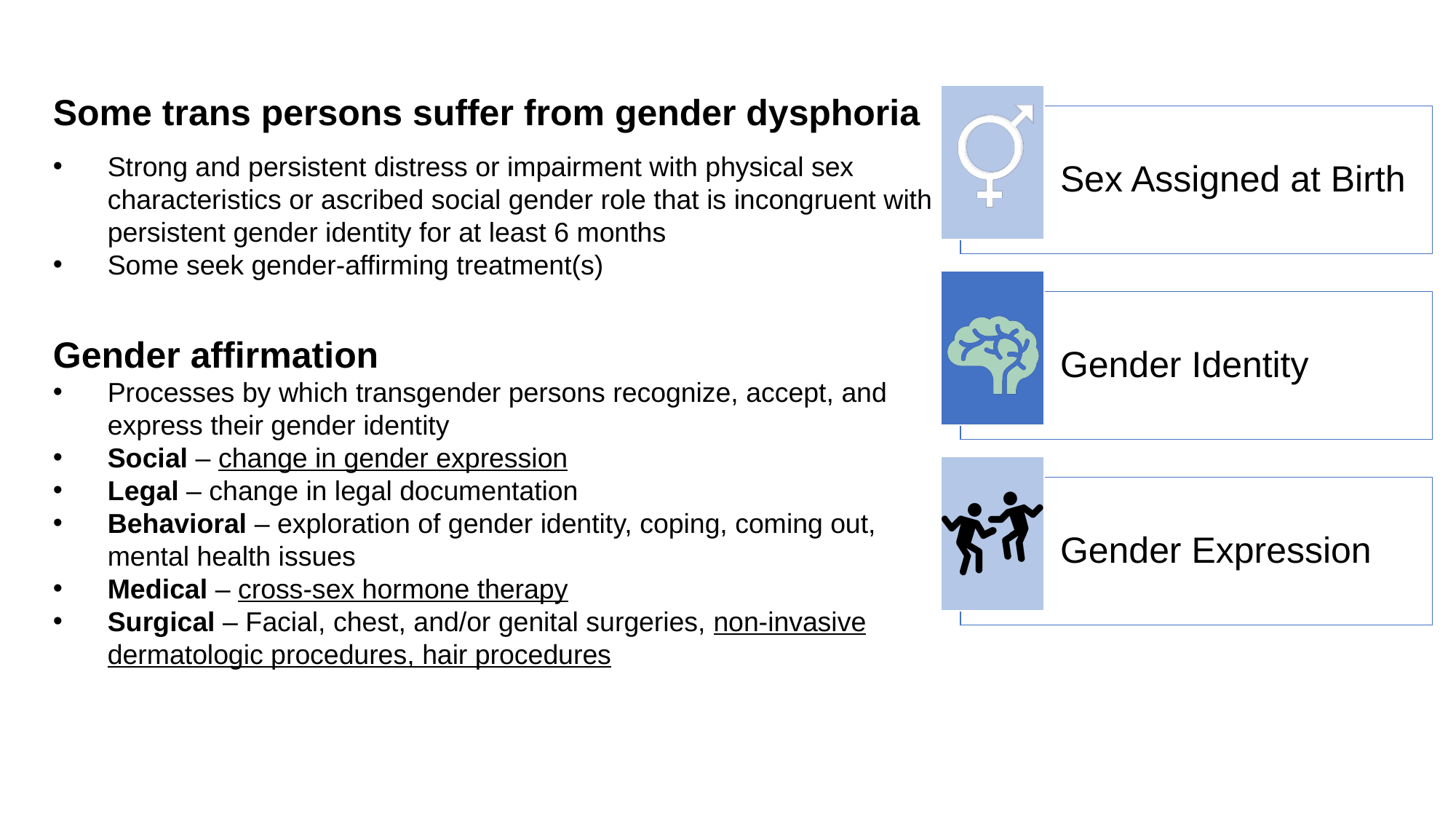

Some trans persons suffer from gender dysphoria
Strong and persistent distress or impairment with physical sex characteristics or ascribed social gender role that is incongruent with persistent gender identity for at least 6 months
Some seek gender-affirming treatment(s)
Gender affirmation
Processes by which transgender persons recognize, accept, and express their gender identity
Social – change in gender expression
Legal – change in legal documentation
Behavioral – exploration of gender identity, coping, coming out, mental health issues
Medical – cross-sex hormone therapy
Surgical – Facial, chest, and/or genital surgeries, non-invasive dermatologic procedures, hair procedures

## Slide 25
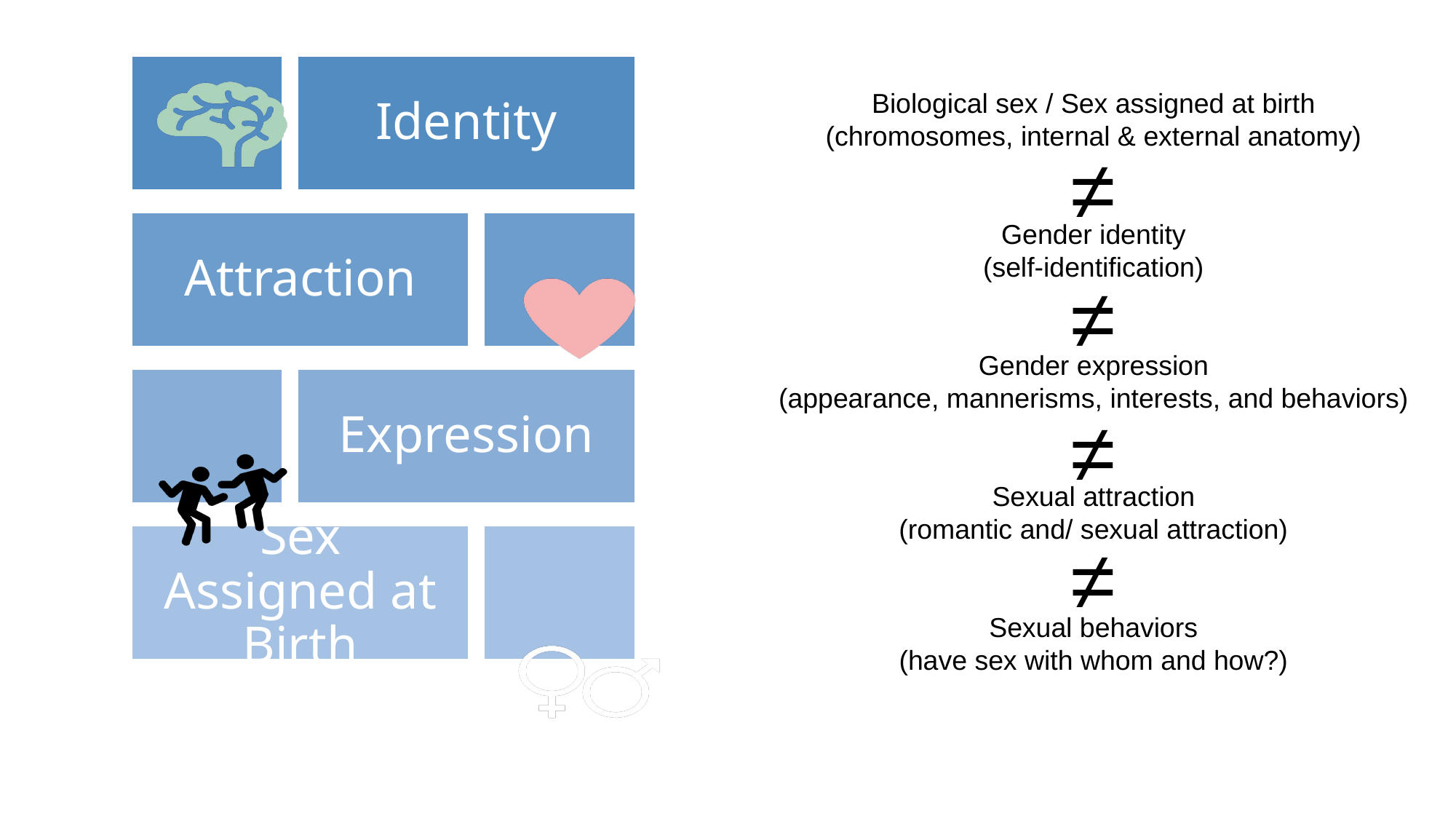

Biological sex / Sex assigned at birth
(chromosomes, internal & external anatomy)
Gender identity
(self-identification)
Gender expression
(appearance, mannerisms, interests, and behaviors)
Sexual attraction
(romantic and/ sexual attraction)
Sexual behaviors
(have sex with whom and how?)
≠
≠
≠
≠

## Slide 26
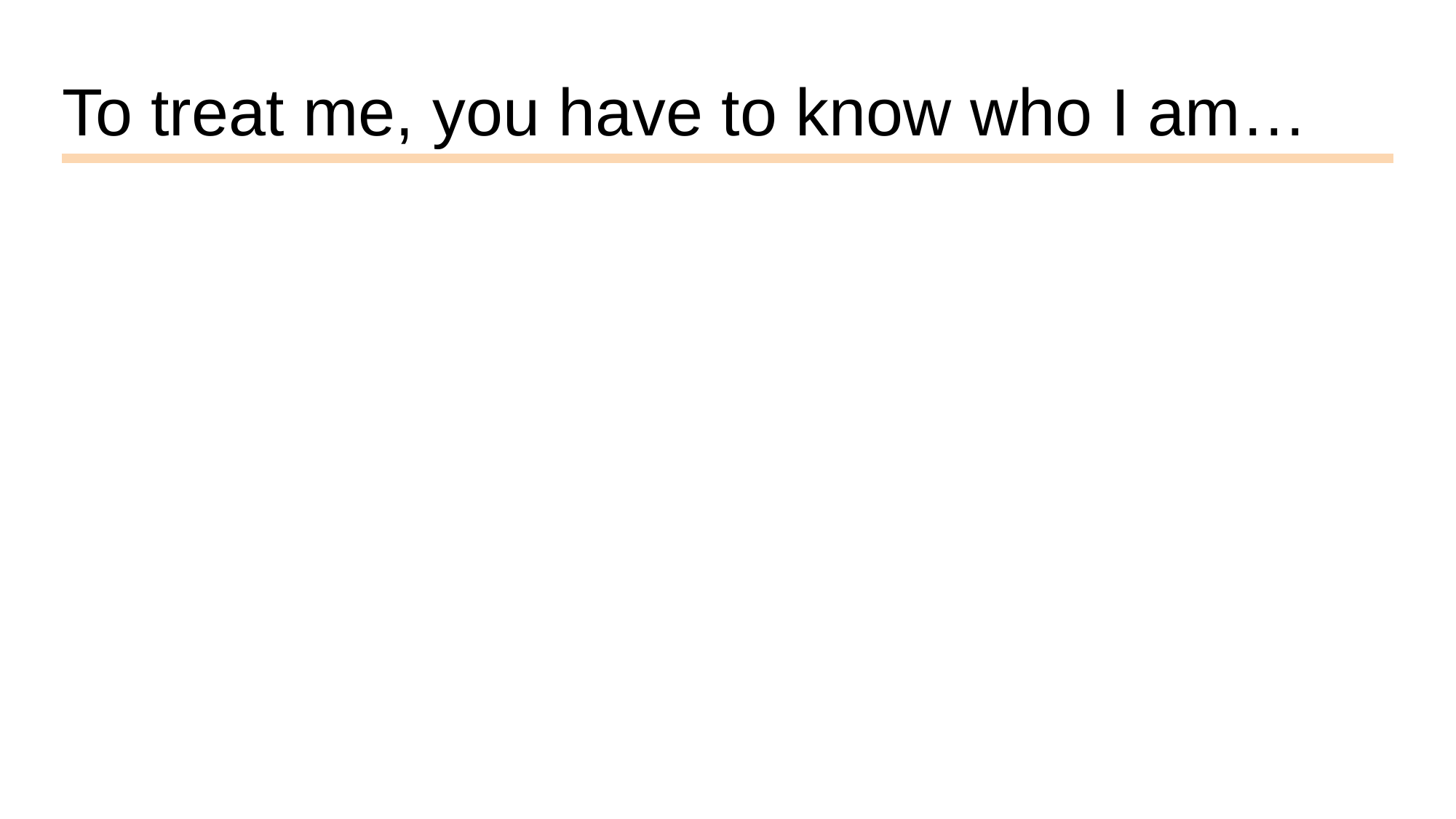

To treat me, you have to know who I am…

## Slide 27
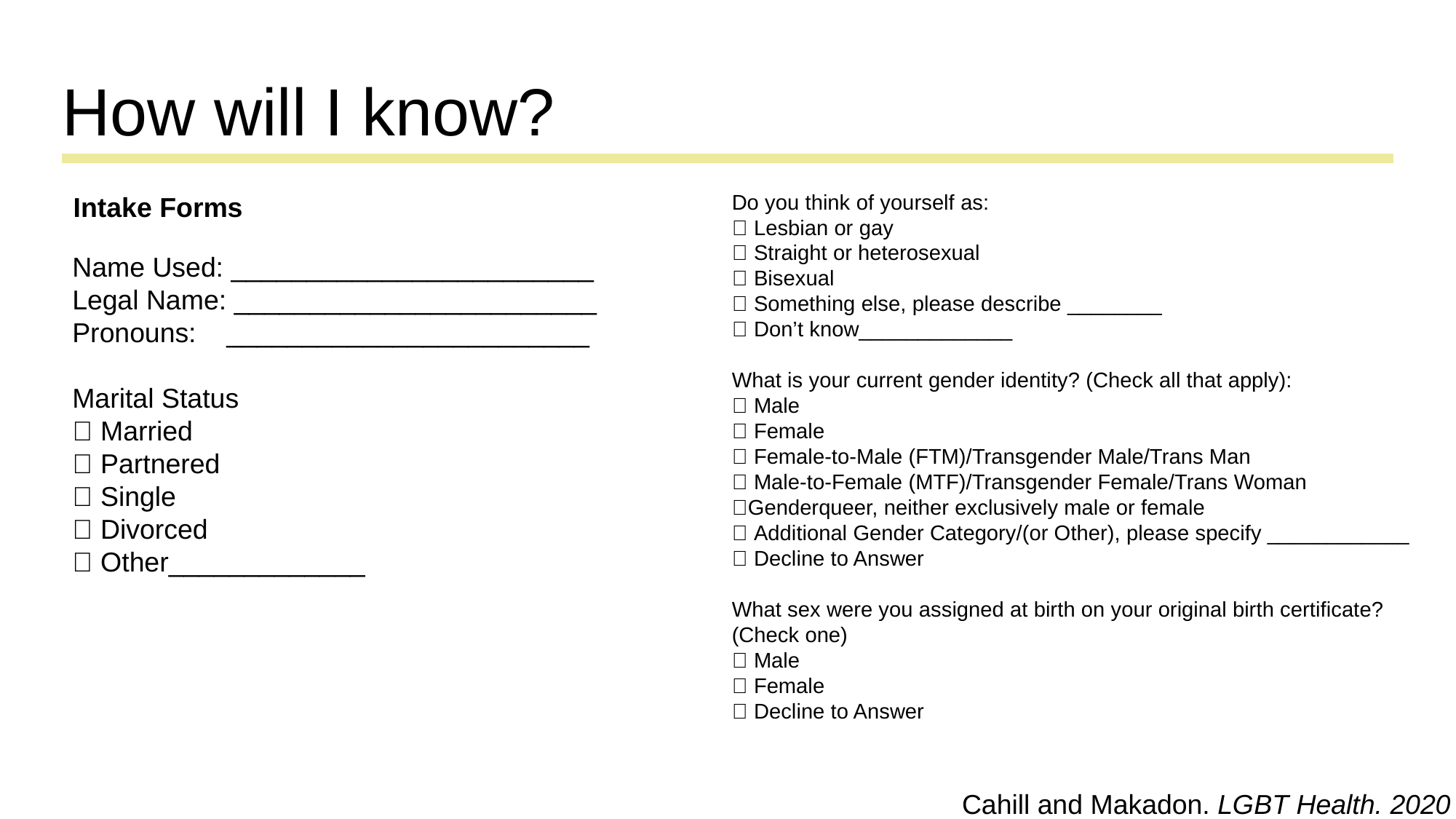

How will I know?
Do you think of yourself as:
 Lesbian or gay
 Straight or heterosexual
 Bisexual
 Something else, please describe ________
 Don’t know_____________
What is your current gender identity? (Check all that apply):
 Male
 Female
 Female-to-Male (FTM)/Transgender Male/Trans Man
 Male-to-Female (MTF)/Transgender Female/Trans Woman
Genderqueer, neither exclusively male or female
 Additional Gender Category/(or Other), please specify ____________
 Decline to Answer
What sex were you assigned at birth on your original birth certificate? (Check one)
 Male
 Female
 Decline to Answer
Intake Forms
Name Used: ________________________
Legal Name: ________________________
Pronouns: ________________________
Marital Status
 Married
 Partnered
 Single
 Divorced
 Other_____________
Cahill and Makadon. LGBT Health. 2020

## Slide 28
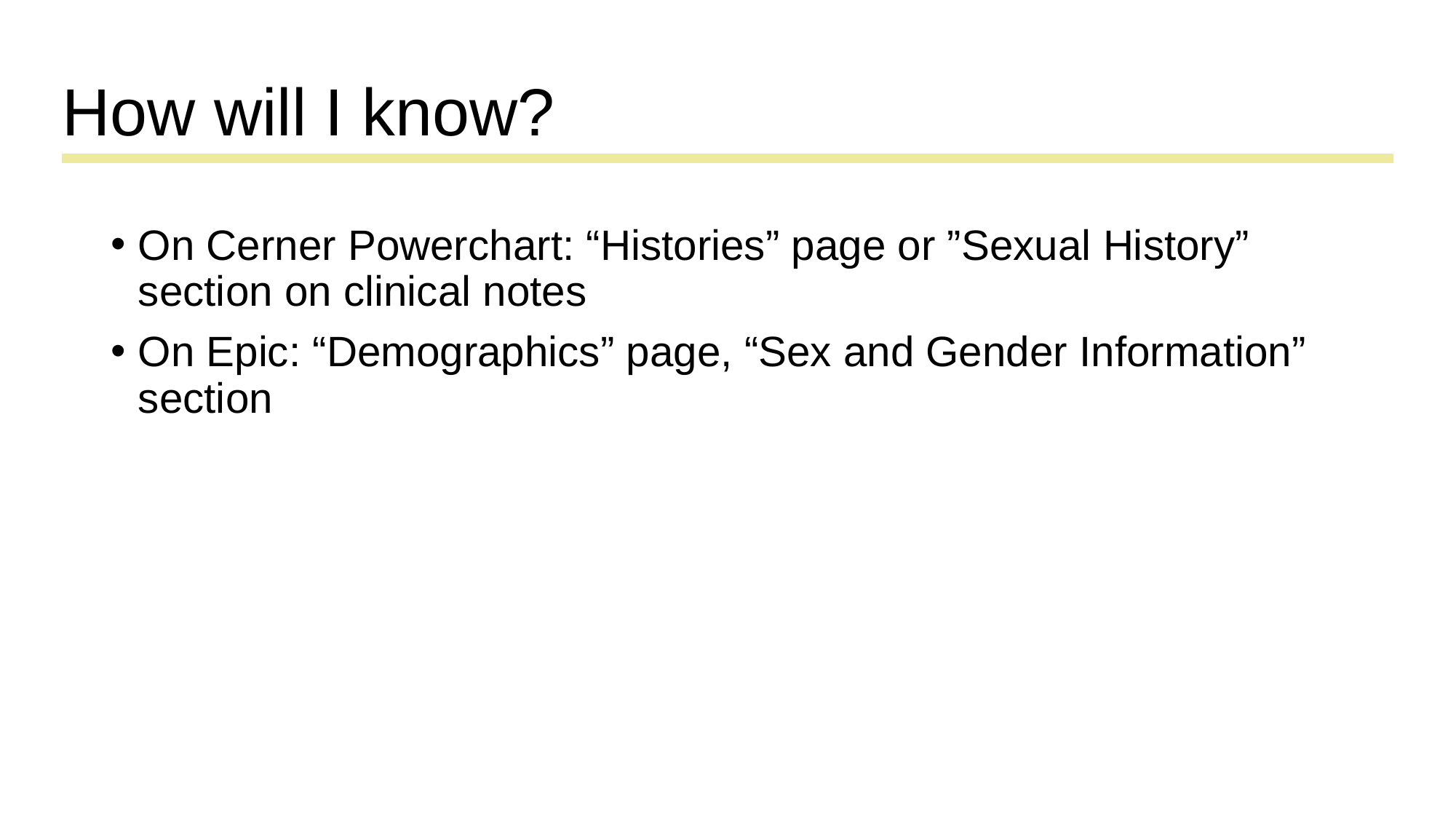

How will I know?
On Cerner Powerchart: “Histories” page or ”Sexual History” section on clinical notes
On Epic: “Demographics” page, “Sex and Gender Information” section

## Slide 29
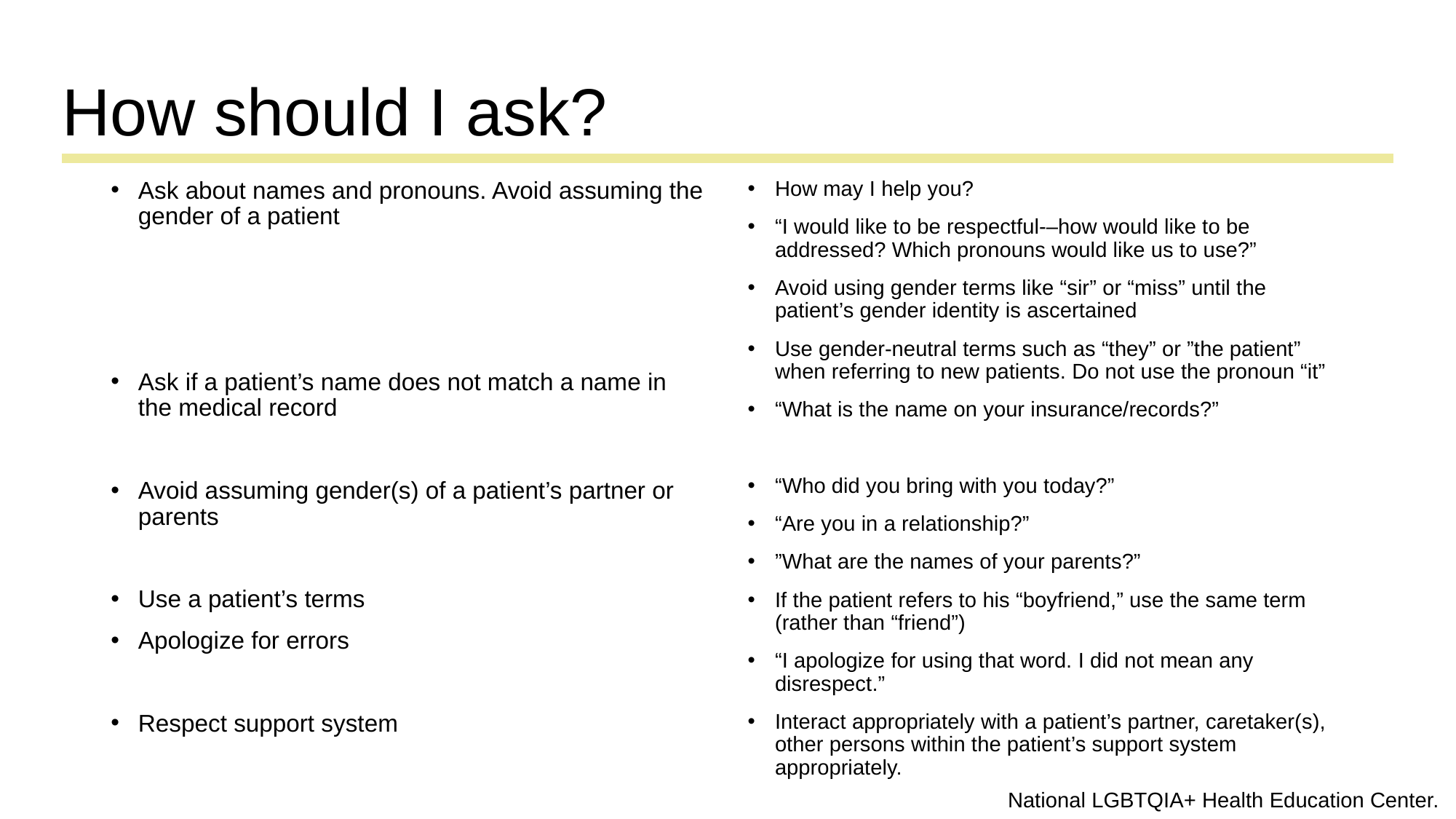

How should I ask?
Ask about names and pronouns. Avoid assuming the gender of a patient
Ask if a patient’s name does not match a name in the medical record
Avoid assuming gender(s) of a patient’s partner or parents
Use a patient’s terms
Apologize for errors
Respect support system
How may I help you?
“I would like to be respectful-–how would like to be addressed? Which pronouns would like us to use?”
Avoid using gender terms like “sir” or “miss” until the patient’s gender identity is ascertained
Use gender-neutral terms such as “they” or ”the patient” when referring to new patients. Do not use the pronoun “it”
“What is the name on your insurance/records?”
“Who did you bring with you today?”
“Are you in a relationship?”
”What are the names of your parents?”
If the patient refers to his “boyfriend,” use the same term (rather than “friend”)
“I apologize for using that word. I did not mean any disrespect.”
Interact appropriately with a patient’s partner, caretaker(s), other persons within the patient’s support system appropriately.
 National LGBTQIA+ Health Education Center.

## Slide 30
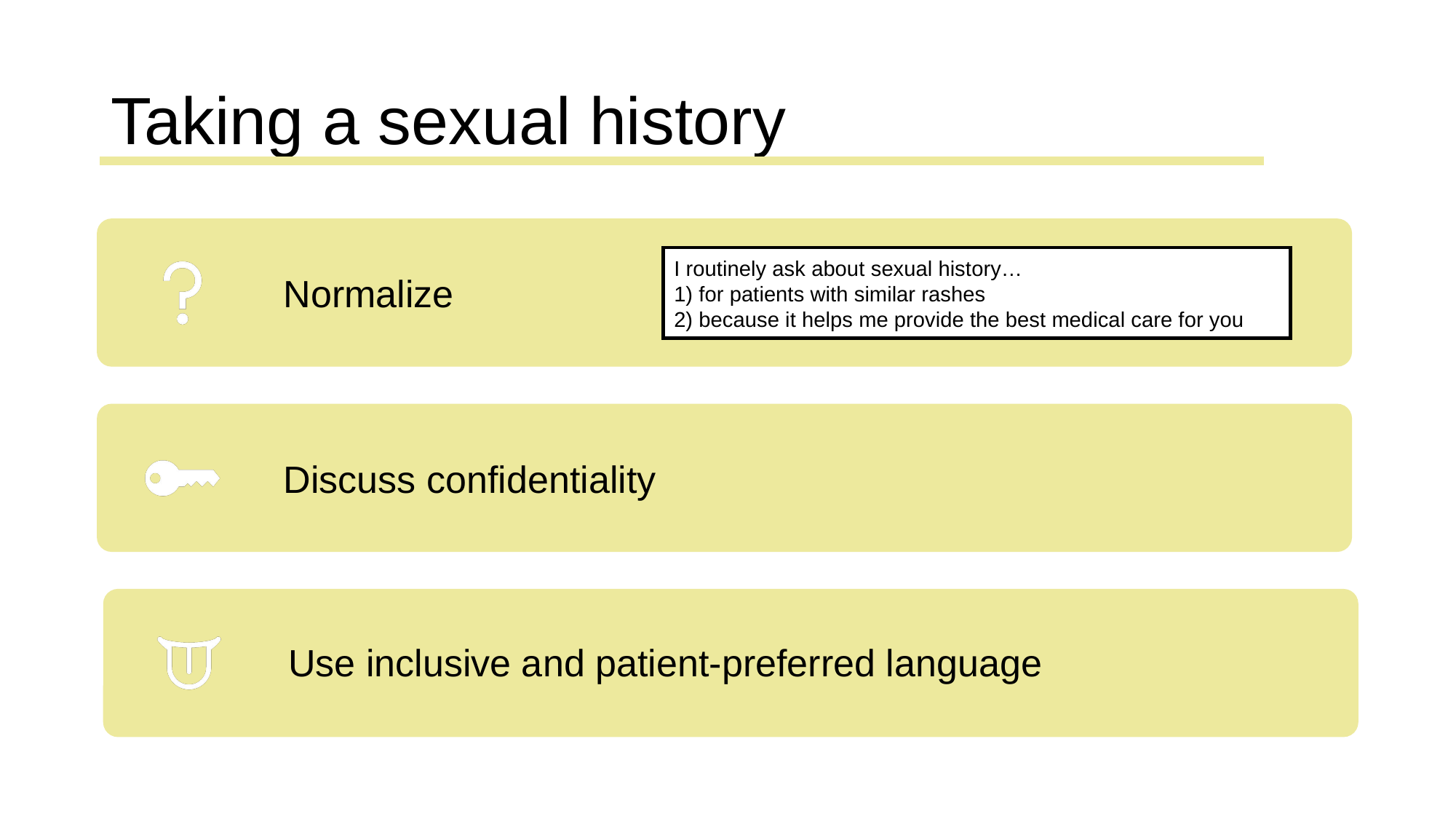

# Taking a sexual history
I routinely ask about sexual history…
1) for patients with similar rashes
2) because it helps me provide the best medical care for you

## Slide 31
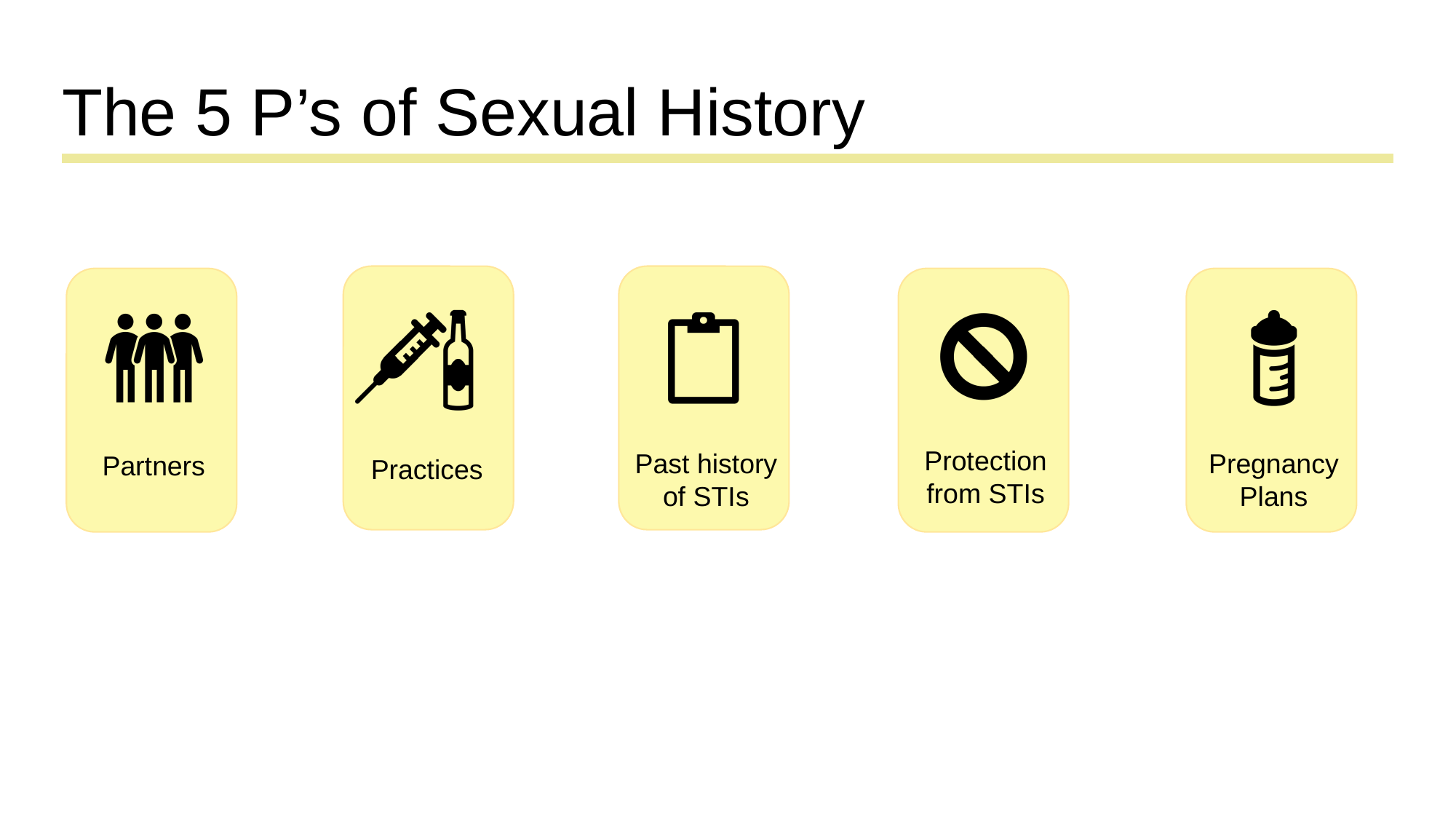

The 5 P’s of Sexual History
Protection from STIs
Past history
of STIs
Pregnancy Plans
Partners
Practices

## Slide 32
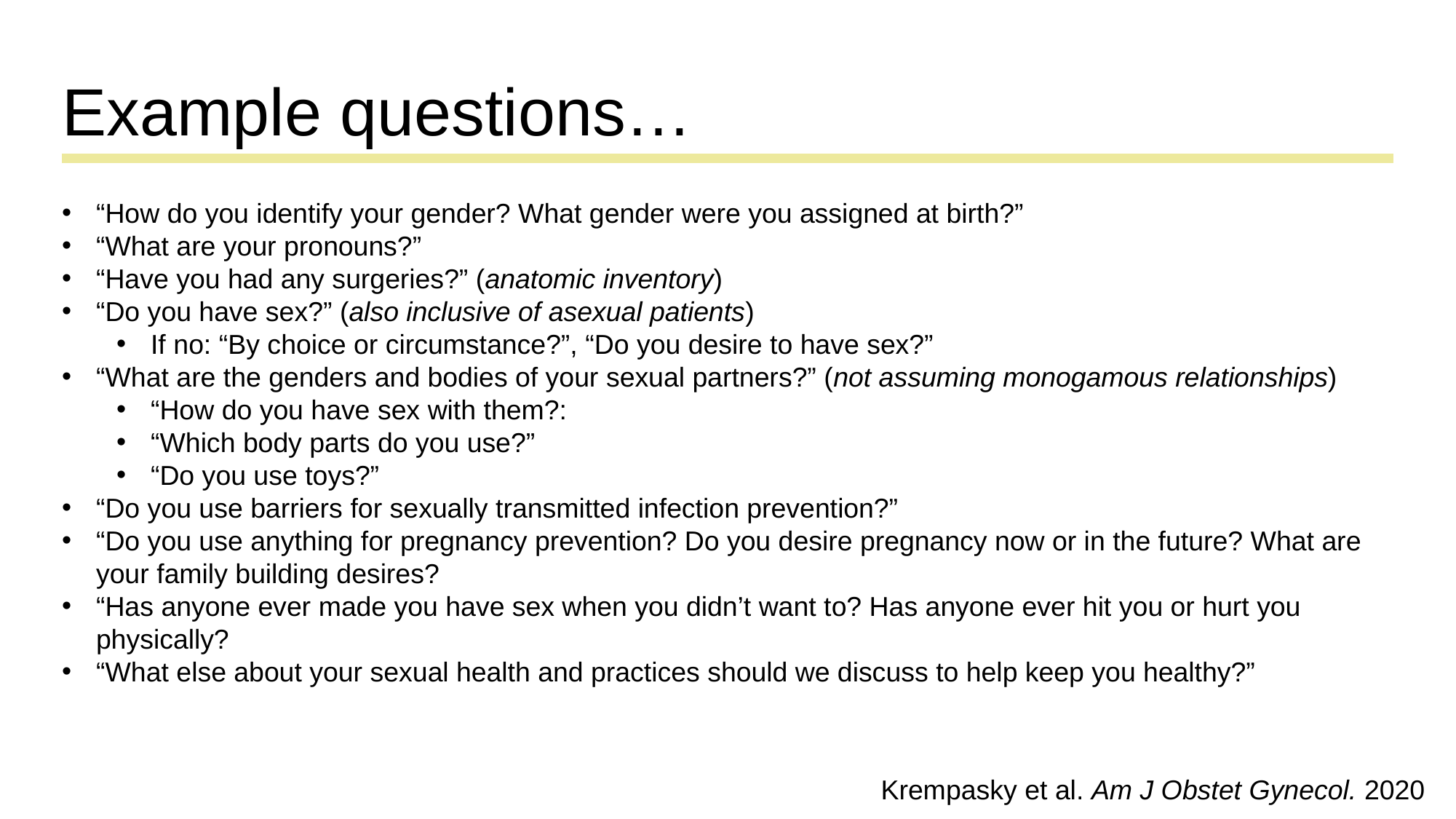

Example questions…
“How do you identify your gender? What gender were you assigned at birth?”
“What are your pronouns?”
“Have you had any surgeries?” (anatomic inventory)
“Do you have sex?” (also inclusive of asexual patients)
If no: “By choice or circumstance?”, “Do you desire to have sex?”
“What are the genders and bodies of your sexual partners?” (not assuming monogamous relationships)
“How do you have sex with them?:
“Which body parts do you use?”
“Do you use toys?”
“Do you use barriers for sexually transmitted infection prevention?”
“Do you use anything for pregnancy prevention? Do you desire pregnancy now or in the future? What are your family building desires?
“Has anyone ever made you have sex when you didn’t want to? Has anyone ever hit you or hurt you physically?
“What else about your sexual health and practices should we discuss to help keep you healthy?”
Krempasky et al. Am J Obstet Gynecol. 2020

## Slide 33
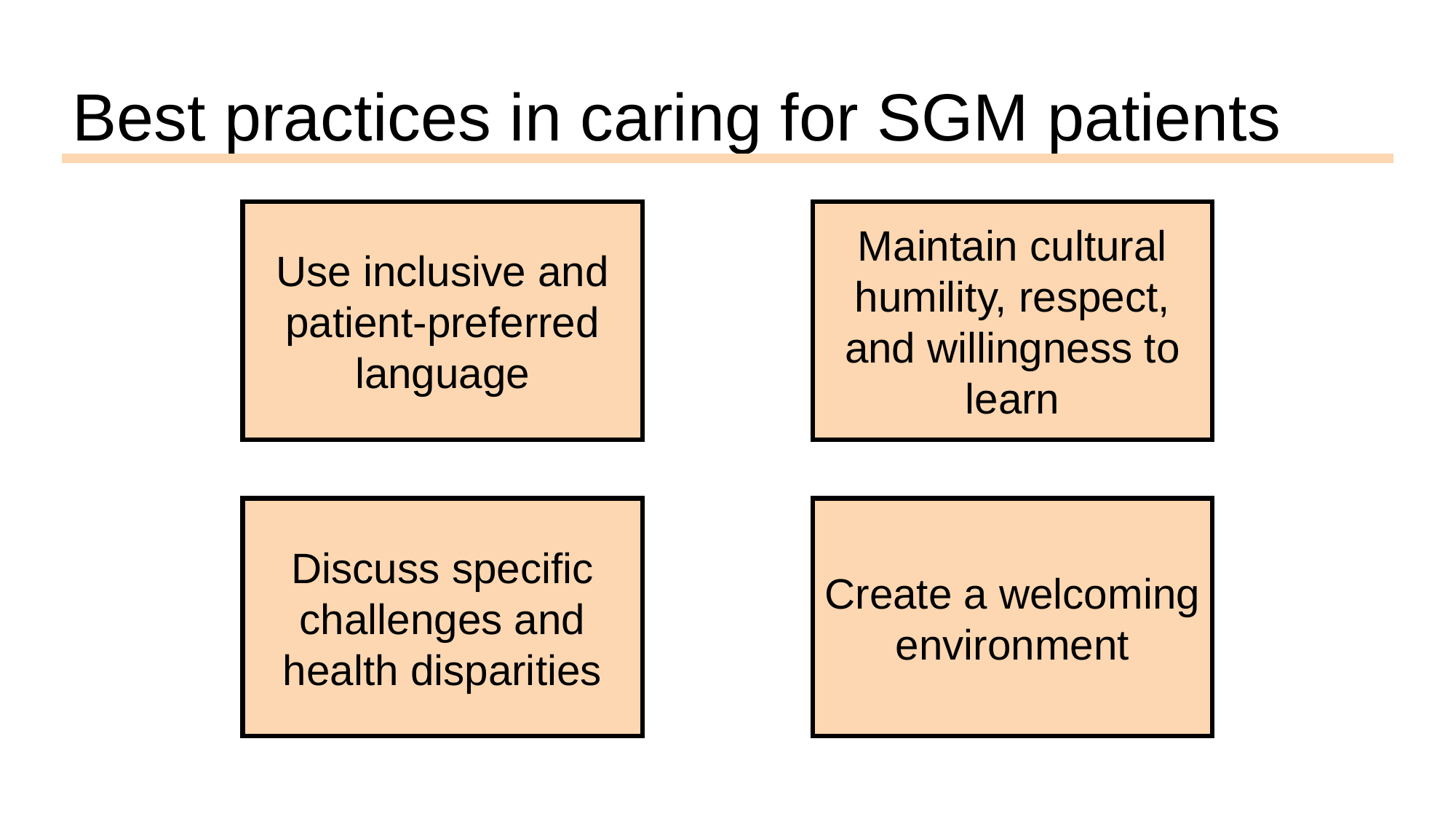

# Best practices in caring for SGM patients
Use inclusive and patient-preferred language
Maintain cultural humility, respect, and willingness to learn
Discuss specific challenges and health disparities
Create a welcoming environment

## Slide 34
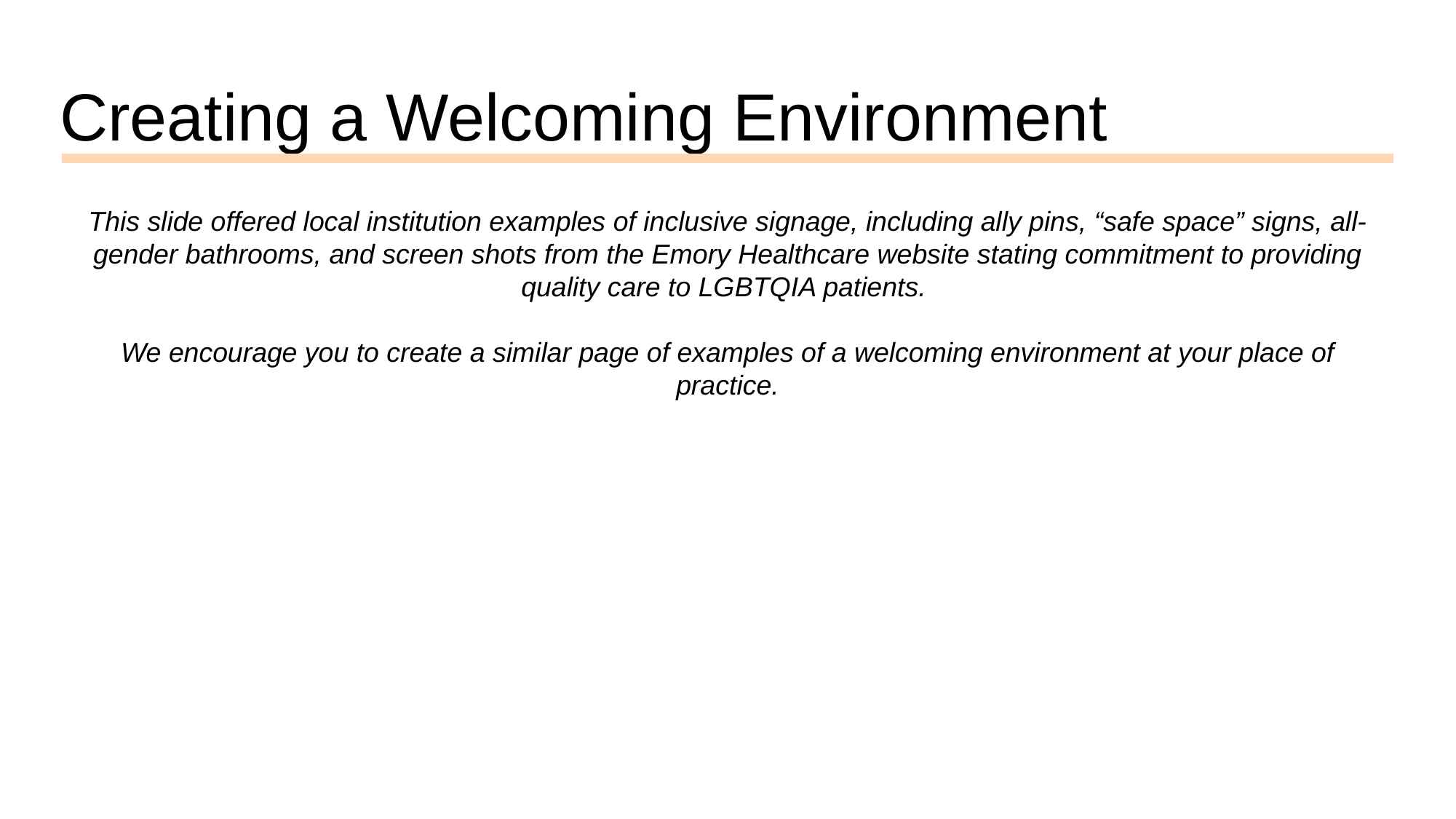

# Creating a Welcoming Environment
This slide offered local institution examples of inclusive signage, including ally pins, “safe space” signs, all-gender bathrooms, and screen shots from the Emory Healthcare website stating commitment to providing quality care to LGBTQIA patients.
We encourage you to create a similar page of examples of a welcoming environment at your place of practice.

## Slide 35
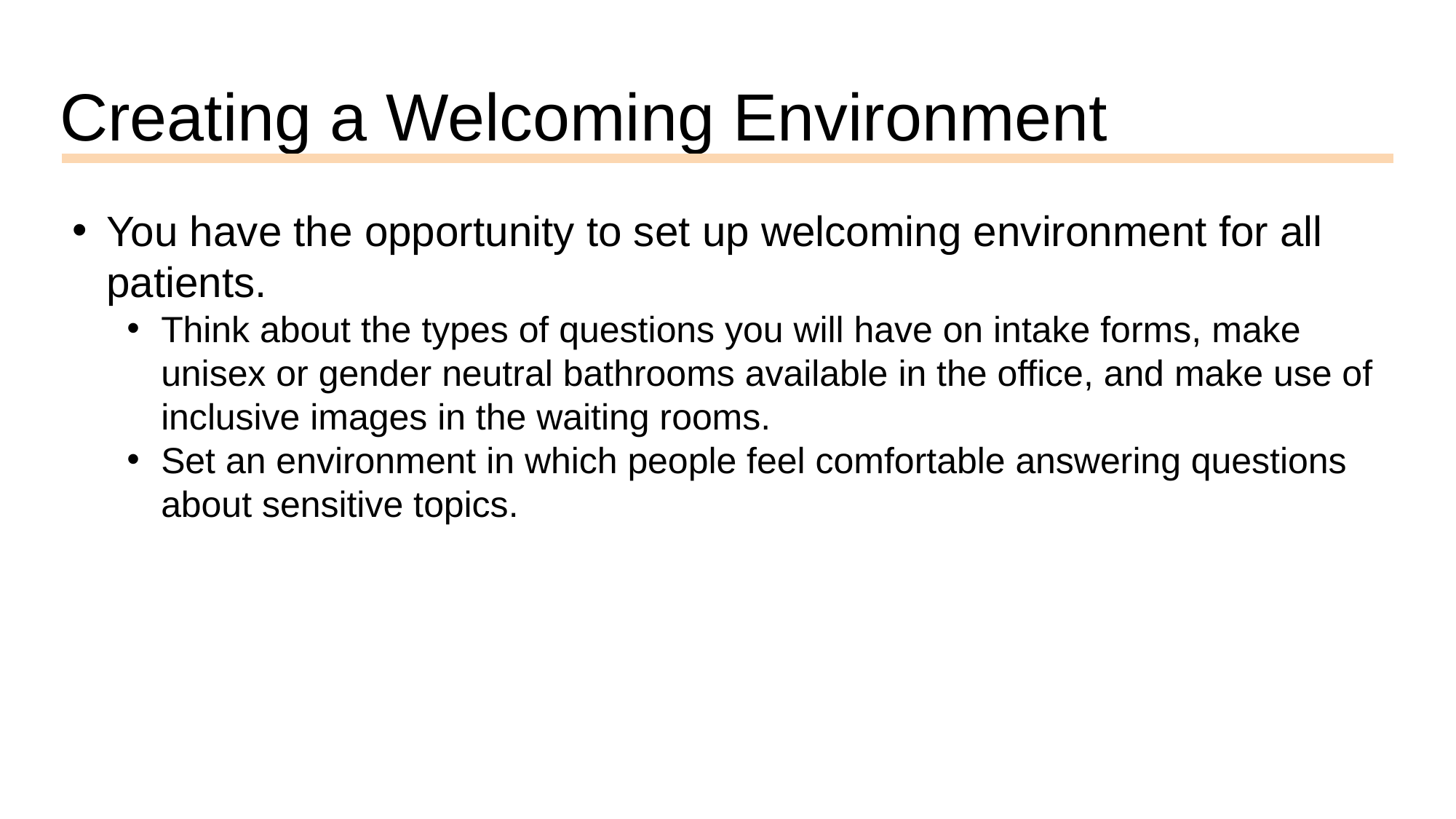

# Creating a Welcoming Environment
You have the opportunity to set up welcoming environment for all patients.
Think about the types of questions you will have on intake forms, make unisex or gender neutral bathrooms available in the office, and make use of inclusive images in the waiting rooms.
Set an environment in which people feel comfortable answering questions about sensitive topics.

## Slide 36
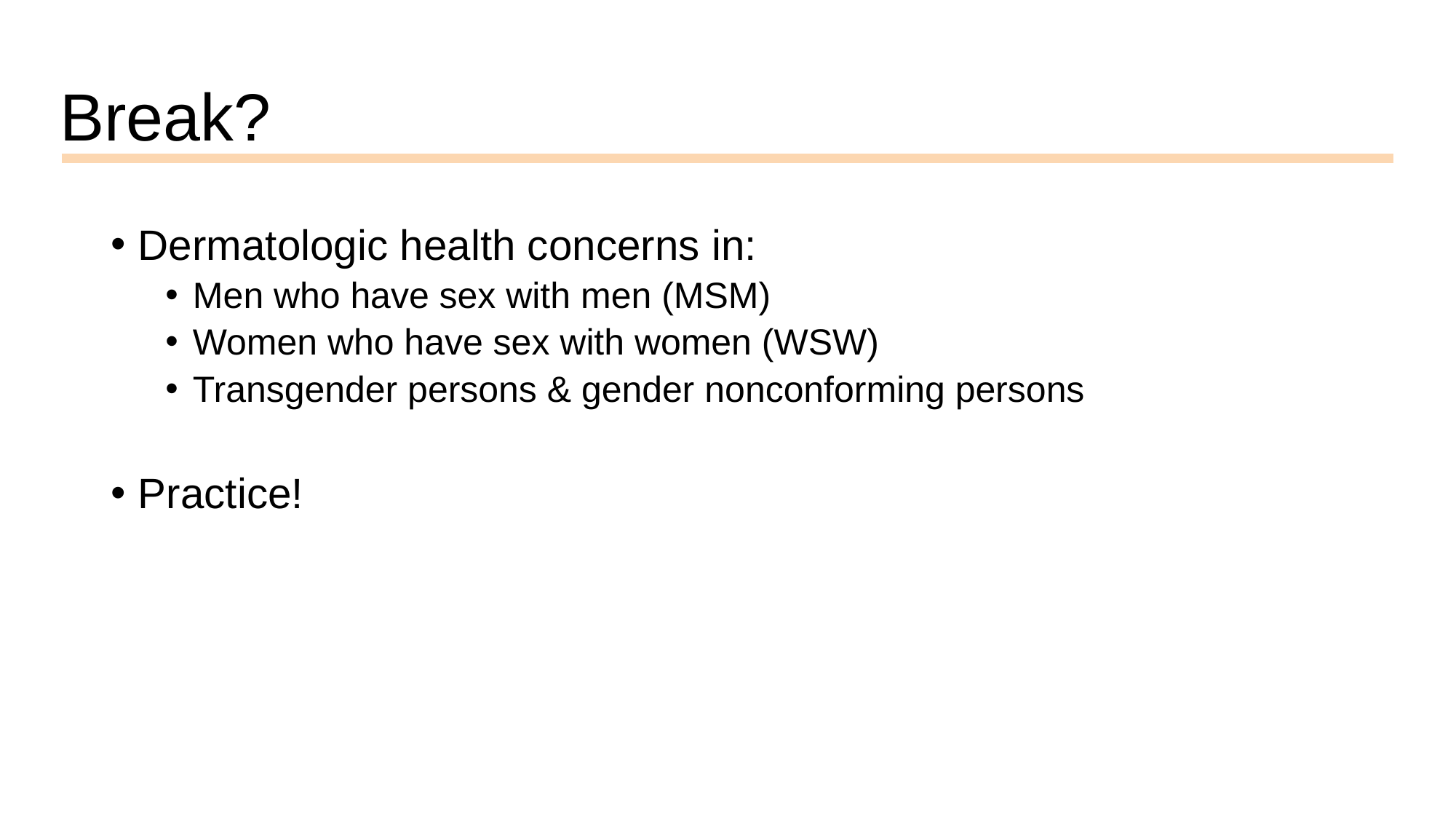

# Break?
Dermatologic health concerns in:
Men who have sex with men (MSM)
Women who have sex with women (WSW)
Transgender persons & gender nonconforming persons
Practice!

## Slide 37
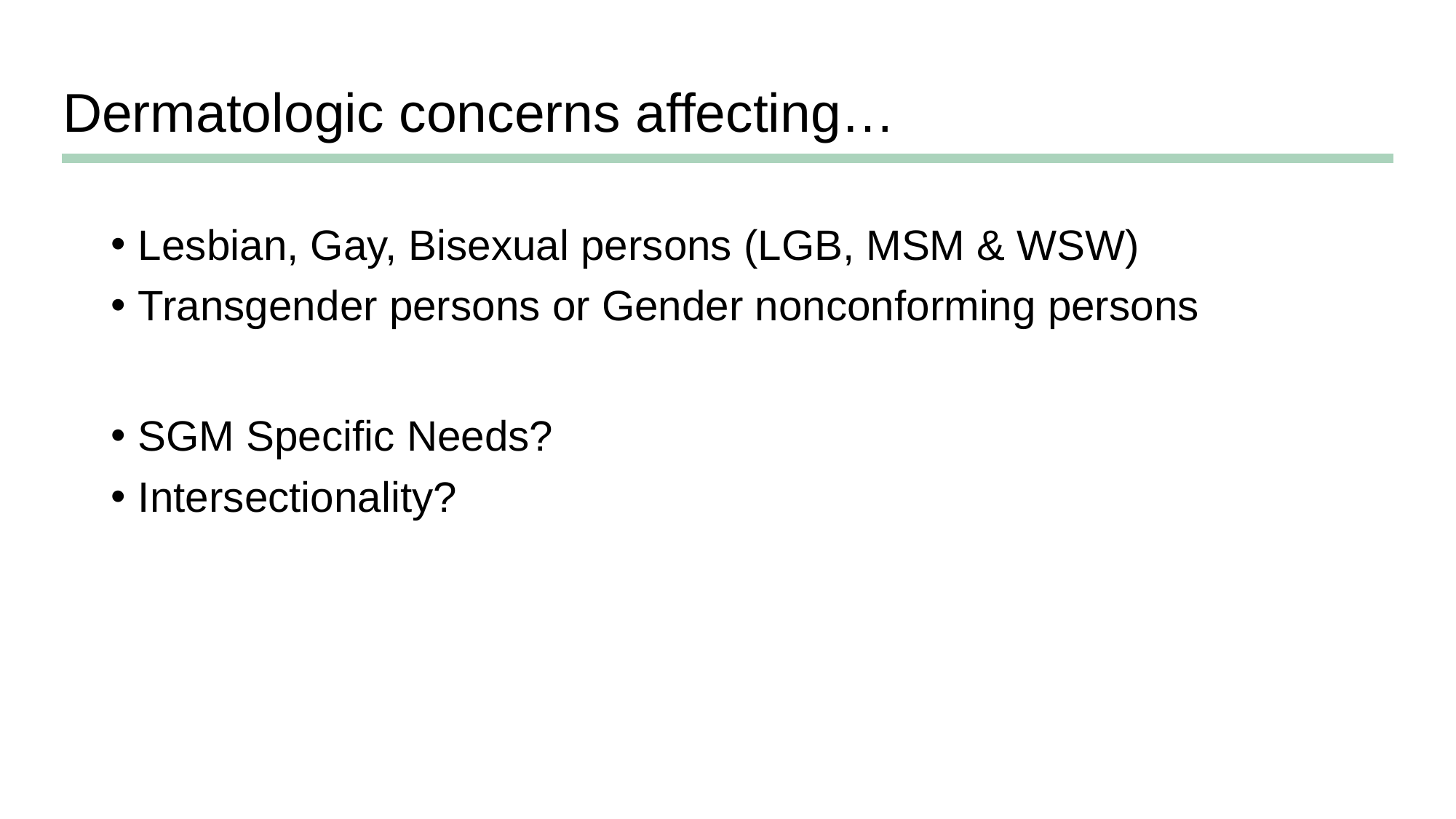

Dermatologic concerns affecting…
Lesbian, Gay, Bisexual persons (LGB, MSM & WSW)
Transgender persons or Gender nonconforming persons
SGM Specific Needs?
Intersectionality?

## Slide 38
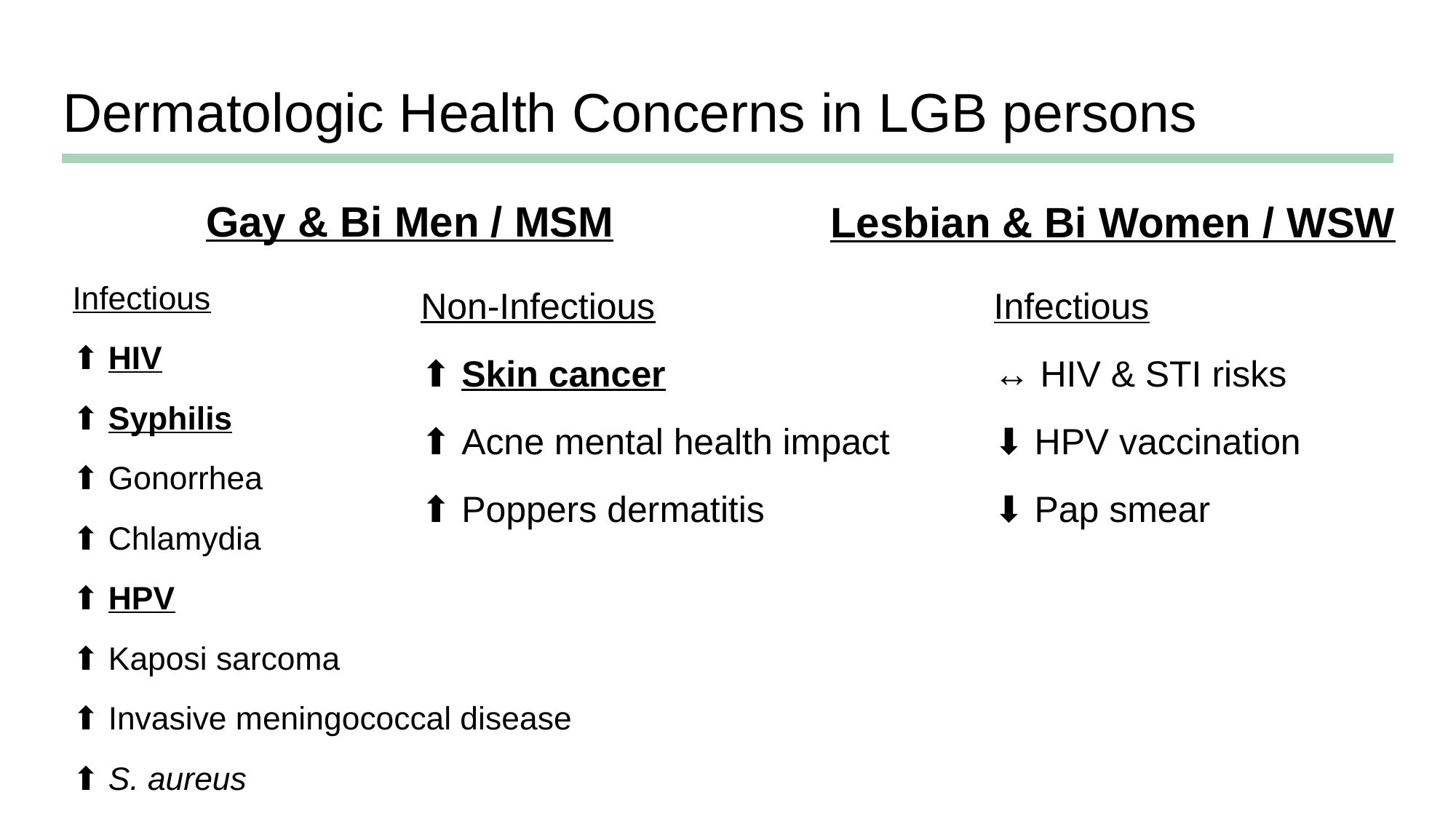

Dermatologic Health Concerns in LGB persons
Gay & Bi Men / MSM
Lesbian & Bi Women / WSW
Infectious
⬆️ HIV
⬆️ Syphilis
⬆️ Gonorrhea
⬆️ Chlamydia
⬆️ HPV
⬆️ Kaposi sarcoma
⬆️ Invasive meningococcal disease
⬆️ S. aureus
Non-Infectious
⬆️ Skin cancer
⬆️ Acne mental health impact
⬆️ Poppers dermatitis
Infectious
↔️ HIV & STI risks
⬇️ HPV vaccination
⬇️ Pap smear

## Slide 39
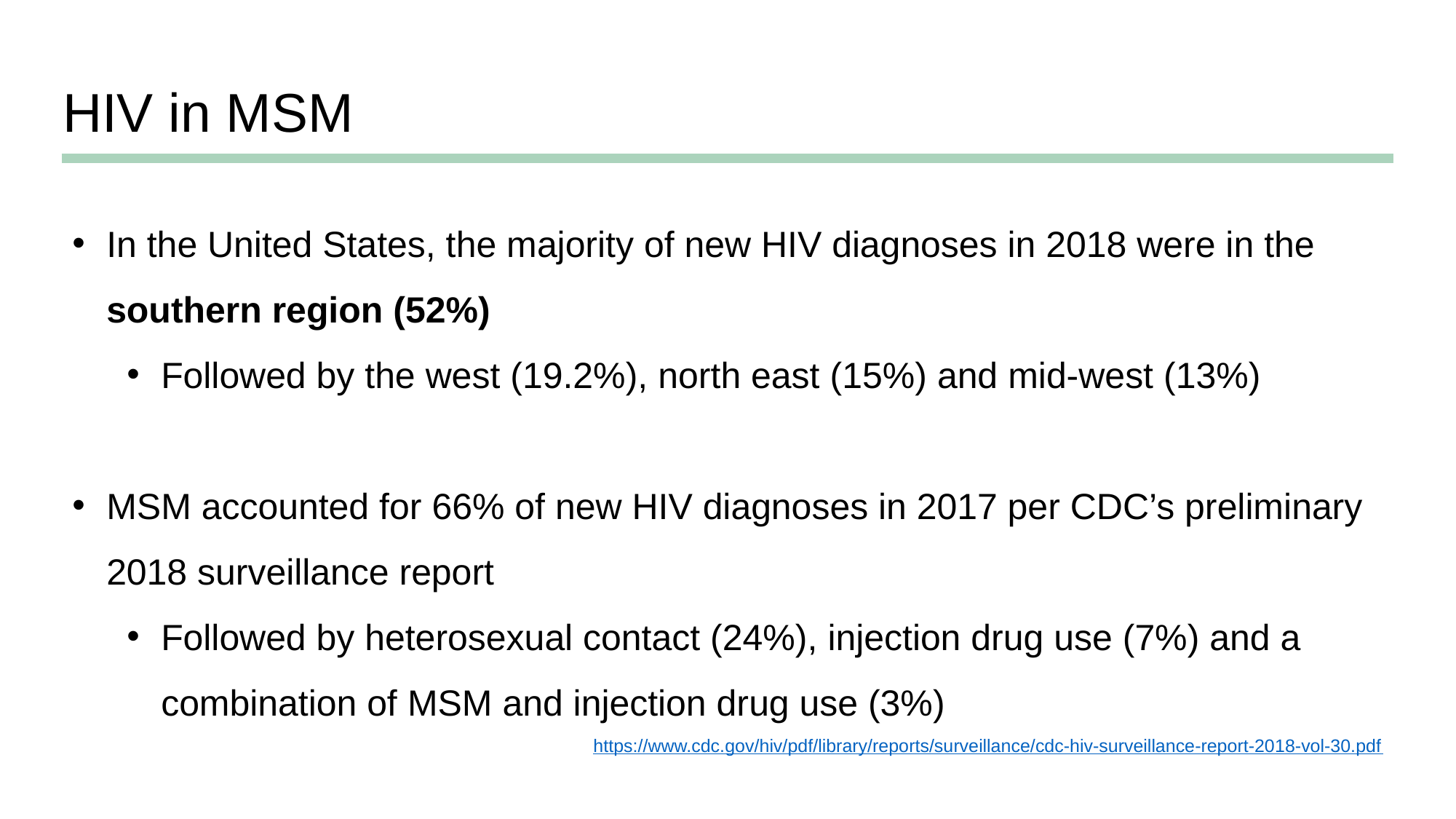

HIV in MSM
In the United States, the majority of new HIV diagnoses in 2018 were in the southern region (52%)
Followed by the west (19.2%), north east (15%) and mid-west (13%)
MSM accounted for 66% of new HIV diagnoses in 2017 per CDC’s preliminary 2018 surveillance report
Followed by heterosexual contact (24%), injection drug use (7%) and a combination of MSM and injection drug use (3%)
https://www.cdc.gov/hiv/pdf/library/reports/surveillance/cdc-hiv-surveillance-report-2018-vol-30.pdf

## Slide 40
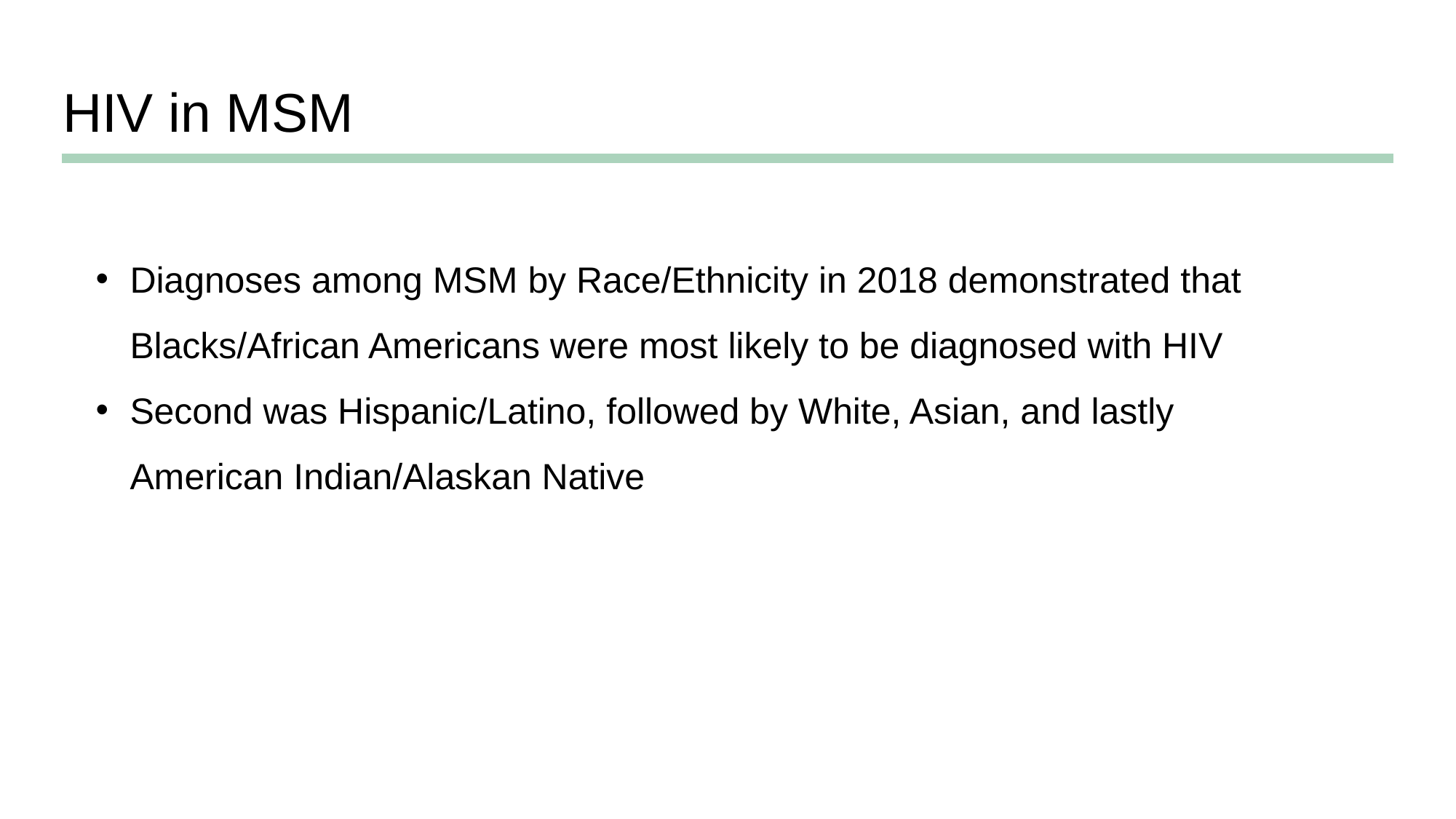

HIV in MSM
Diagnoses among MSM by Race/Ethnicity in 2018 demonstrated that Blacks/African Americans were most likely to be diagnosed with HIV
Second was Hispanic/Latino, followed by White, Asian, and lastly American Indian/Alaskan Native

## Slide 41
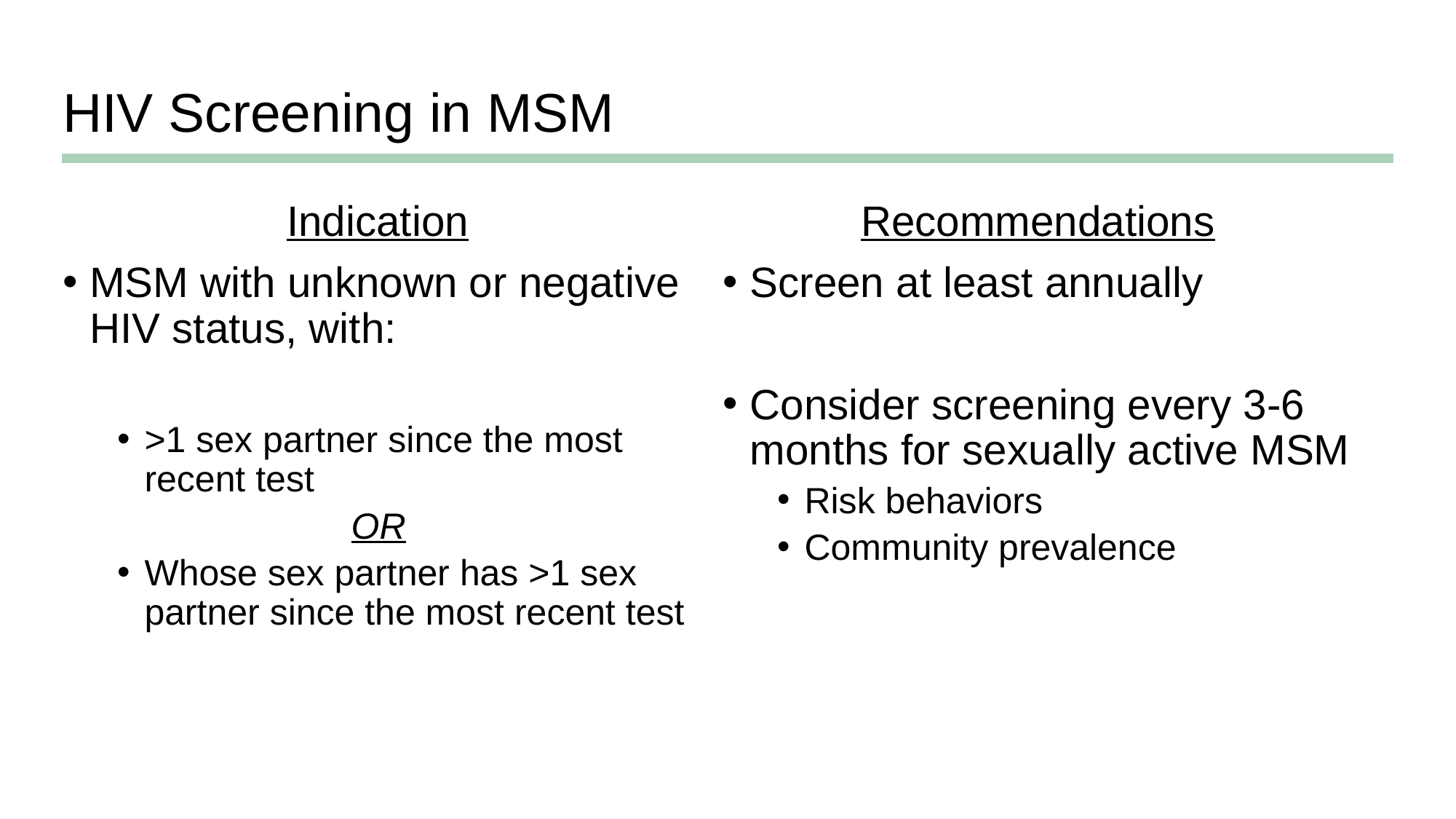

HIV Screening in MSM
Indication
MSM with unknown or negative HIV status, with:
>1 sex partner since the most recent test
OR
Whose sex partner has >1 sex partner since the most recent test
Recommendations
Screen at least annually
Consider screening every 3-6 months for sexually active MSM
Risk behaviors
Community prevalence

## Slide 42
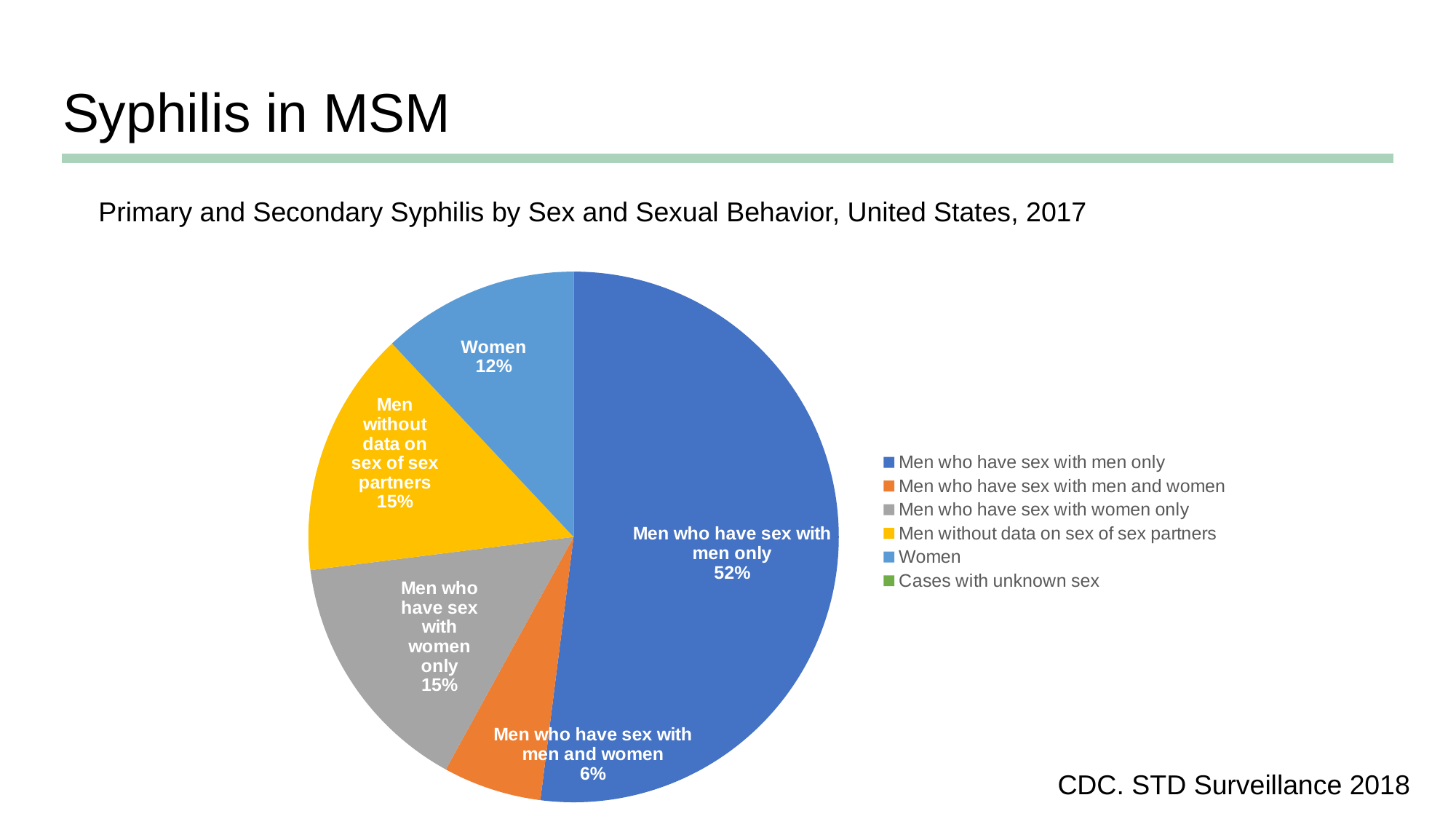

Syphilis in MSM
Primary and Secondary Syphilis by Sex and Sexual Behavior, United States, 2017
### Chart
| Category | Sales |
|---|---|
| Men who have sex with men only | 0.52 |
| Men who have sex with men and women | 0.06 |
| Men who have sex with women only | 0.15 |
| Men without data on sex of sex partners | 0.15 |
| Women | 0.12 |
| Cases with unknown sex | 0.0 |CDC. STD Surveillance 2018

## Slide 43
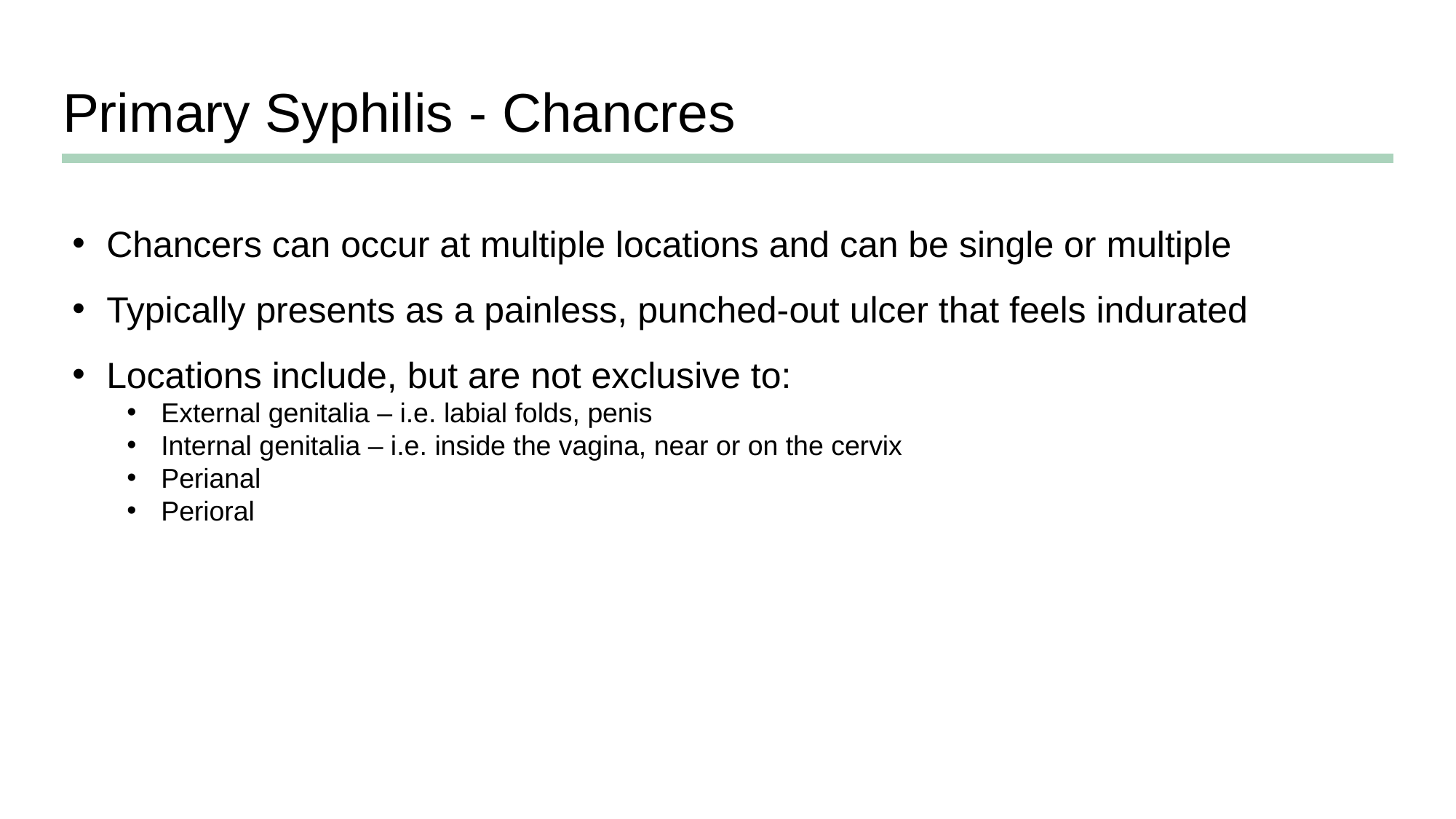

Primary Syphilis - Chancres
Chancers can occur at multiple locations and can be single or multiple
Typically presents as a painless, punched-out ulcer that feels indurated
Locations include, but are not exclusive to:
External genitalia – i.e. labial folds, penis
Internal genitalia – i.e. inside the vagina, near or on the cervix
Perianal
Perioral

## Slide 44
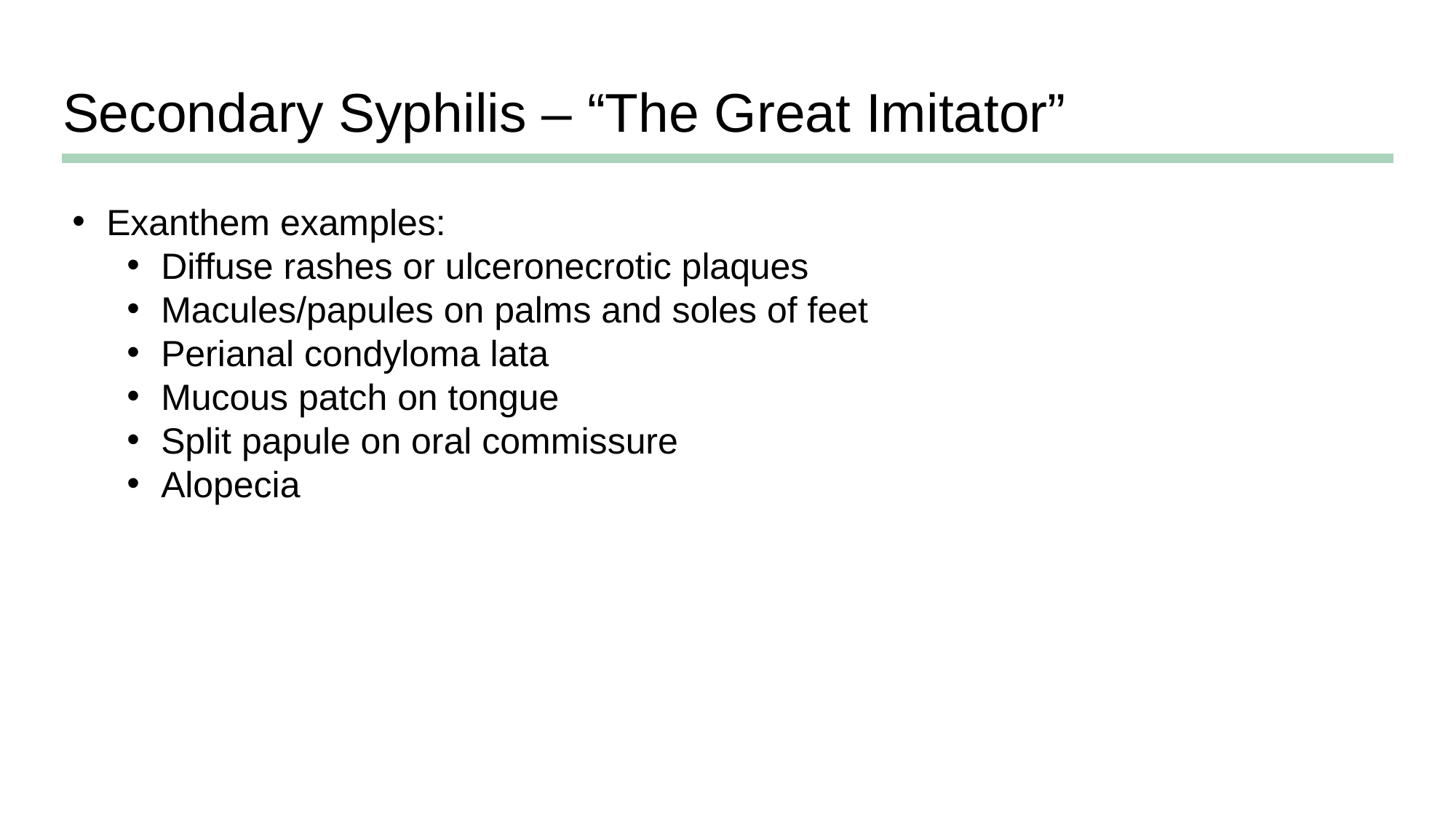

Secondary Syphilis – “The Great Imitator”
Exanthem examples:
Diffuse rashes or ulceronecrotic plaques
Macules/papules on palms and soles of feet
Perianal condyloma lata
Mucous patch on tongue
Split papule on oral commissure
Alopecia

## Slide 45
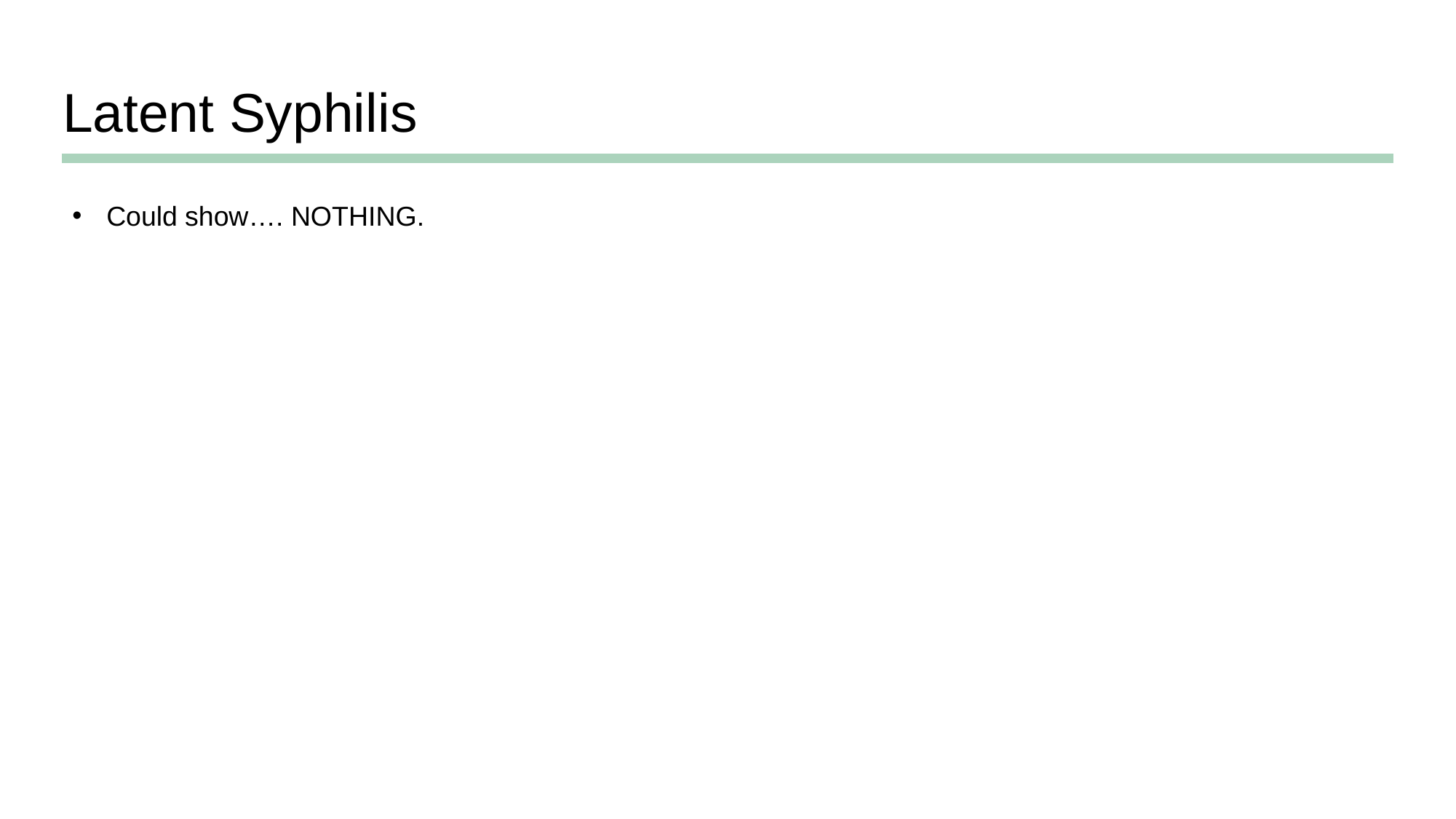

Latent Syphilis
Could show…. NOTHING.

## Slide 46
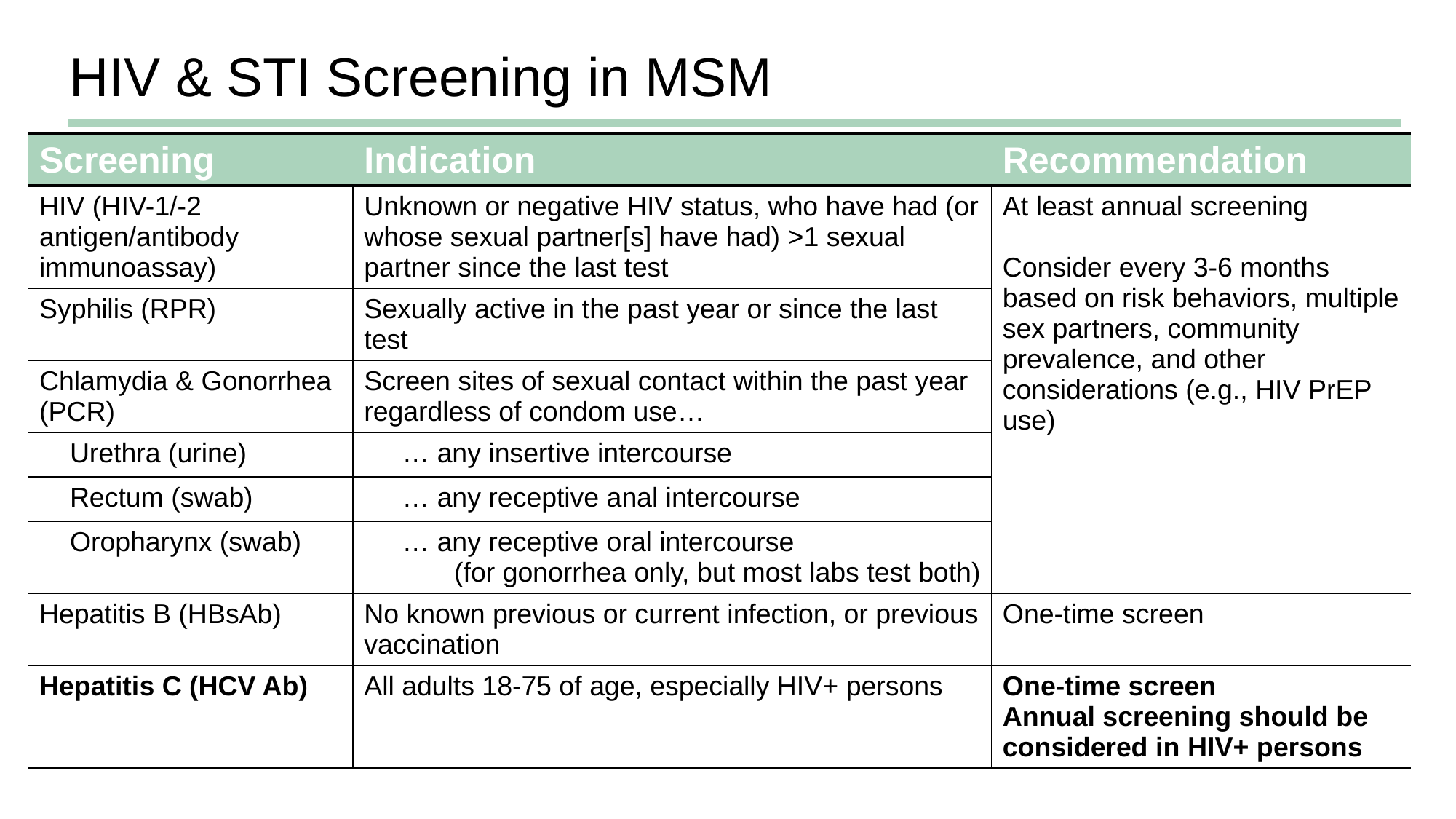

HIV & STI Screening in MSM
| Screening | Indication | Recommendation |
| --- | --- | --- |
| HIV (HIV-1/-2 antigen/antibody immunoassay) | Unknown or negative HIV status, who have had (or whose sexual partner[s] have had) >1 sexual partner since the last test | At least annual screening Consider every 3-6 months based on risk behaviors, multiple sex partners, community prevalence, and other considerations (e.g., HIV PrEP use) |
| Syphilis (RPR) | Sexually active in the past year or since the last test | |
| Chlamydia & Gonorrhea (PCR) | Screen sites of sexual contact within the past year regardless of condom use… | |
| Urethra (urine) | … any insertive intercourse | |
| Rectum (swab) | … any receptive anal intercourse | |
| Oropharynx (swab) | … any receptive oral intercourse (for gonorrhea only, but most labs test both) | |
| Hepatitis B (HBsAb) | No known previous or current infection, or previous vaccination | One-time screen |
| Hepatitis C (HCV Ab) | All adults 18-75 of age, especially HIV+ persons | One-time screen Annual screening should be considered in HIV+ persons |

## Slide 47
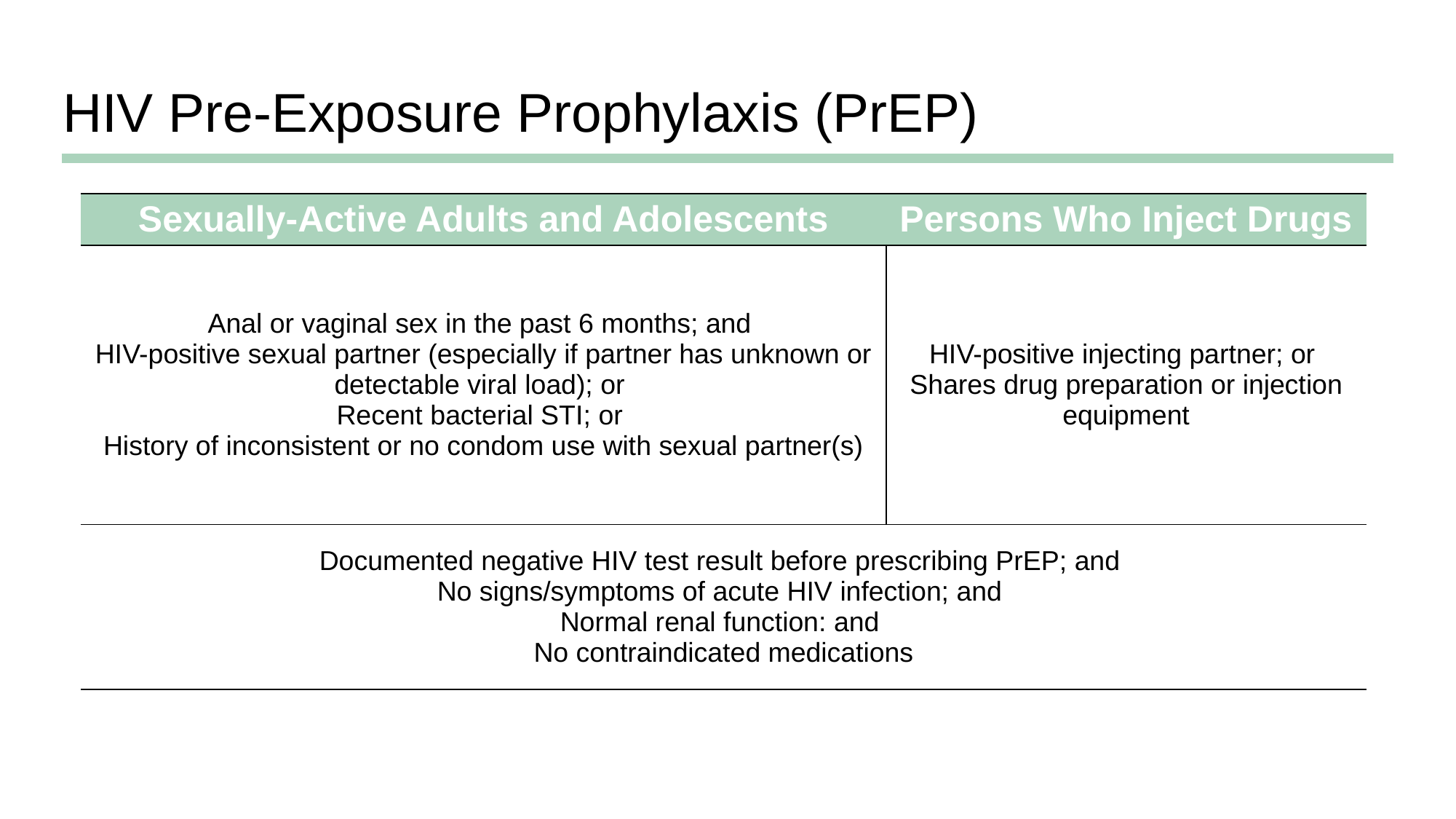

HIV Pre-Exposure Prophylaxis (PrEP)
| Sexually-Active Adults and Adolescents | Persons Who Inject Drugs |
| --- | --- |
| Anal or vaginal sex in the past 6 months; and HIV-positive sexual partner (especially if partner has unknown or detectable viral load); or Recent bacterial STI; or History of inconsistent or no condom use with sexual partner(s) | HIV-positive injecting partner; or Shares drug preparation or injection equipment |
| Documented negative HIV test result before prescribing PrEP; and No signs/symptoms of acute HIV infection; and Normal renal function: and No contraindicated medications | |

## Slide 48
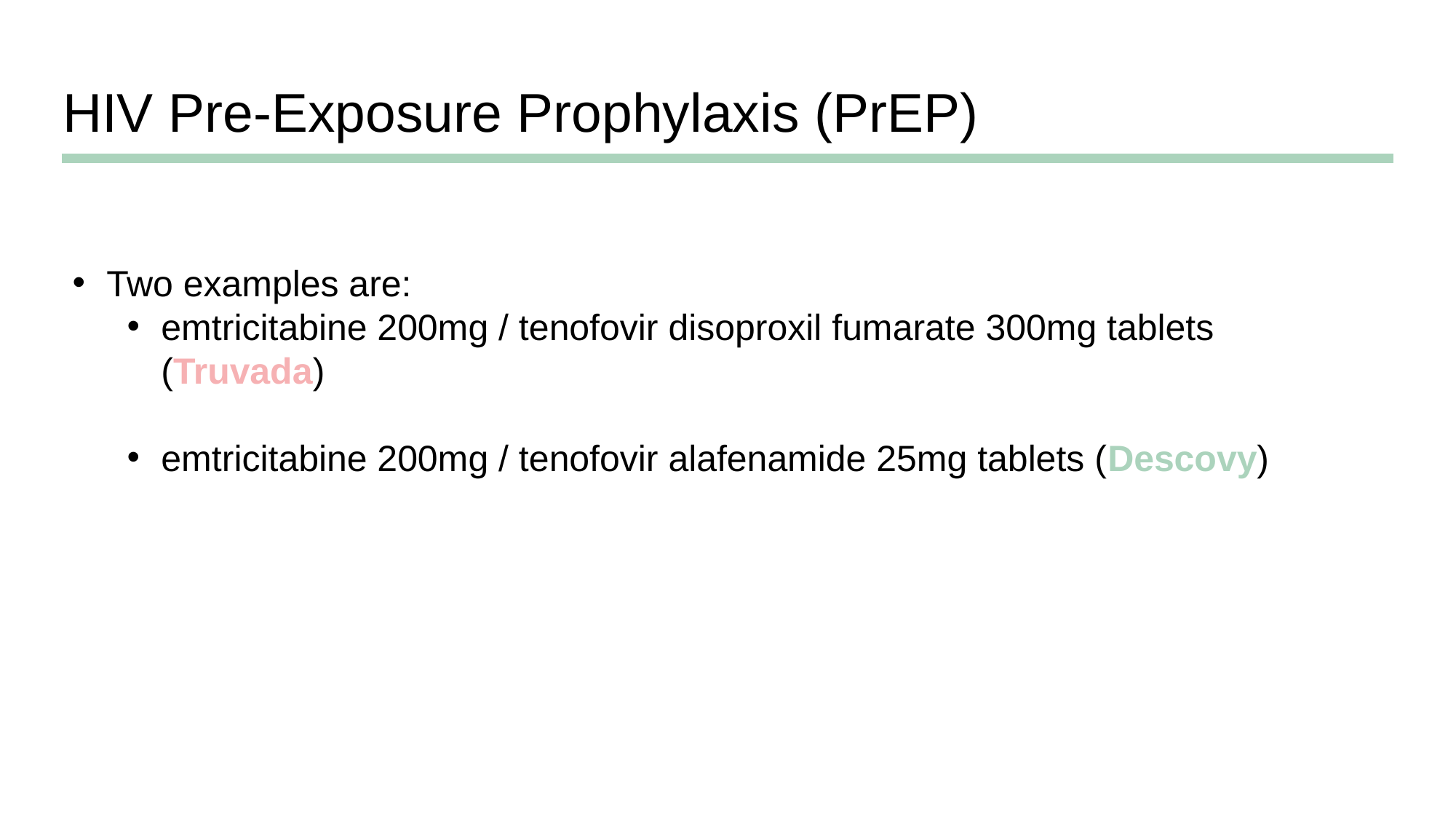

HIV Pre-Exposure Prophylaxis (PrEP)
Two examples are:
emtricitabine 200mg / tenofovir disoproxil fumarate 300mg tablets (Truvada)
emtricitabine 200mg / tenofovir alafenamide 25mg tablets (Descovy)

## Slide 49
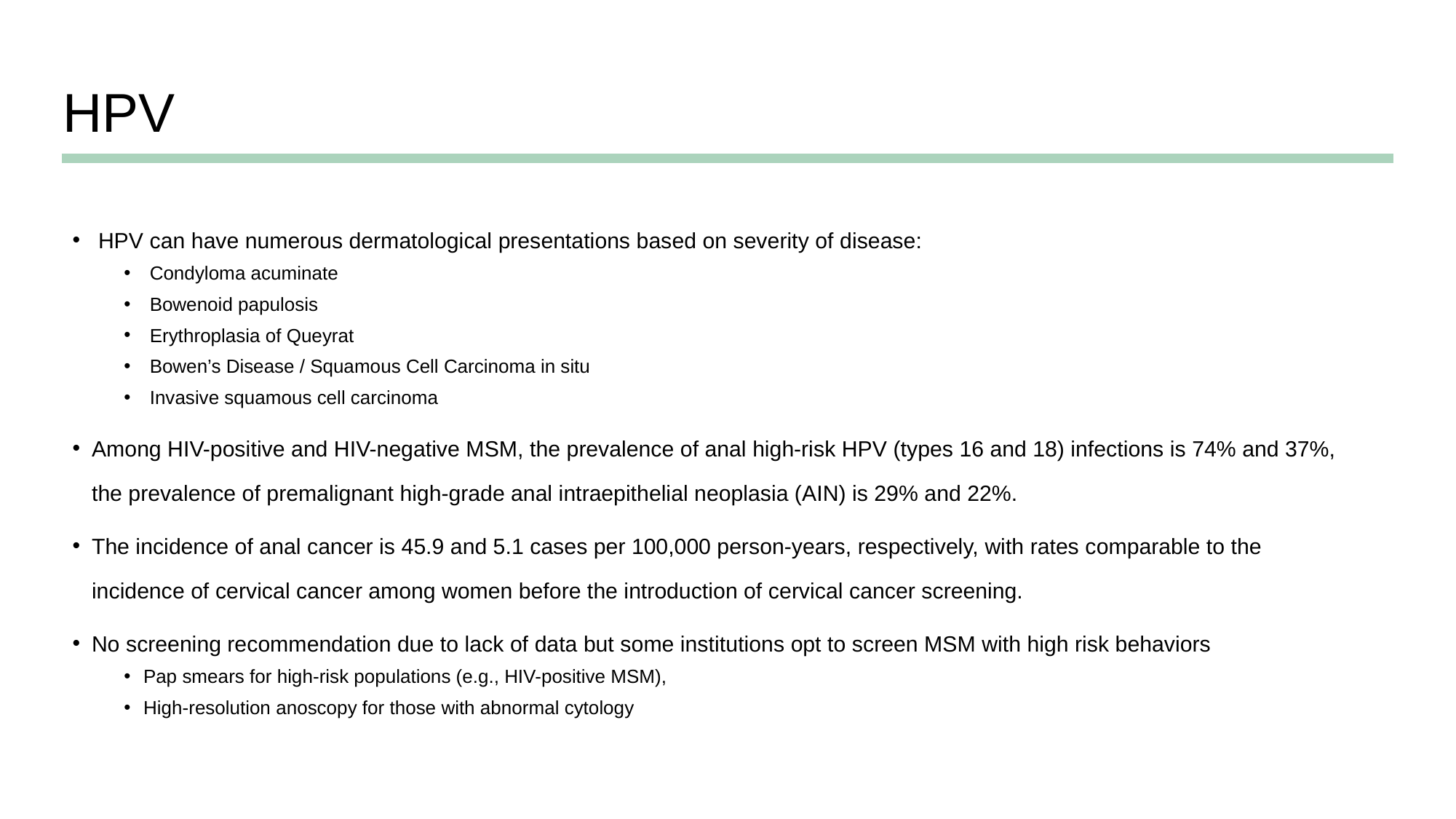

HPV
HPV can have numerous dermatological presentations based on severity of disease:
Condyloma acuminate
Bowenoid papulosis
Erythroplasia of Queyrat
Bowen’s Disease / Squamous Cell Carcinoma in situ
Invasive squamous cell carcinoma
Among HIV-positive and HIV-negative MSM, the prevalence of anal high-risk HPV (types 16 and 18) infections is 74% and 37%, the prevalence of premalignant high-grade anal intraepithelial neoplasia (AIN) is 29% and 22%.
The incidence of anal cancer is 45.9 and 5.1 cases per 100,000 person-years, respectively, with rates comparable to the incidence of cervical cancer among women before the introduction of cervical cancer screening.
No screening recommendation due to lack of data but some institutions opt to screen MSM with high risk behaviors
Pap smears for high-risk populations (e.g., HIV-positive MSM),
High-resolution anoscopy for those with abnormal cytology

## Slide 50
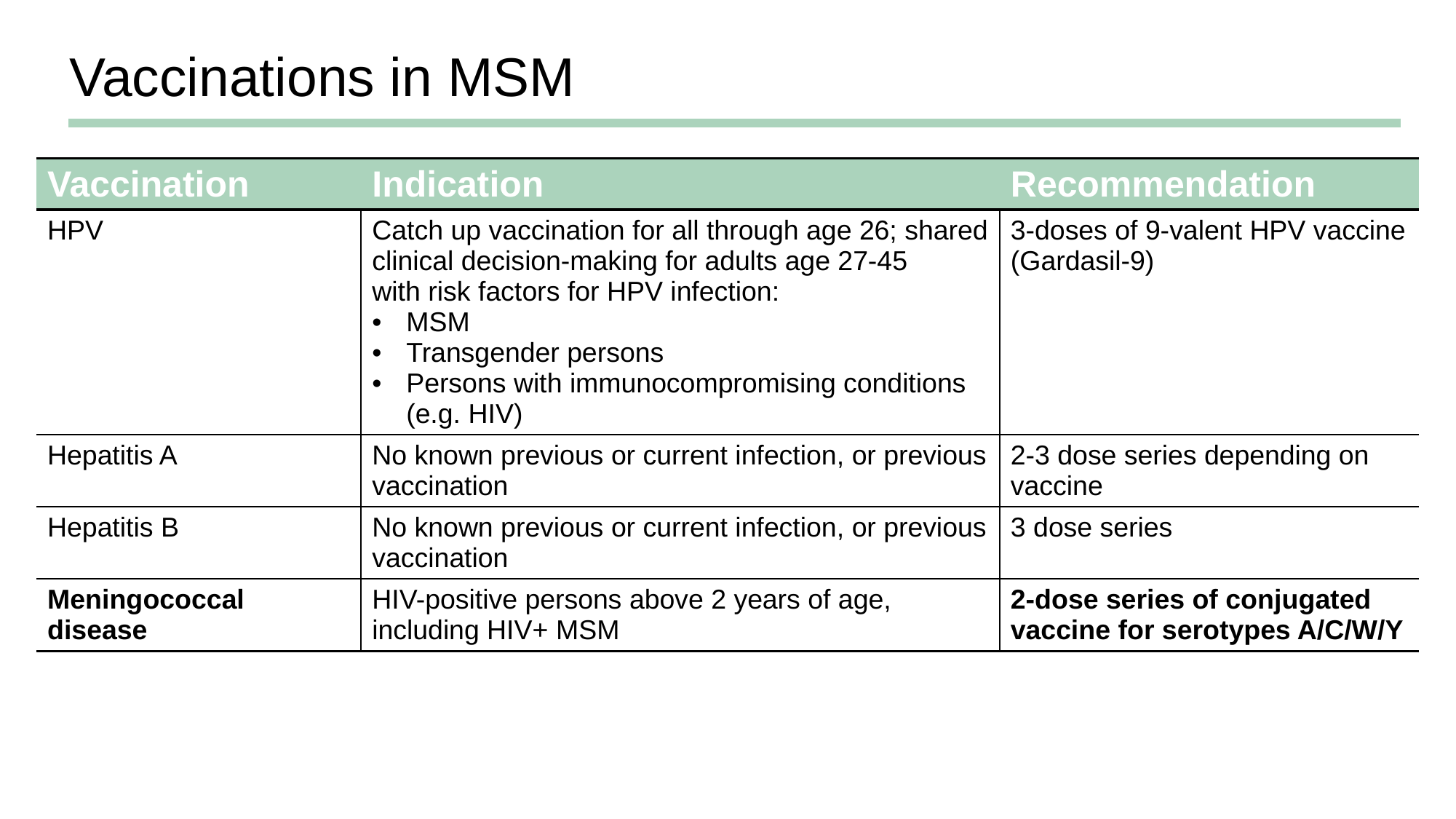

Vaccinations in MSM
| Vaccination | Indication | Recommendation |
| --- | --- | --- |
| HPV | Catch up vaccination for all through age 26; shared clinical decision-making for adults age 27-45 with risk factors for HPV infection: MSM Transgender persons Persons with immunocompromising conditions (e.g. HIV) | 3-doses of 9-valent HPV vaccine (Gardasil-9) |
| Hepatitis A | No known previous or current infection, or previous vaccination | 2-3 dose series depending on vaccine |
| Hepatitis B | No known previous or current infection, or previous vaccination | 3 dose series |
| Meningococcal disease | HIV-positive persons above 2 years of age, including HIV+ MSM | 2-dose series of conjugated vaccine for serotypes A/C/W/Y |

## Slide 51
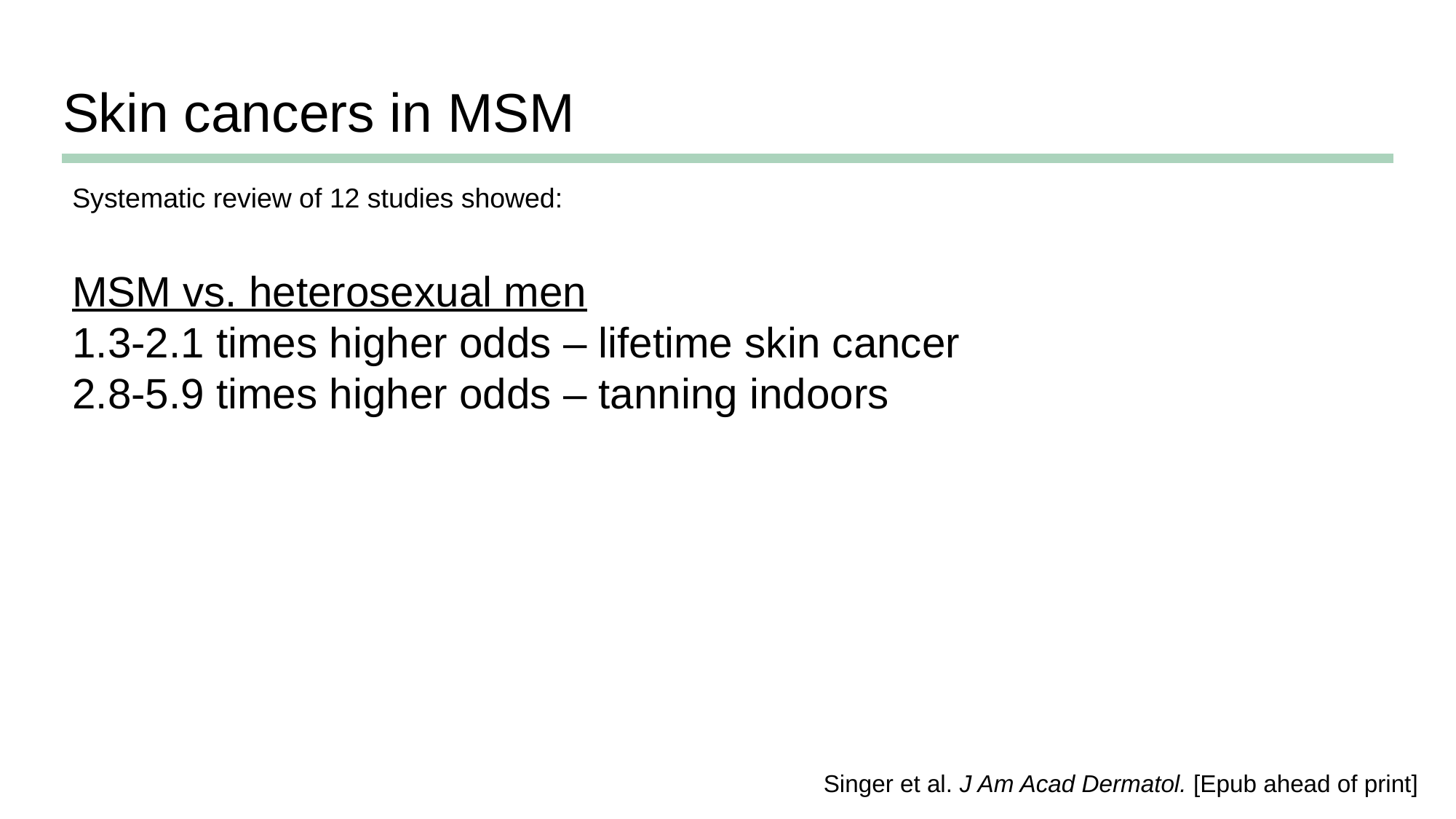

Skin cancers in MSM
Systematic review of 12 studies showed:
MSM vs. heterosexual men
1.3-2.1 times higher odds – lifetime skin cancer
2.8-5.9 times higher odds – tanning indoors
Singer et al. J Am Acad Dermatol. [Epub ahead of print]

## Slide 52
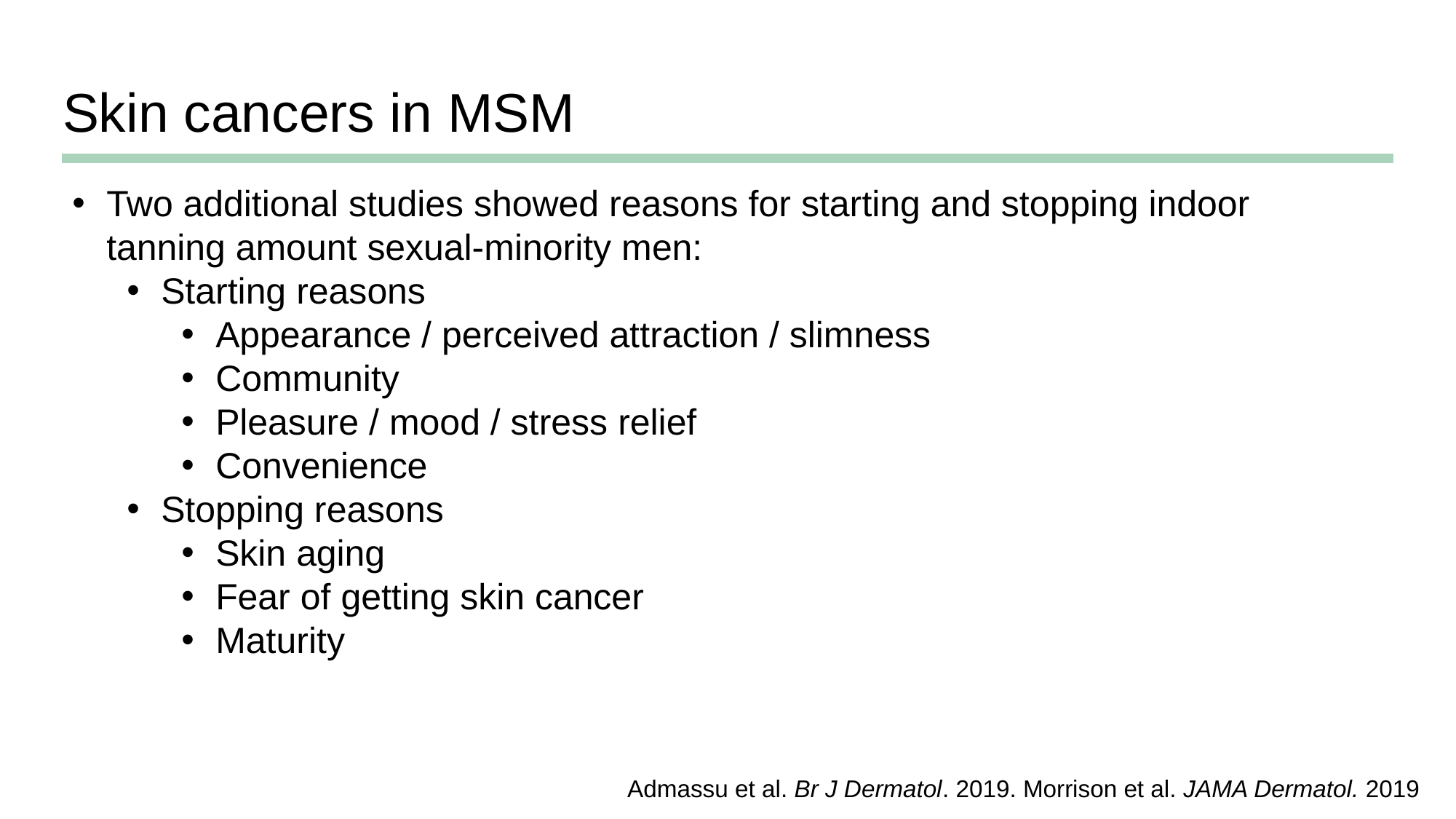

Skin cancers in MSM
Two additional studies showed reasons for starting and stopping indoor tanning amount sexual-minority men:
Starting reasons
Appearance / perceived attraction / slimness
Community
Pleasure / mood / stress relief
Convenience
Stopping reasons
Skin aging
Fear of getting skin cancer
Maturity
Admassu et al. Br J Dermatol. 2019. Morrison et al. JAMA Dermatol. 2019

## Slide 53
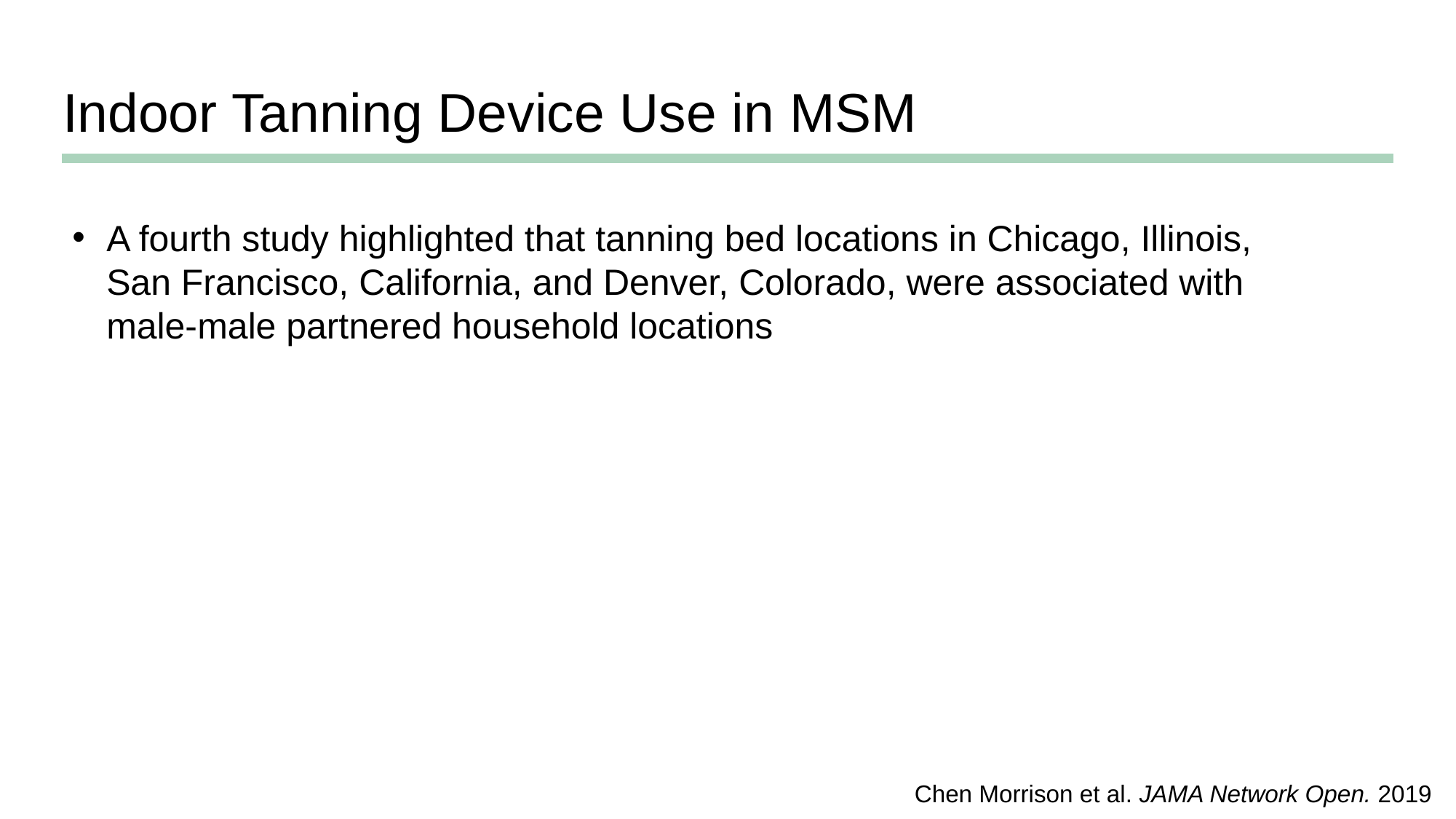

Indoor Tanning Device Use in MSM
A fourth study highlighted that tanning bed locations in Chicago, Illinois, San Francisco, California, and Denver, Colorado, were associated with male-male partnered household locations
Chen Morrison et al. JAMA Network Open. 2019

## Slide 54
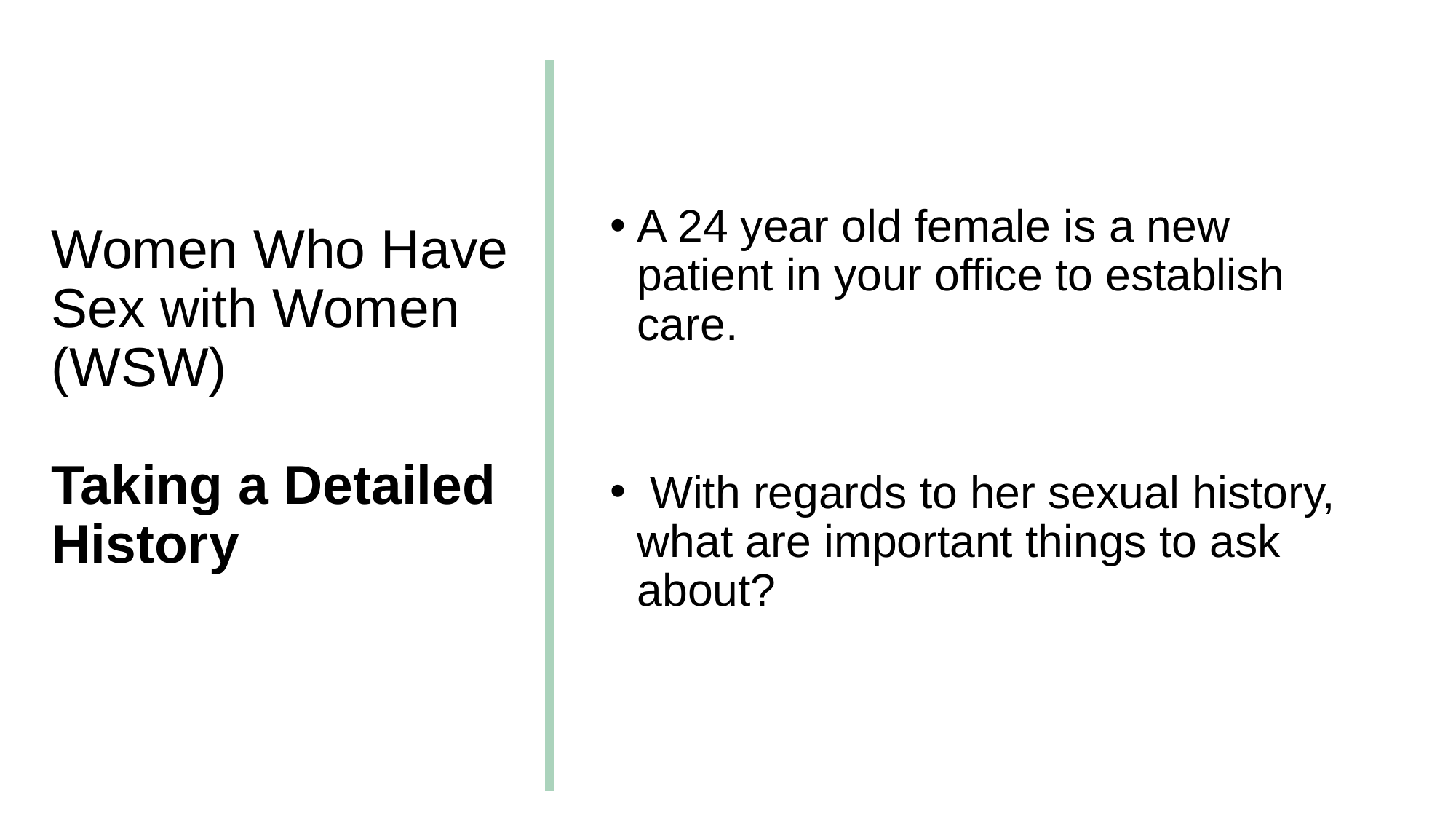

# Women Who Have Sex with Women (WSW) Taking a Detailed History
A 24 year old female is a new patient in your office to establish care.
 With regards to her sexual history, what are important things to ask about?

## Slide 55
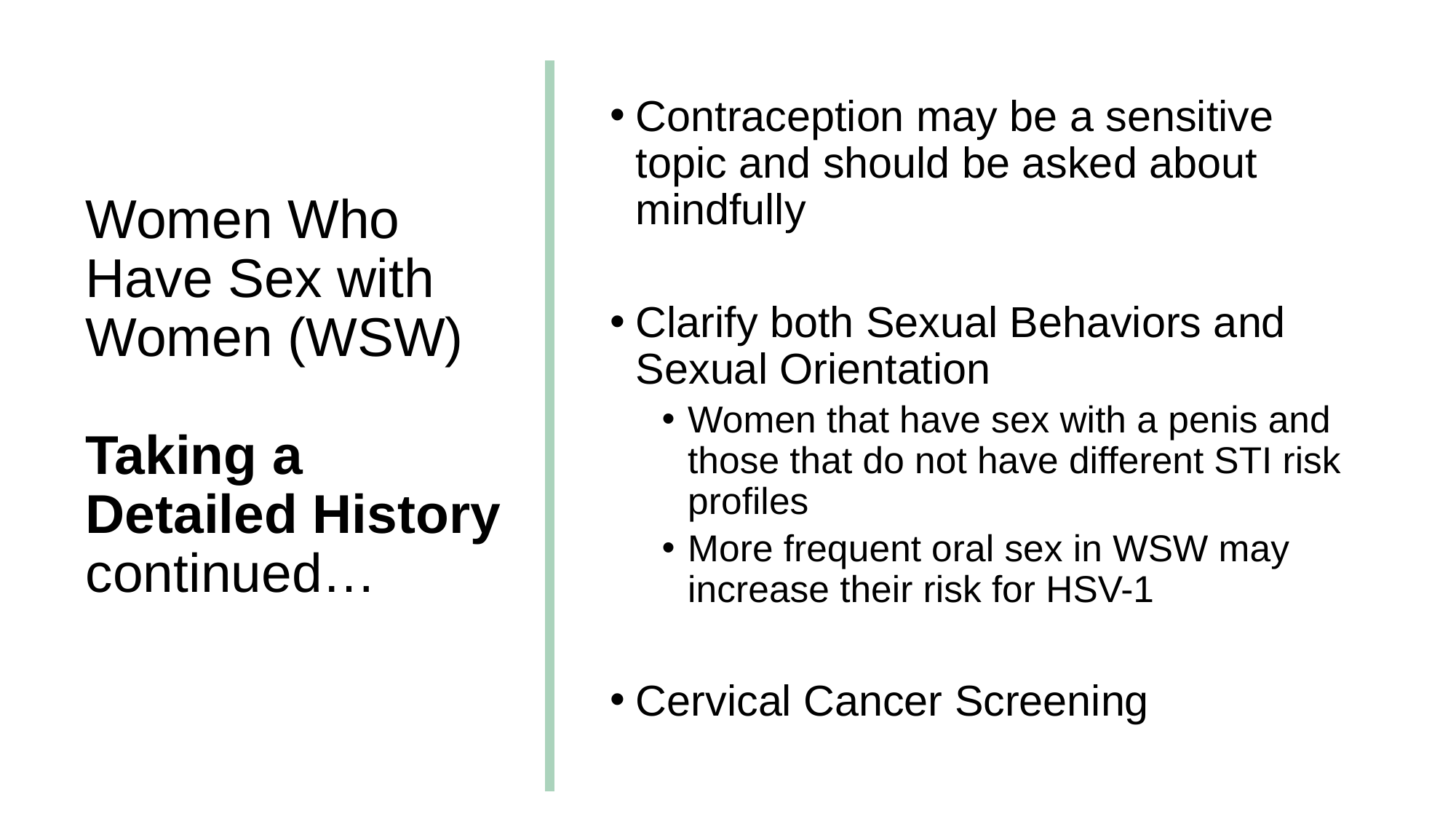

# Women Who Have Sex with Women (WSW) Taking a Detailed History continued…
Contraception may be a sensitive topic and should be asked about mindfully
Clarify both Sexual Behaviors and Sexual Orientation
Women that have sex with a penis and those that do not have different STI risk profiles
More frequent oral sex in WSW may increase their risk for HSV-1
Cervical Cancer Screening

## Slide 56
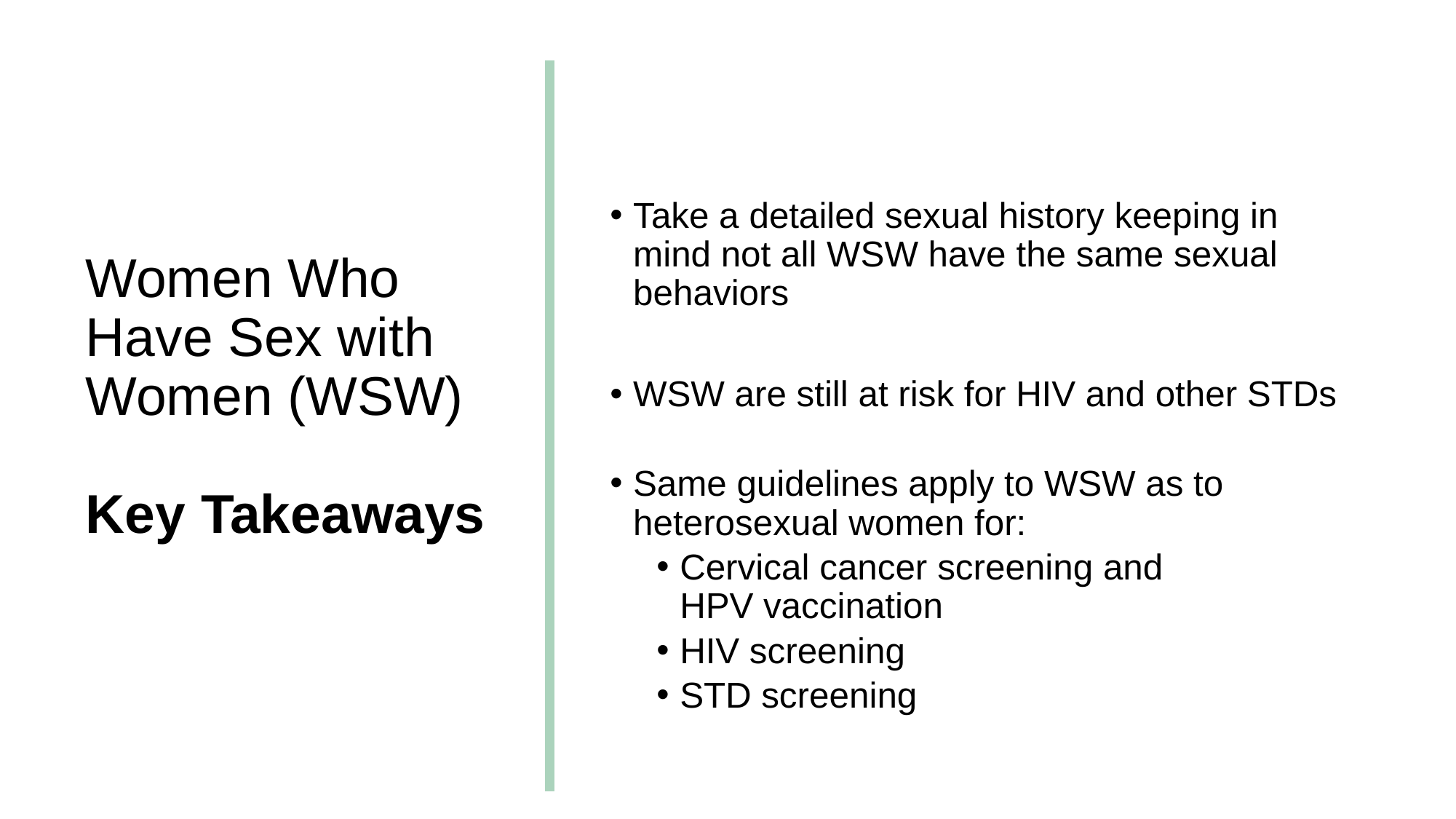

# Women Who Have Sex with Women (WSW) Key Takeaways
Take a detailed sexual history keeping in mind not all WSW have the same sexual behaviors
WSW are still at risk for HIV and other STDs
Same guidelines apply to WSW as to heterosexual women for:
Cervical cancer screening and
HPV vaccination
HIV screening
STD screening

## Slide 57
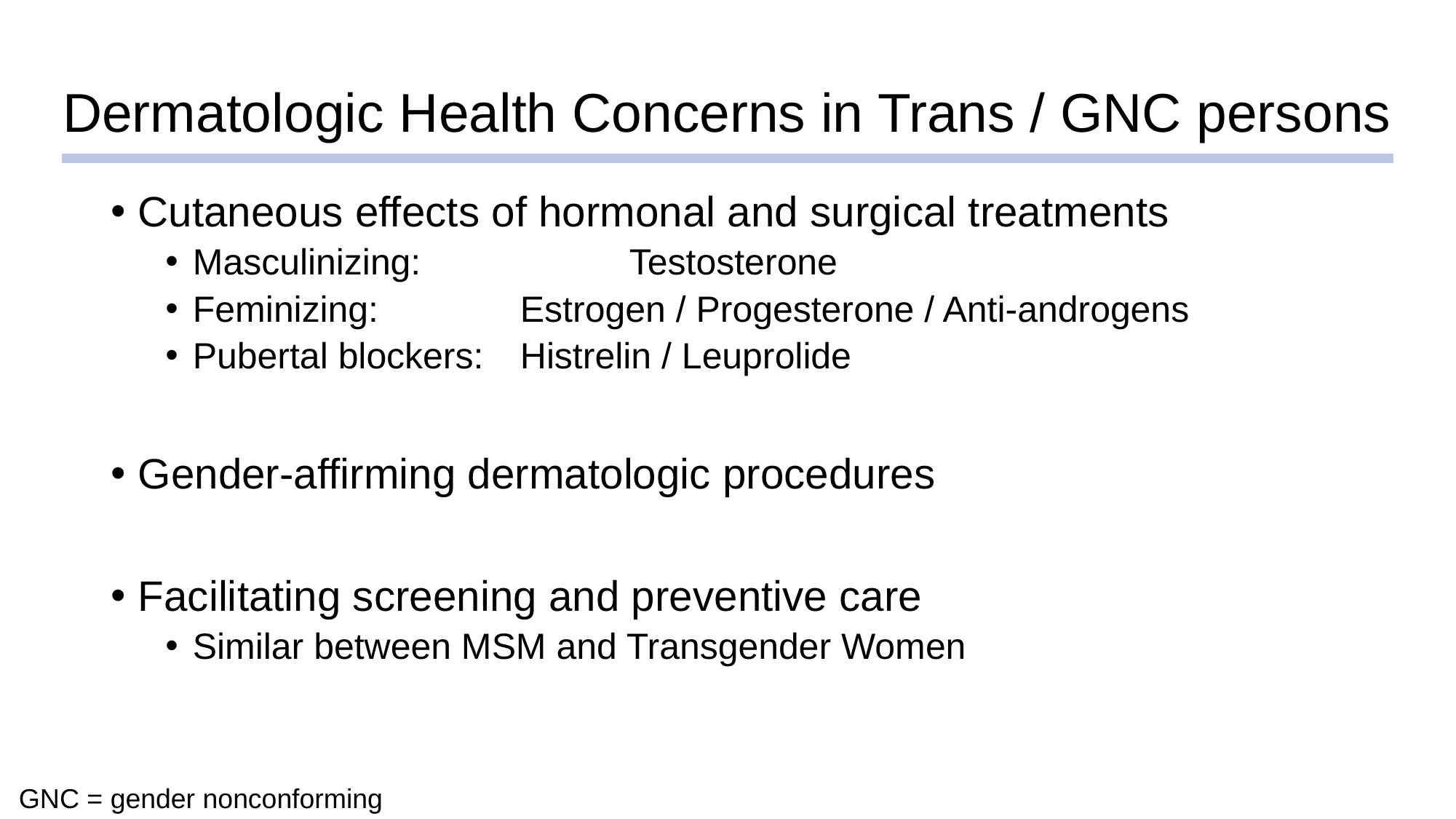

Dermatologic Health Concerns in Trans / GNC persons
Cutaneous effects of hormonal and surgical treatments
Masculinizing: 		Testosterone
Feminizing: 		Estrogen / Progesterone / Anti-androgens
Pubertal blockers: 	Histrelin / Leuprolide
Gender-affirming dermatologic procedures
Facilitating screening and preventive care
Similar between MSM and Transgender Women
GNC = gender nonconforming

## Slide 58
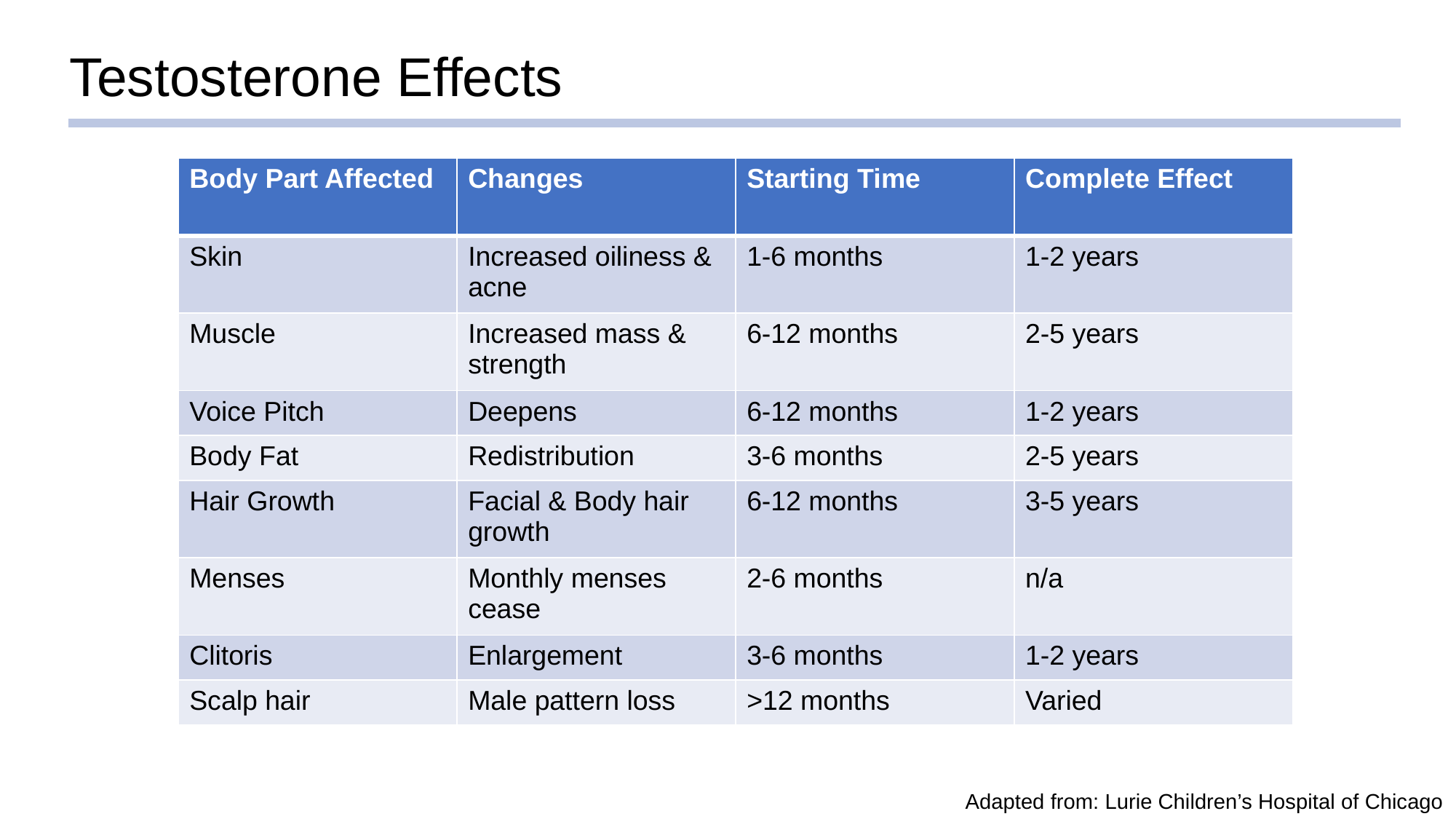

Testosterone Effects
| Body Part Affected | Changes | Starting Time | Complete Effect |
| --- | --- | --- | --- |
| Skin | Increased oiliness & acne | 1-6 months | 1-2 years |
| Muscle | Increased mass & strength | 6-12 months | 2-5 years |
| Voice Pitch | Deepens | 6-12 months | 1-2 years |
| Body Fat | Redistribution | 3-6 months | 2-5 years |
| Hair Growth | Facial & Body hair growth | 6-12 months | 3-5 years |
| Menses | Monthly menses cease | 2-6 months | n/a |
| Clitoris | Enlargement | 3-6 months | 1-2 years |
| Scalp hair | Male pattern loss | >12 months | Varied |
Adapted from: Lurie Children’s Hospital of Chicago

## Slide 59
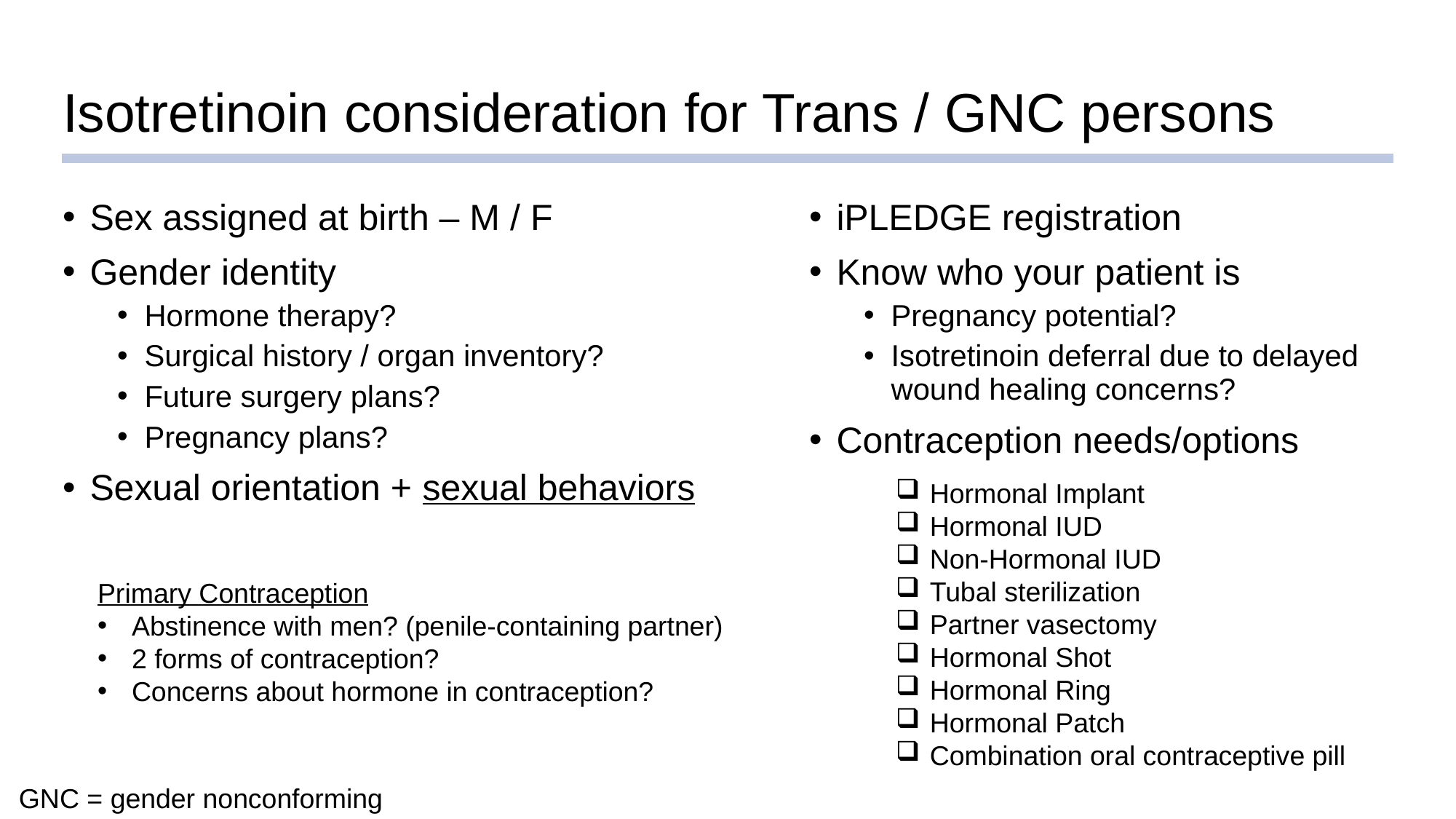

Isotretinoin consideration for Trans / GNC persons
Sex assigned at birth – M / F
Gender identity
Hormone therapy?
Surgical history / organ inventory?
Future surgery plans?
Pregnancy plans?
Sexual orientation + sexual behaviors
iPLEDGE registration
Know who your patient is
Pregnancy potential?
Isotretinoin deferral due to delayed wound healing concerns?
Contraception needs/options
Hormonal Implant
Hormonal IUD
Non-Hormonal IUD
Tubal sterilization
Partner vasectomy
Hormonal Shot
Hormonal Ring
Hormonal Patch
Combination oral contraceptive pill
Primary Contraception
Abstinence with men? (penile-containing partner)
2 forms of contraception?
Concerns about hormone in contraception?
GNC = gender nonconforming

## Slide 60
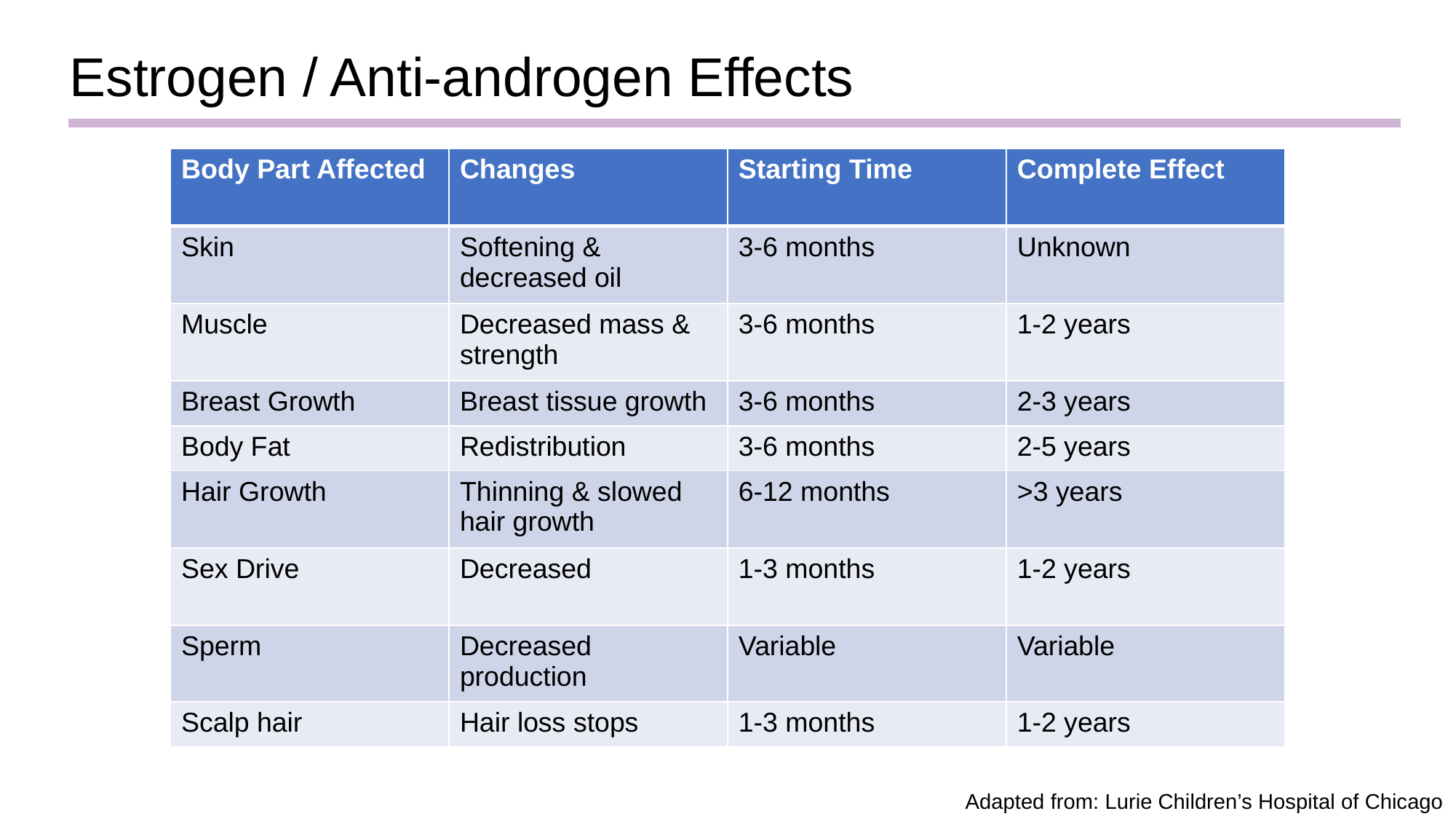

Estrogen / Anti-androgen Effects
| Body Part Affected | Changes | Starting Time | Complete Effect |
| --- | --- | --- | --- |
| Skin | Softening & decreased oil | 3-6 months | Unknown |
| Muscle | Decreased mass & strength | 3-6 months | 1-2 years |
| Breast Growth | Breast tissue growth | 3-6 months | 2-3 years |
| Body Fat | Redistribution | 3-6 months | 2-5 years |
| Hair Growth | Thinning & slowed hair growth | 6-12 months | >3 years |
| Sex Drive | Decreased | 1-3 months | 1-2 years |
| Sperm | Decreased production | Variable | Variable |
| Scalp hair | Hair loss stops | 1-3 months | 1-2 years |
Adapted from: Lurie Children’s Hospital of Chicago

## Slide 61
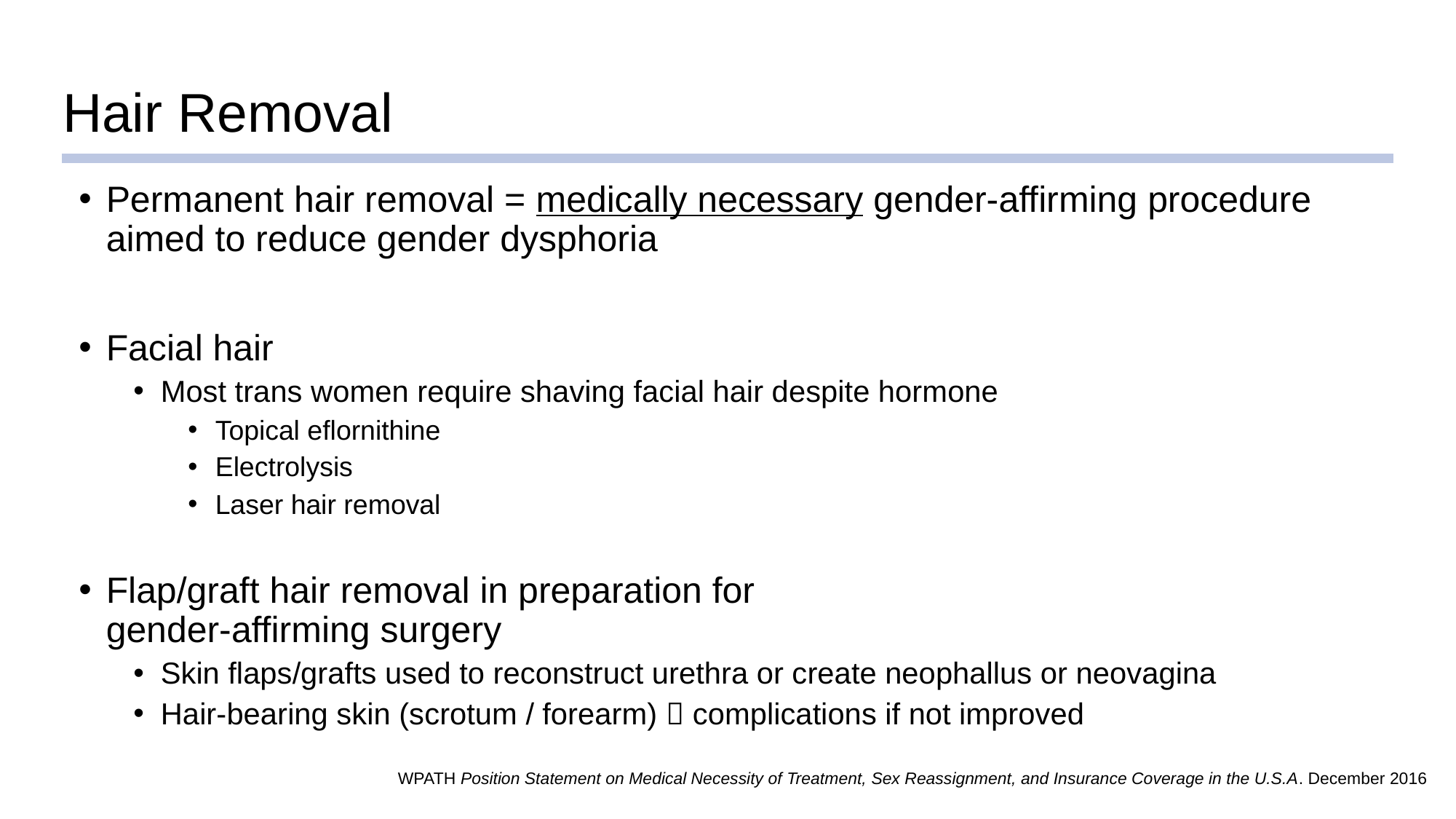

Hair Removal
Permanent hair removal = medically necessary gender-affirming procedure aimed to reduce gender dysphoria
Facial hair
Most trans women require shaving facial hair despite hormone
Topical eflornithine
Electrolysis
Laser hair removal
Flap/graft hair removal in preparation for
gender-affirming surgery
Skin flaps/grafts used to reconstruct urethra or create neophallus or neovagina
Hair-bearing skin (scrotum / forearm)  complications if not improved
WPATH Position Statement on Medical Necessity of Treatment, Sex Reassignment, and Insurance Coverage in the U.S.A. December 2016

## Slide 62
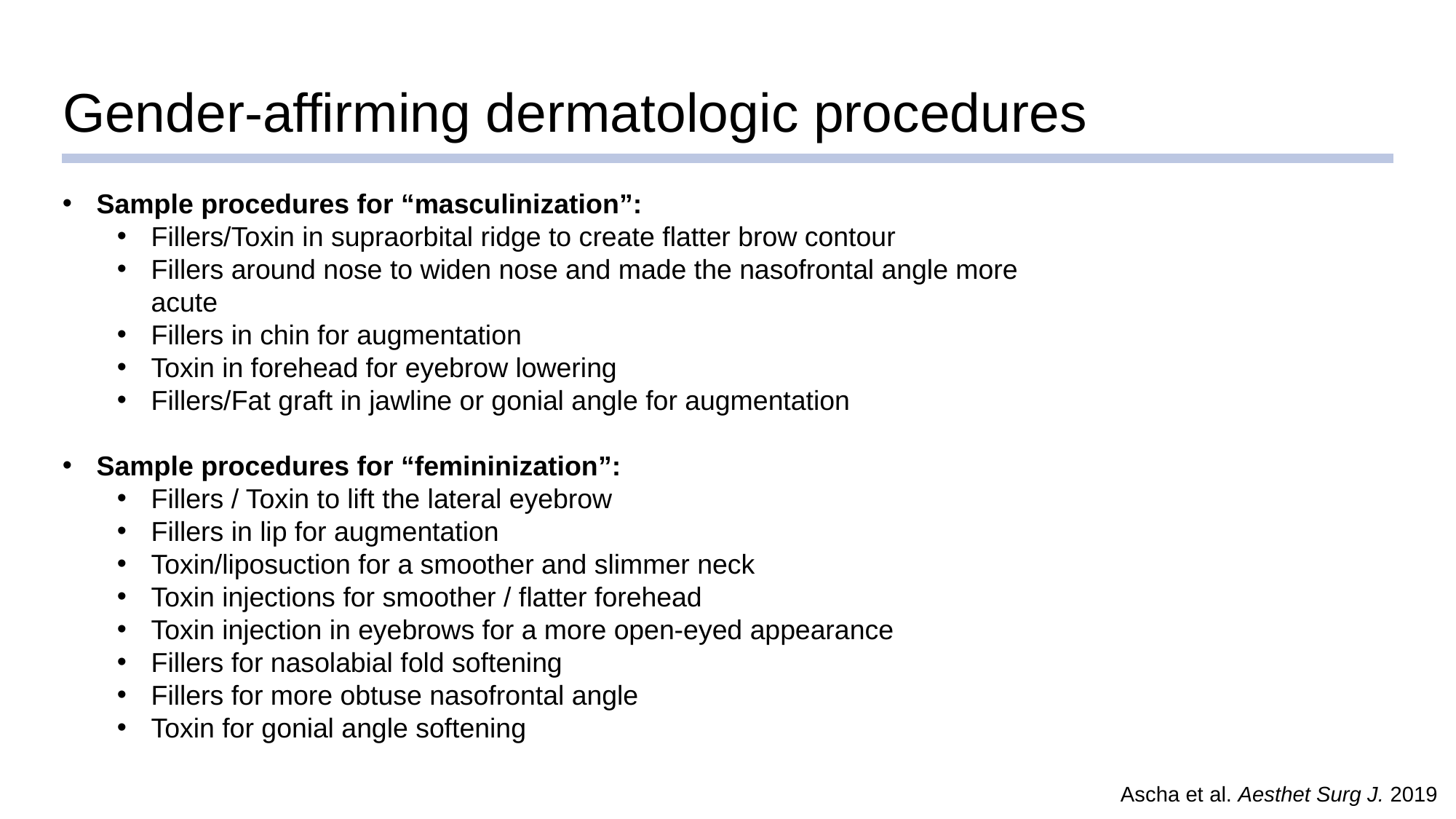

Gender-affirming dermatologic procedures
Sample procedures for “masculinization”:
Fillers/Toxin in supraorbital ridge to create flatter brow contour
Fillers around nose to widen nose and made the nasofrontal angle more acute
Fillers in chin for augmentation
Toxin in forehead for eyebrow lowering
Fillers/Fat graft in jawline or gonial angle for augmentation
Sample procedures for “femininization”:
Fillers / Toxin to lift the lateral eyebrow
Fillers in lip for augmentation
Toxin/liposuction for a smoother and slimmer neck
Toxin injections for smoother / flatter forehead
Toxin injection in eyebrows for a more open-eyed appearance
Fillers for nasolabial fold softening
Fillers for more obtuse nasofrontal angle
Toxin for gonial angle softening
Ascha et al. Aesthet Surg J. 2019

## Slide 63
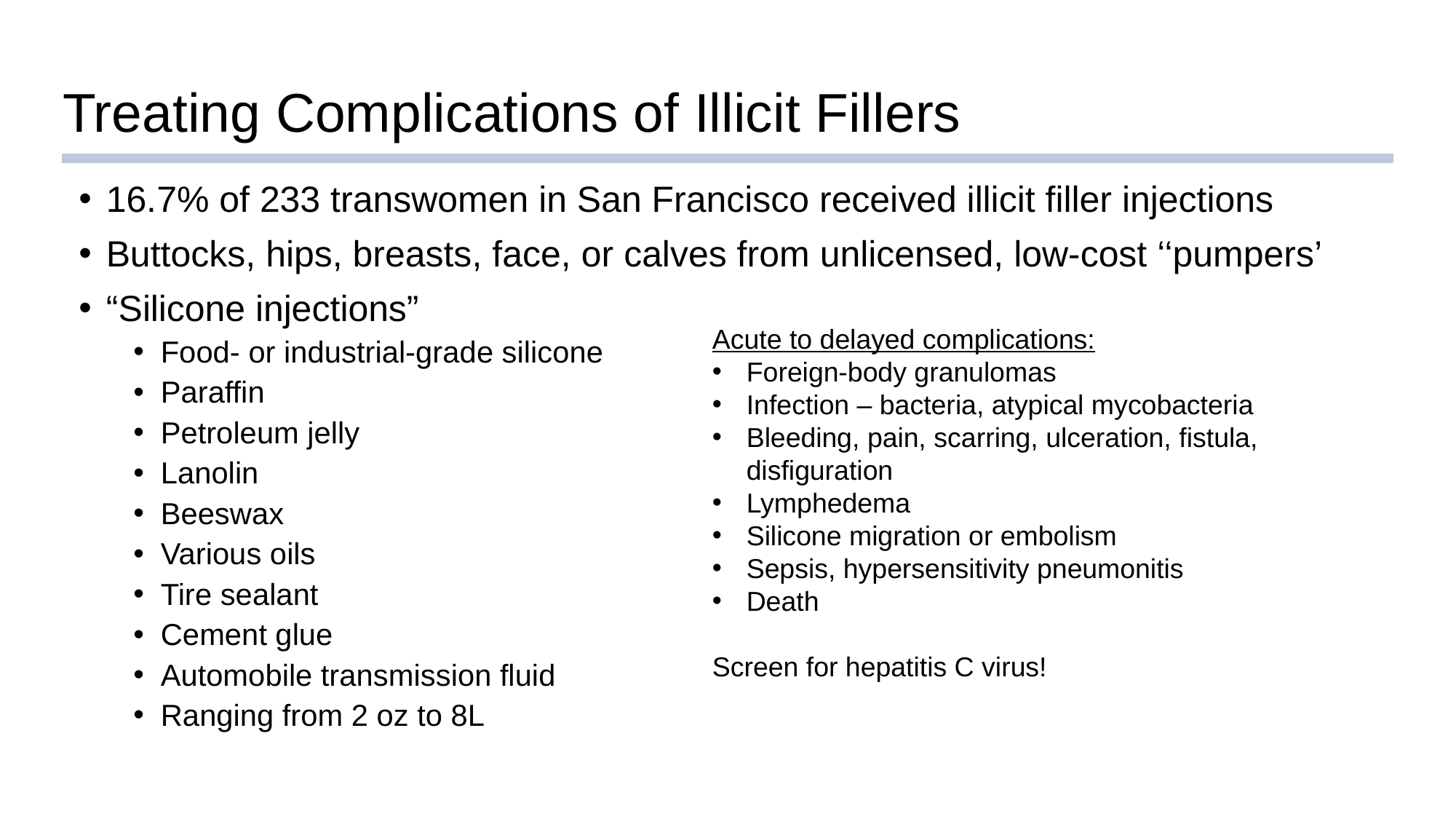

Treating Complications of Illicit Fillers
16.7% of 233 transwomen in San Francisco received illicit filler injections
Buttocks, hips, breasts, face, or calves from unlicensed, low-cost ‘‘pumpers’
“Silicone injections”
Food- or industrial-grade silicone
Paraffin
Petroleum jelly
Lanolin
Beeswax
Various oils
Tire sealant
Cement glue
Automobile transmission fluid
Ranging from 2 oz to 8L
Acute to delayed complications:
Foreign-body granulomas
Infection – bacteria, atypical mycobacteria
Bleeding, pain, scarring, ulceration, fistula, disfiguration
Lymphedema
Silicone migration or embolism
Sepsis, hypersensitivity pneumonitis
Death
Screen for hepatitis C virus!

## Slide 64
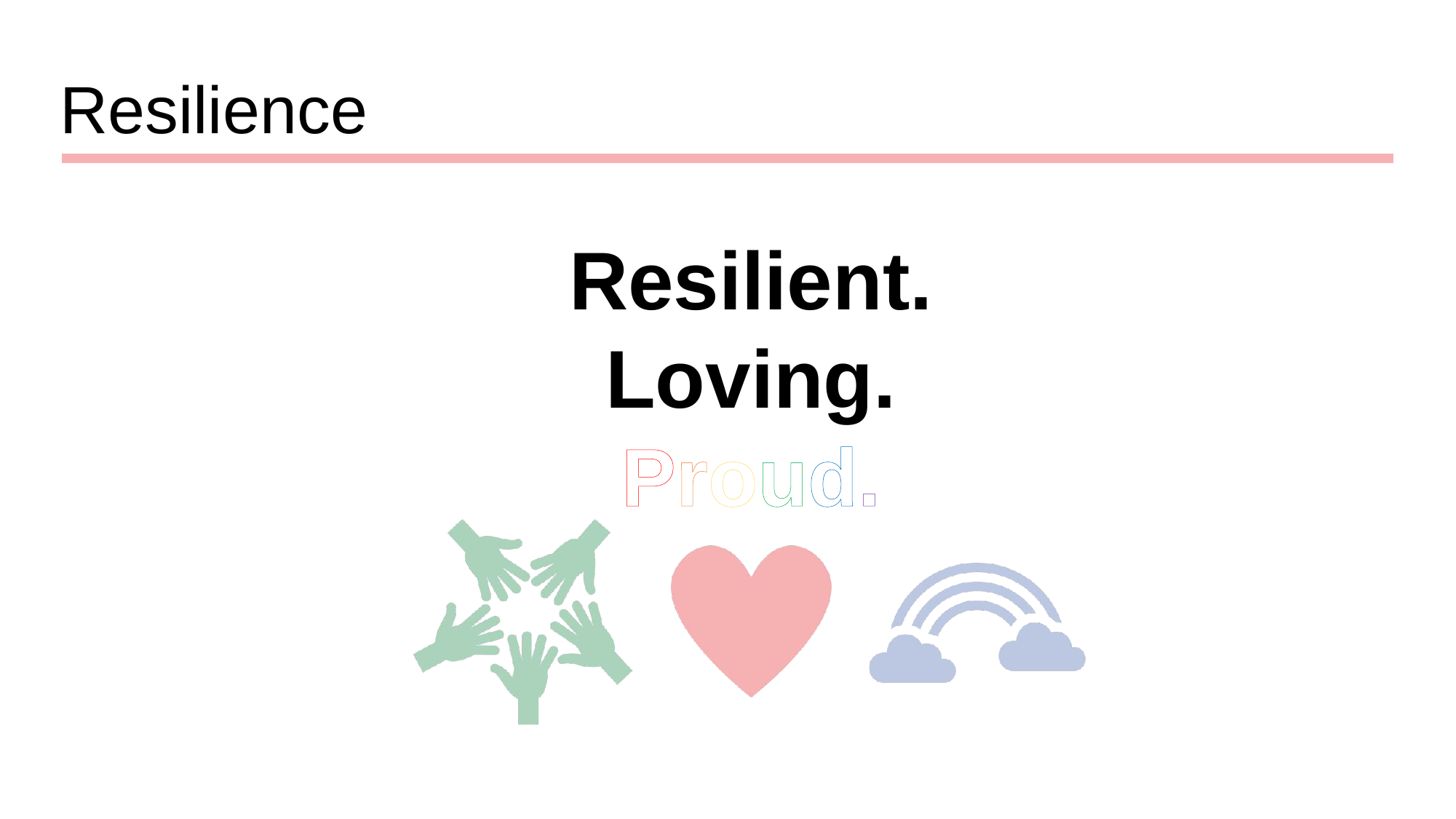

Resilience
Resilient.
Loving.
Proud.

## Slide 65
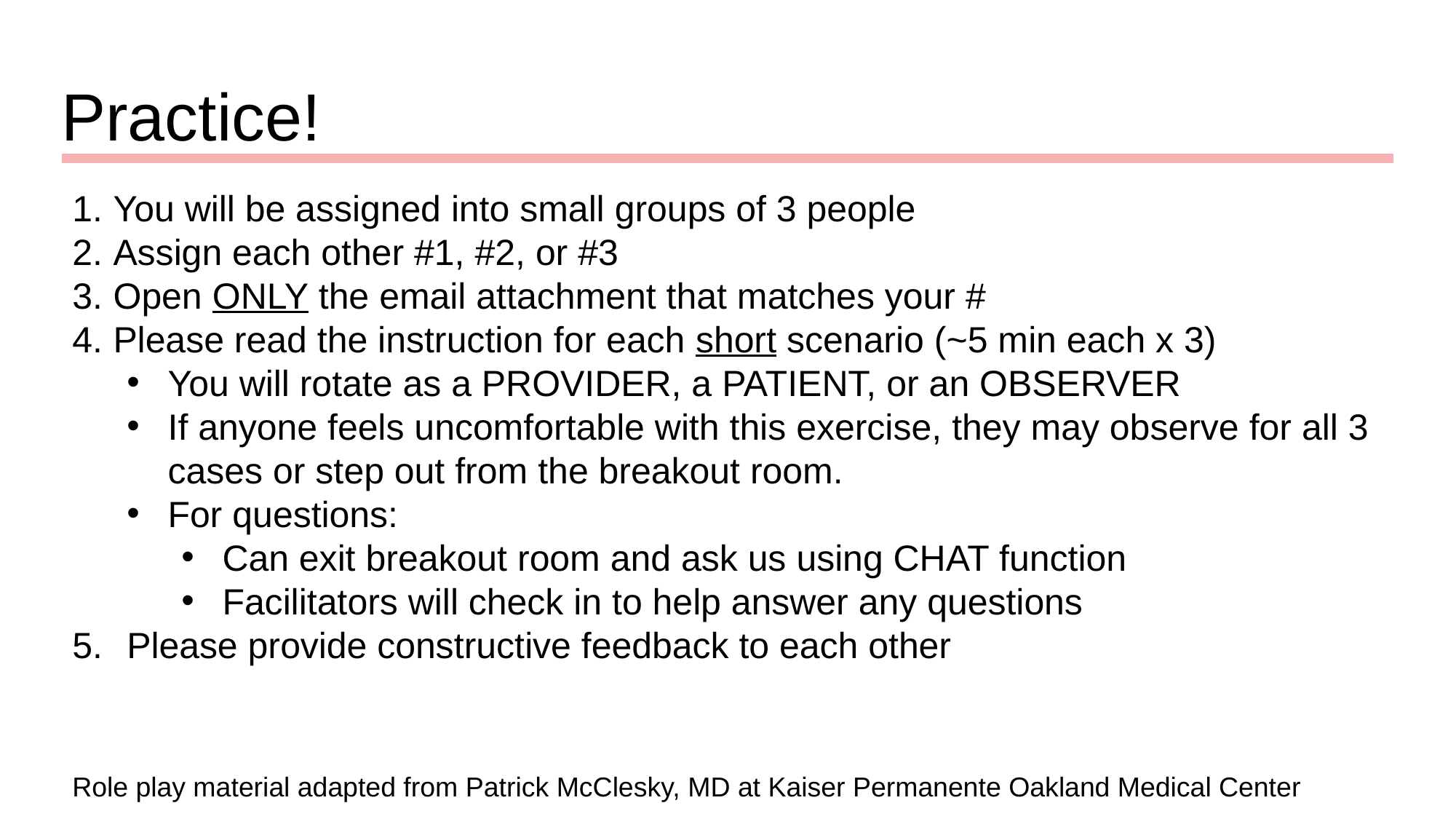

# Practice!
You will be assigned into small groups of 3 people
Assign each other #1, #2, or #3
Open ONLY the email attachment that matches your #
Please read the instruction for each short scenario (~5 min each x 3)
You will rotate as a PROVIDER, a PATIENT, or an OBSERVER
If anyone feels uncomfortable with this exercise, they may observe for all 3 cases or step out from the breakout room.
For questions:
Can exit breakout room and ask us using CHAT function
Facilitators will check in to help answer any questions
Please provide constructive feedback to each other
Role play material adapted from Patrick McClesky, MD at Kaiser Permanente Oakland Medical Center

## Slide 66
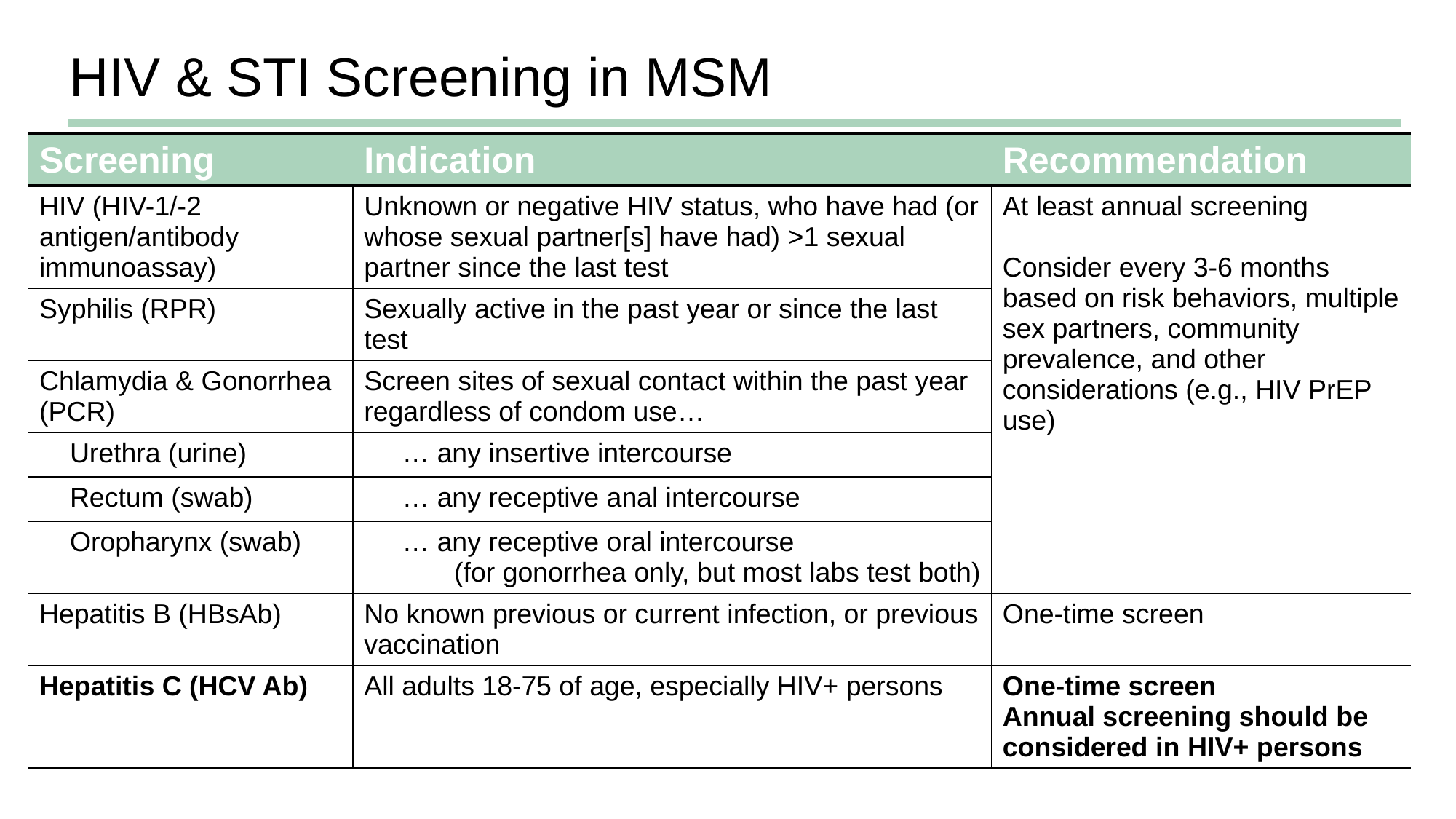

HIV & STI Screening in MSM
| Screening | Indication | Recommendation |
| --- | --- | --- |
| HIV (HIV-1/-2 antigen/antibody immunoassay) | Unknown or negative HIV status, who have had (or whose sexual partner[s] have had) >1 sexual partner since the last test | At least annual screening Consider every 3-6 months based on risk behaviors, multiple sex partners, community prevalence, and other considerations (e.g., HIV PrEP use) |
| Syphilis (RPR) | Sexually active in the past year or since the last test | |
| Chlamydia & Gonorrhea (PCR) | Screen sites of sexual contact within the past year regardless of condom use… | |
| Urethra (urine) | … any insertive intercourse | |
| Rectum (swab) | … any receptive anal intercourse | |
| Oropharynx (swab) | … any receptive oral intercourse (for gonorrhea only, but most labs test both) | |
| Hepatitis B (HBsAb) | No known previous or current infection, or previous vaccination | One-time screen |
| Hepatitis C (HCV Ab) | All adults 18-75 of age, especially HIV+ persons | One-time screen Annual screening should be considered in HIV+ persons |

## Slide 67
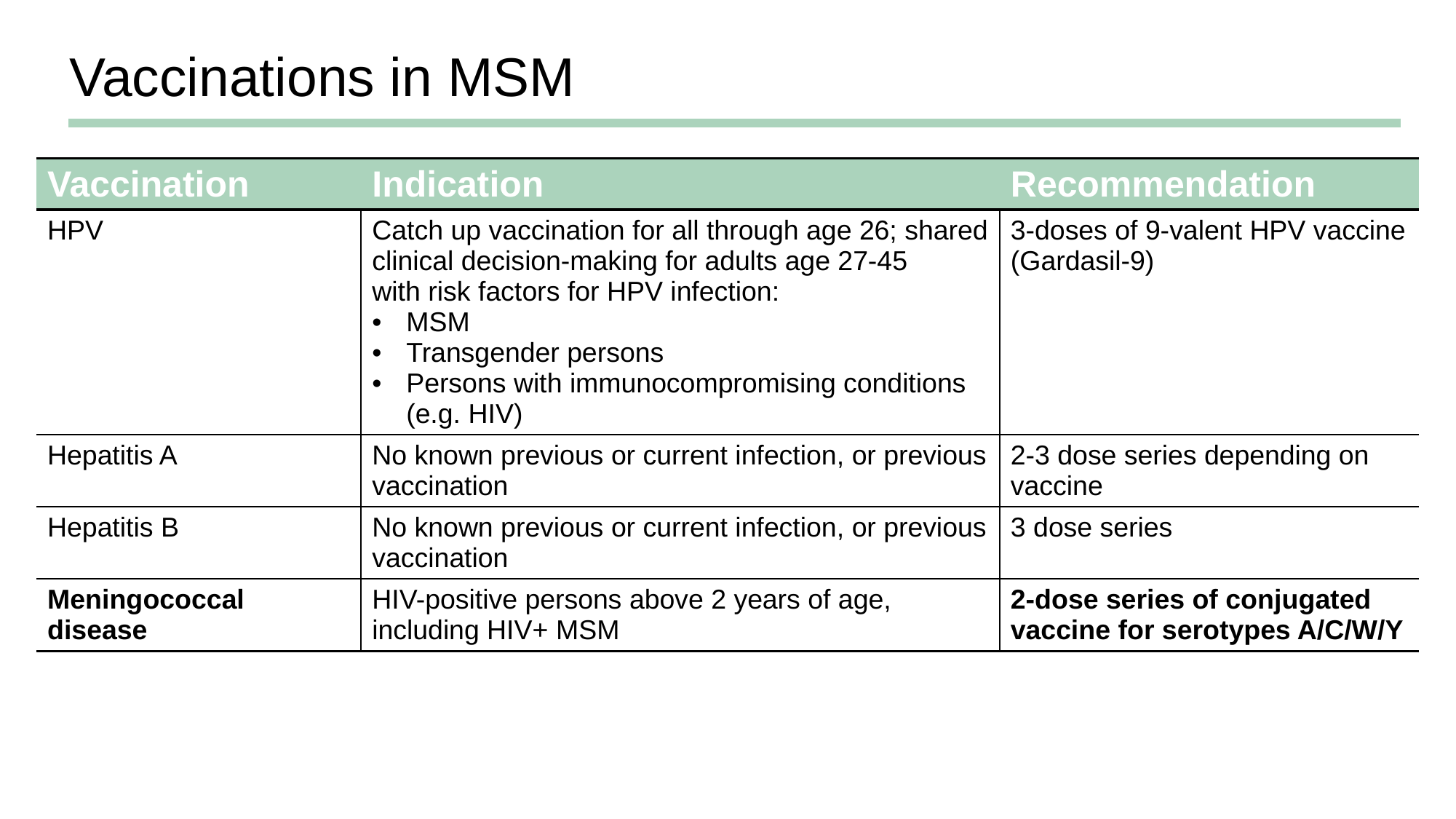

Vaccinations in MSM
| Vaccination | Indication | Recommendation |
| --- | --- | --- |
| HPV | Catch up vaccination for all through age 26; shared clinical decision-making for adults age 27-45 with risk factors for HPV infection: MSM Transgender persons Persons with immunocompromising conditions (e.g. HIV) | 3-doses of 9-valent HPV vaccine (Gardasil-9) |
| Hepatitis A | No known previous or current infection, or previous vaccination | 2-3 dose series depending on vaccine |
| Hepatitis B | No known previous or current infection, or previous vaccination | 3 dose series |
| Meningococcal disease | HIV-positive persons above 2 years of age, including HIV+ MSM | 2-dose series of conjugated vaccine for serotypes A/C/W/Y |

## Slide 68
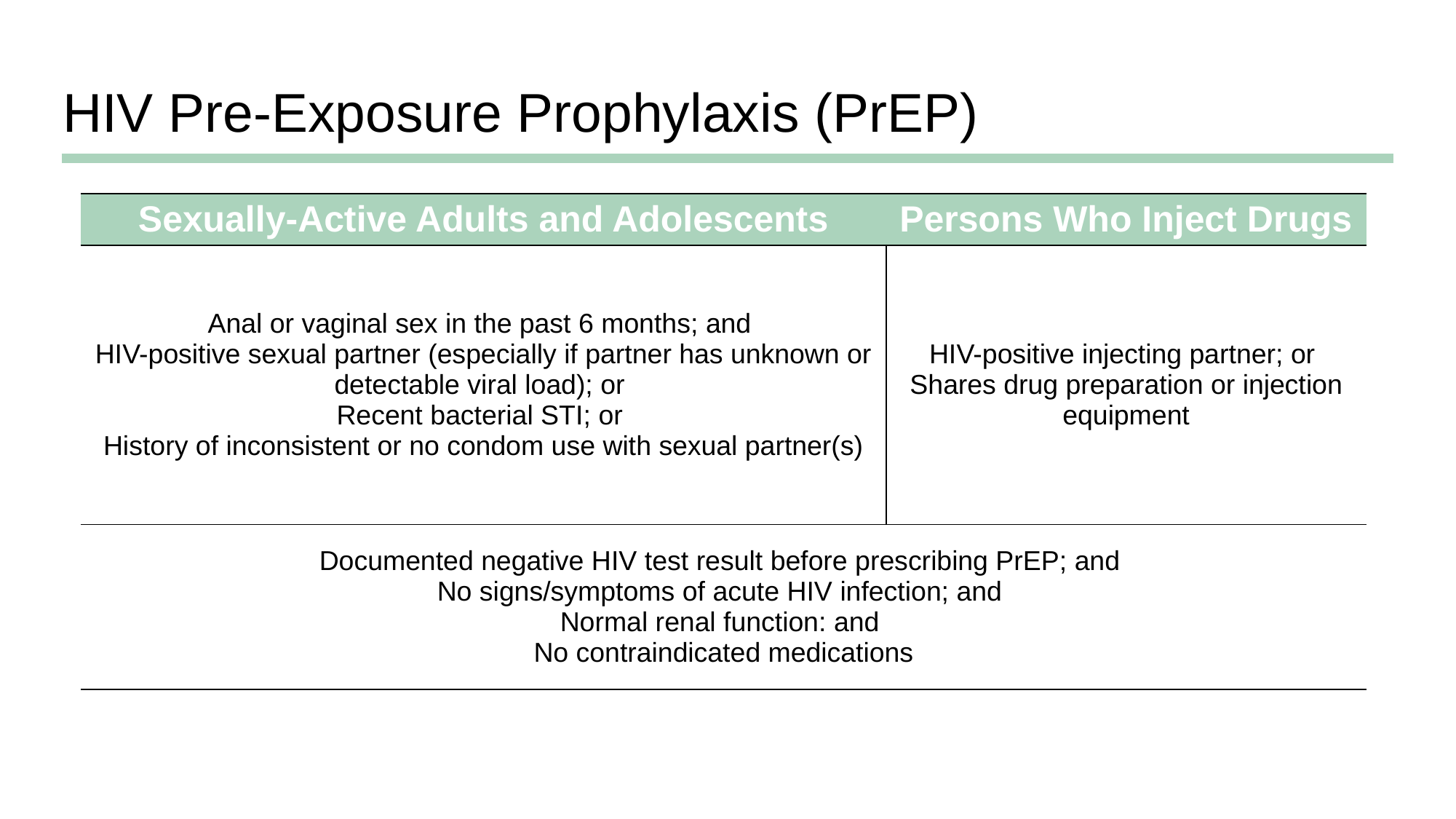

HIV Pre-Exposure Prophylaxis (PrEP)
| Sexually-Active Adults and Adolescents | Persons Who Inject Drugs |
| --- | --- |
| Anal or vaginal sex in the past 6 months; and HIV-positive sexual partner (especially if partner has unknown or detectable viral load); or Recent bacterial STI; or History of inconsistent or no condom use with sexual partner(s) | HIV-positive injecting partner; or Shares drug preparation or injection equipment |
| Documented negative HIV test result before prescribing PrEP; and No signs/symptoms of acute HIV infection; and Normal renal function: and No contraindicated medications | |

## Slide 69
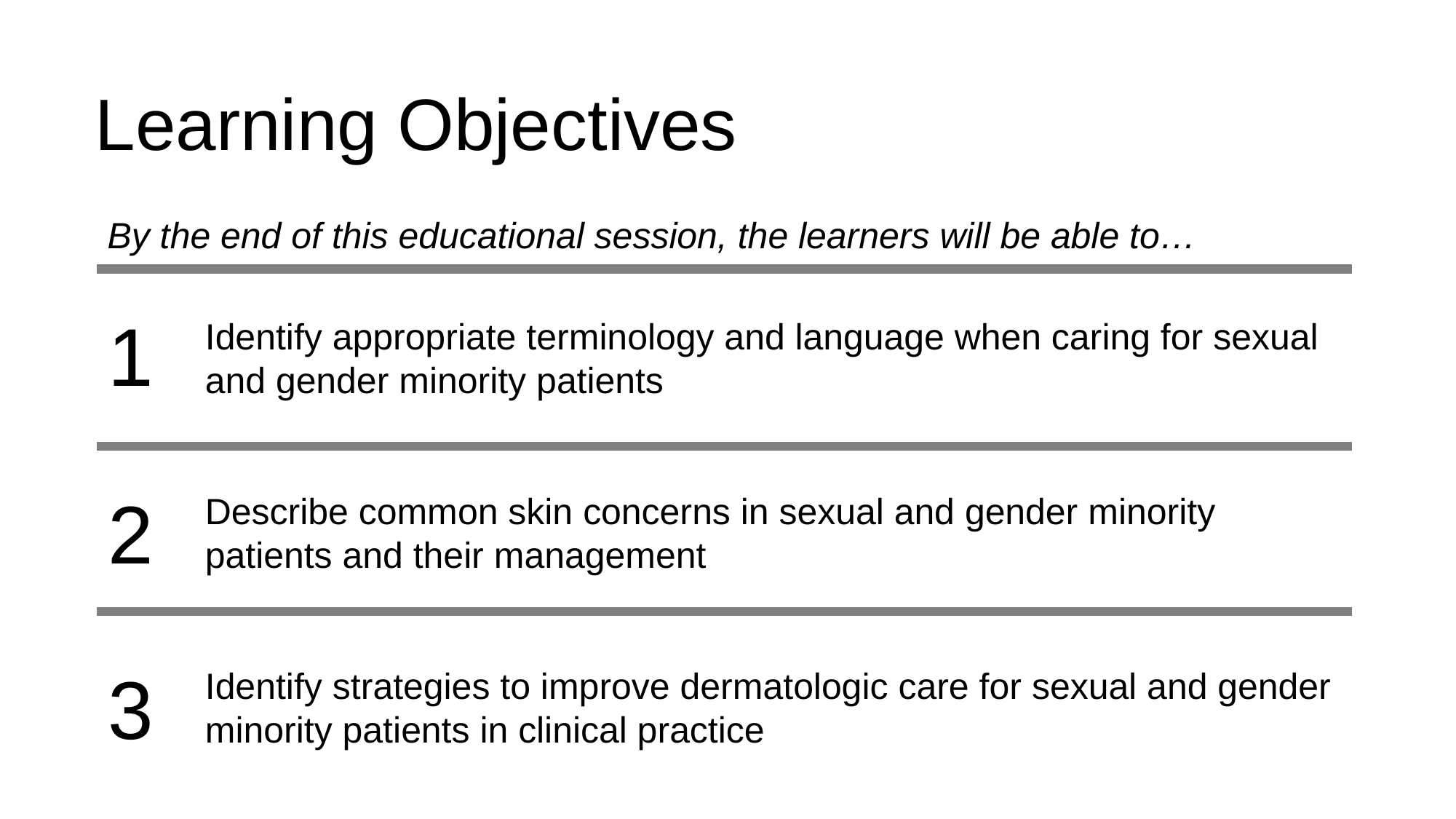

# Learning Objectives
By the end of this educational session, the learners will be able to…
1
Identify appropriate terminology and language when caring for sexual and gender minority patients
Describe common skin concerns in sexual and gender minority patients and their management
Identify strategies to improve dermatologic care for sexual and gender minority patients in clinical practice
2
3

## Slide 70
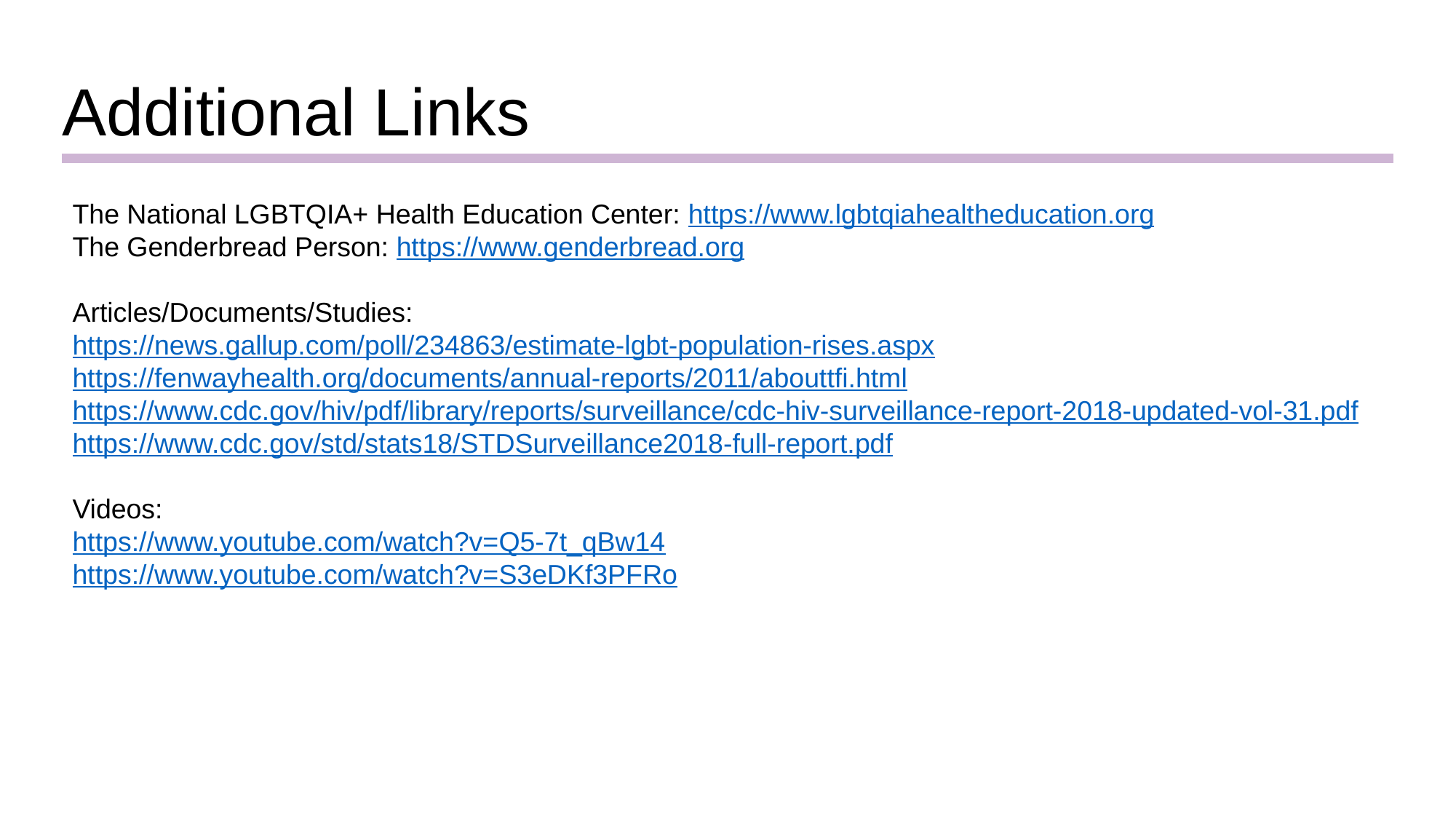

Additional Links
The National LGBTQIA+ Health Education Center: https://www.lgbtqiahealtheducation.org
The Genderbread Person: https://www.genderbread.org
Articles/Documents/Studies:
https://news.gallup.com/poll/234863/estimate-lgbt-population-rises.aspx
https://fenwayhealth.org/documents/annual-reports/2011/abouttfi.html
https://www.cdc.gov/hiv/pdf/library/reports/surveillance/cdc-hiv-surveillance-report-2018-updated-vol-31.pdf
https://www.cdc.gov/std/stats18/STDSurveillance2018-full-report.pdf
Videos:
https://www.youtube.com/watch?v=Q5-7t_qBw14
https://www.youtube.com/watch?v=S3eDKf3PFRo

## Slide 71
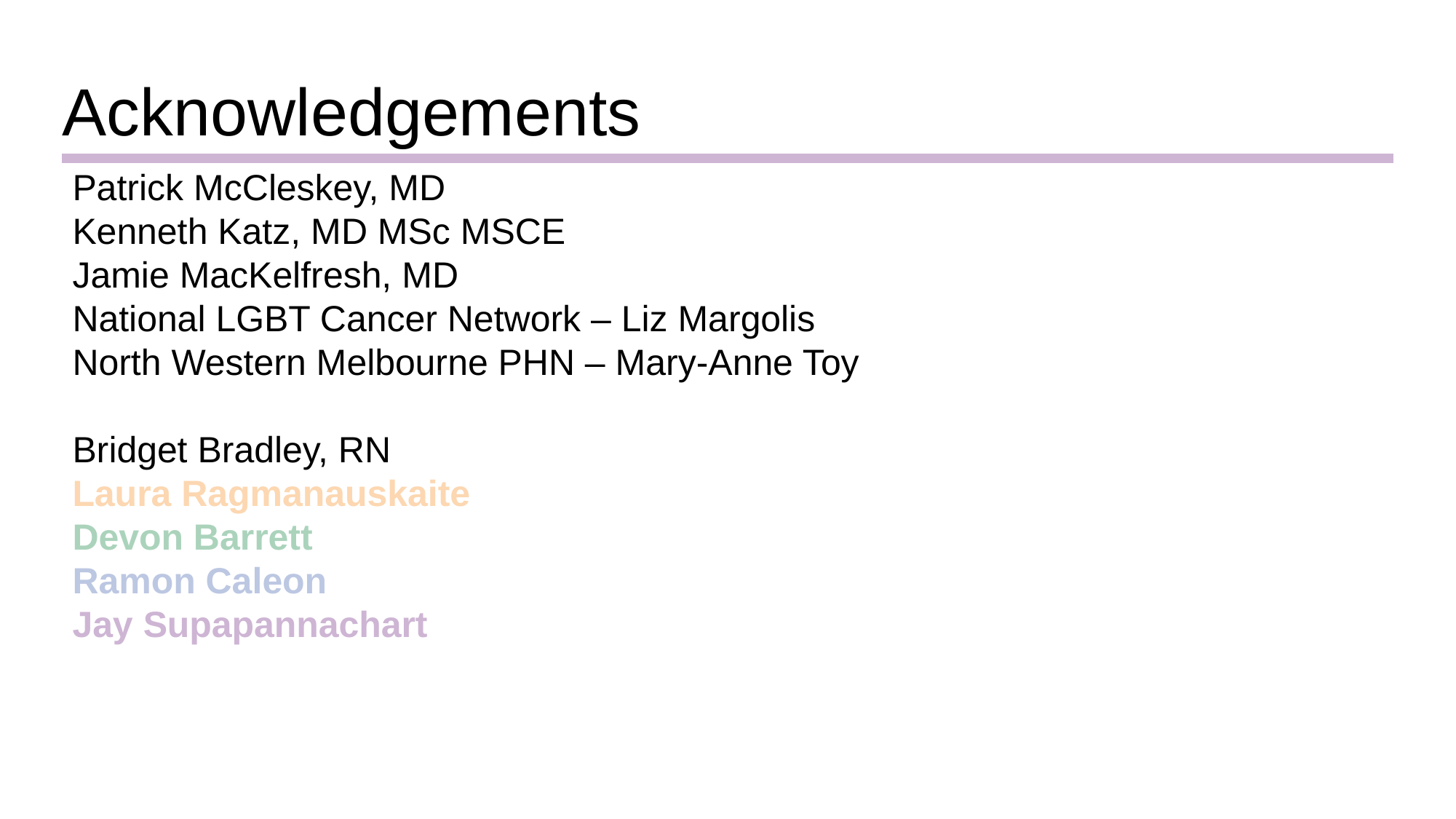

Acknowledgements
Patrick McCleskey, MD
Kenneth Katz, MD MSc MSCE
Jamie MacKelfresh, MD
National LGBT Cancer Network – Liz Margolis
North Western Melbourne PHN – Mary-Anne Toy
Bridget Bradley, RN
Laura Ragmanauskaite
Devon Barrett
Ramon Caleon
Jay Supapannachart
