# Supplementary material for: Interactive Session for Residents and Medical Students on Dermatologic Care for Lesbian, Gay, Bisexual, Transgender, and Queer Patients
Source: MedEdPORTAL. 2021 Apr 21;17:11148. doi: 10.15766/mep_2374-8265.11148 (PMC8063631; doi:10.15766/mep_2374-8265.11148)
Supplement: Supplementary file 1 — LGBTQ Curriculum Presentation.pptxCase 1.docxCase 2.docxCase 3.docxBaseline Survey.docxFollow-up Survey.docx [file mep_2374-8265.11148-s001.zip › C. Case 2.docx]

**Exercise: Practice taking a sexual history**

You have been assigned **ROLE 2**. This means you will play the following roles for each scenario:

| **Scenario** | **Role** |
| --- | --- |
| 1 | PROVIDER |
| 2 | PATIENT |
| 3 | OBSERVER* |

You will automatically be put in a group of three with two other people using the Zoom breakout session feature. ***Alternatively, if any person feels uncomfortable with this exercise they may play the observer for all three cases.**

**TURN TO THE NEXT PAGE FOR SCENARIO 1**

**SCENARIO 1: PROVIDER**

FOR THE SCENARIO...

The PROVIDER will conduct a brief sexual history and will also aim to identify any guideline recommended screening / diagnostic tests based on the PATIENT’S risk factors.

1. **Introduction (1 minute)**
   1. Introduce yourself to the patient (feel free to include your pronouns)
   2. Ask how they would like to be addressed and what pronouns they use
   3. Ask an open-ended question about their **chief complaint**
2. **Sexual History (2 minutes)**
   1. Normalize and discuss confidentiality
   2. 5 P’s of Sexual History (examples below, not all may apply to your case)
      1. … Do you have sex? with men, women, or both?
         … What are the bodies and genders do you have sex with?
         … How many people have you had sex within the past year (since your last STI test)?
         … Do you have a primary partner?
      2. … How do you have sex with them? … Which body parts do you use?
         … What kind of sex have you had? Oral sex? Vaginal sex? Anal sex (insertive ‘‘top,’’ receptive ‘‘bottom,’’ or both ‘‘versatile’’)?
      3. … When were your last HIV and STI tests? … What were the results?
         … Have you ever been diagnosed with HIV or any other STDs?
      4. … How often do you use barriers or condoms to prevent pregnancy or STDs?
         … Have you heard or thought about PrEP (if appropriate)?
      5. … Do you desire pregnancy now or in the future?
3. After completing of sexual history taking, decide which of the following tests may be appropriate to recommend to your patient. Inform the patient about your recommendations: (**1 minute)**
   1. HIV-1/HIV-2 antibody/antigen screen
   2. Rapid plasma reagin (for syphilis)
   3. Urethral Chlamydia / gonorrhea
   4. Pharyngeal Chlamydia / gonorrhea swab
   5. Rectal Chlamydia / gonorrhea swab
   6. Cervical Chlamydia / gonorrhea swab
   7. Hepatitis C serum antibody

AFTER THE SCENARIO IS COMPLETED...

Please discuss scenario with the PATIENT and then the OBSERVER.

**TURN TO THE NEXT PAGE FOR SCENARIO 2**

**SCENARIO 2: PATIENT**

FOR THE SCENARIO...

1. Please read the introduction and patient scenario.
2. Let the PROVIDER ask you questions and only answer questions that the PROVIDER asks.

INTRODUCTION: I’m a 36-year-old gay male, and I’m returning to **make sure a single wart on my anus went away with cryotherapy your colleague did a month ago**

Partners: Approximately 60 male partners since last STI check 4 months ago;

Hx: open relationship with boyfriend; both are HIV negative and on PreP.

Behaviors: has receptive and insertive oral sex; only has insertive anal sex. Uses condoms about half the time, not at all with boyfriend. Has not received penetrative anal sex or rimming since he had this wart >4 months ago. He uses GHB and poppers during recreational sex; smoked methamphetamine 2 years ago but in recovery now. No children and no plan for children.

AFTER THE SCENARIO IS COMPLETED...

1. Give constructive feedback on:
   - Use of inclusive and patient-preferred language
   - Normalizing history taking and discussion of confidentiality
   - Maintaining professionalism and respect
   - Any relevant / missed information from the scenario
2. The OBSERVER will provide any additional feedback.

[FYI] the PROVIDER should offer the following tests:

- **HIV serology** – HIV negative MSM with >1 partner since the last check
- **RPR –** MSM with multiple partners and intermittent condom use should be screened every 3-6 months
- **Urethral and Pharyngeal Chlamydia / gonorrhea** – insertive anal and oral sex (urethral), receptive oral sex (pharyngeal). Rectal screening is not required if patient had prior negative testing and does not engage in receptive anal sex.

**TURN TO THE NEXT PAGE FOR SCENARIO 3**

**SCENARIO 3: OBSERVER**

FOR THE SCENARIO...

Please observe whether the provider addressed the following:

- Elicit and use inclusive and patient preferred language
- Elicit open-ended history about CC / HPI
- Normalize asking sensitive questions
- Establish confidentiality
- Elicit 5 P’s: __ Partners, ___ Practices, __ Past history of STI testing & diagnoses,
   ___ Protection from STI, ___ Pregnancy Plans
- Recognize non-verbal clues
- Maintain professionalism, open-mindedness, non-judgmental tone, respect
- Offer appropriate testing based on risk factors

[Scenario]

**Patient Information:**

Patient 3: 28 y/o transgender male

Patient Intro: I’m a 28-year-old transgender male you’re seeing for the first time for suspected **pityriasis rosea on the trunk**

Partners: 3 female, 3 male, in the past year

Hx: Last STI check: 18 months ago (HIV, cervical chl/GC negative)

Surgical Hx: chest reconstruction 4 years ago; no other gender affirmation surgery; Organs present: ovaries, uterus; taking testosterone undecanoate 100mg subcutaneous injection every 12 weeks for past 5 years.

Behaviors: always uses condoms for receptive vaginal sex with male partners. Does not use other forms of contraception; receptive oral sex with men; no receptive anal sex in past two years. No immediate plan for children but may change his mind in the future.

[FYI] the PROVIDER should offer the following tests:

- **HIV serology** – HIV negative with >1 partner since the last STI check
- **RPR**– diagnostic testing to rule out secondary syphilis Pityriasis rosea
- **[Urethral, Vaginal, OR Cervical] and Oropharyngeal Chlamydia / Gonorrhea** – Receptive vaginal sex (urethral or vaginal, or endocervical) and receptive oral sex (oropharyngeal).

AFTER THE SCENARIO IS COMPLETED...

After the PATIENT provided their feedback, please provide any additional constructive feedback based on the above checklist, including what went well and what opportunities there are for improvement.
