# Supplementary material for: Interactive Session for Residents and Medical Students on Dermatologic Care for Lesbian, Gay, Bisexual, Transgender, and Queer Patients
Source: MedEdPORTAL. 2021 Apr 21;17:11148. doi: 10.15766/mep_2374-8265.11148 (PMC8063631; doi:10.15766/mep_2374-8265.11148)
Supplement: Supplementary file 1 — LGBTQ Curriculum Presentation.pptxCase 1.docxCase 2.docxCase 3.docxBaseline Survey.docxFollow-up Survey.docx [file mep_2374-8265.11148-s001.zip › F. Follow Up Survey.docx]

**Consent**

1. Do you agree to take part in this study?

Yes

No

**Identifier**

2. What is your favorite animal?

3. What is your favorite skin lesion?

**Attitudes**

The first series of questions will ask about your personal attitudes, comfort level, and current practices when caring for members of LGBT community.

Indicate how strongly you agree or disagree with the following statements:

4^1^. I am aware of institutional barriers that may inhibit transgender people from using health care services

*Mark only one oval.*

1 2 3 4 5 6 7

Where 1 = strongly disagree and 7 = strongly agree

5^1^. I am aware of institutional barriers that may inhibit lesbian, gay, and bisexual (LGB) people from using health services

*Mark only one oval.*

1 2 3 4 5 6 7

Where 1 = strongly disagree and 7 = strongly agree

6^1^. I think being transgender is a mental disorder

*Mark only one oval.*

1 2 3 4 5 6 7

Where 1 = strongly disagree and 7 = strongly agree

7^1^. I would feel unprepared talking with a lesbian, gay, bisexual, and transgender client/patient about issues related to their sexual orientation or gender identity.

*Mark only one oval.*

1 2 3 4 5 6 7

Where 1 = strongly disagree and 7 = strongly agree

8^1^. A same sex relationship between two men or two women is NOT as strong and committed as one between a man and a woman.

*Mark only one oval.*

1 2 3 4 5 6 7

Where 1 = strongly disagree and 7 = strongly agree

9^1^. I am aware of research indicating that LGB individuals experience disproportionate level of health and mental health problems compared to heterosexual individuals.

*Mark only one oval.*

1 2 3 4 5 6 7

Where 1 = strongly disagree and 7 = strongly agree

10^1^. LGB individuals must be discreet about their sexual orientation around children.

*Mark only one oval.*

1 2 3 4 5 6 7

Where 1 = strongly disagree and 7 = strongly agree

11^1^. I am aware of research indicating that transgender individuals experience disproportionate levels of health and mental health problems compared to cisgender individuals

*Mark only one oval.*

1 2 3 4 5 6 7

Where 1 = strongly disagree and 7 = strongly agree

12^1^. When it comes to transgender individuals, I believe they are morally deviant.

*Mark only one oval.*

1 2 3 4 5 6 7

Where 1 = strongly disagree and 7 = strongly agree

13^1^. I have received adequate clinical training and supervision to work with transgender clients/patients.

*Mark only one oval.*

1 2 3 4 5 6 7

Where 1 = strongly disagree and 7 = strongly agree

14^1^. I have received adequate clinical training and supervision to work with LGBT client/patients.

*Mark only one oval.*

1 2 3 4 5 6 7

Where 1 = strongly disagree and 7 = strongly agree

15^1^. The lifestyle of a LGB individual is unnatural or immoral

*Mark only one oval.*

1 2 3 4 5 6 7

Where 1 = strongly disagree and 7 = strongly agree

16^1^. I have experience working with LGB clients/patients

*Mark only one oval.*

1 2 3 4 5 6 7

Where 1 = strongly disagree and 7 = strongly agree

17^1^. I feel competent to asses a person who is LGB in a therapeutic setting

*Mark only one oval.*

1 2 3 4 5 6 7

Where 1 = strongly disagree and 7 = strongly agree

18^1^. I feel competent to asses a person who is transgender in a therapeutic setting

*Mark only one oval.*

1 2 3 4 5 6 7

Where 1 = strongly disagree and 7 = strongly agree

19^1^. I have experience working with transgender clients/patients.

*Mark only one oval.*

1 2 3 4 5 6 7

Where 1 = strongly disagree and 7 = strongly agree

20^1^. People who dress OPPOSITE to their biologic sex have a perversion.

*Mark only one oval.*

1 2 3 4 5 6 7

Where 1 = strongly disagree and 7 = strongly agree

21^1^. I would be morally uncomfortable working with a lesbian, gay, bisexual and transgender client/patient.

*Mark only one oval.*

1 2 3 4 5 6 7

Where 1 = strongly disagree and 7 = strongly agree

22^2^***. I am comfortable working alongside LGBTQ physicians

*Mark only one oval.*

1 2 3 4 5 6 7

Where 1 = strongly disagree and 7 = strongly agree

23. Gender identity is a person's method to communicate their gender through appearance, personality, or behaviors.

*Mark only one oval.*

True

False

24. Sexual orientation is how a person characterizes their emotional and sexual attraction to others.

*Mark only one oval.*

True

False

25. Obtaining a comprehensive sexual history includes asking about (CHECK ALL THAT APPLY)

*Check all that apply.*

Do you have sex with women, men, or both?

Are you using birth control?

When was the last time you were tested for STI's?

How are you protecting yourself from STI's including HIV?

What type of sex are you having?

26. Ash is a 28 year old patient in your clinic and complains of a genital rash. Select the statement that DOES NOT have inclusive language

*Mark only one oval.*

Do you have sex with women, men, or both?

Has your girl friend had a similar rash?

When was your last sexual encounter?

How often do you use condoms?

When was the last time you were tested for STIs?

27. You diagnosed Ash with intertrigo. Ash asks if there are any tests that you may recommend for concerns about sexually transmitted diseases. Ash identifies as a gay man. Ash had a negative HIV test 1 year ago and does not recall any prior vaccinations. Which of the following test(s) would you recommend for Ash (CHECK ALL THAT APPLY)?

HIV1/2 antibody screening

HIV-1 RNA PCR

RPR

FTA-ABS

Urine Gonorrhea/ Chlamydia

Pharyngeal Gonorrhea/ Chlamydia Swab

Rectal Gonorrhea/ Chlamydia swab

Anal Pap smear

HPV

Hepatitis B surface antibody

Hepatitis B core antibody

Hepatitis C surface antibody

HSV 1/2 serology

28. Jo is a transgender man who was assigned female at birth. He receives testosterone injections and has developed severe acne not responding to oral antibiotics. Based on his anatomy, you determined that he has reproductive potential. In prescribing isotretinoin to Jo, according to iPLEDGE, he must comply with 2 forms of reliable contraception. Which of the following may be appropriate PRIMARY forms of contraception (CHECK ALL THAT APPLY)?

*Check all that apply.*

Birth Control Pills

Intrauterine Device

Abstinence

Diaphragm

Tubal Ligation

Vaginal Ring

Condoms

Vasectomy

Hormonal Patch

Hormonal Injection

Hormonal Implant

Cervical Cap

Vaginal Sponge

29. Persons who have received illicit fillers or injections should be screened for Hepatitis C

*Mark only one oval.*

True

False

**Overall Session Evaluation**

This section of the survey will assess the overall effectiveness of the lecture.

Please rate the quality of this lecture in regards to:

30 How valuable was the overall content to your practice?

Poor; Fair; Average; Very Good; Excellent

31 How effective were the educational materials used during this lecture?

Poor; Fair; Average; Very Good; Excellent

32 Usefulness: Please identify aspects or tools from today’s session that you may use in your future practice when caring for LGBTQ patients?

*Free response; please use at least 20 words*

33 Suggestions for Improvement: please identify AT LEAST ONE aspect of the lecture that you think could be improved, and describe how it could be improved.

*Free response; please respond with at least 20 words.*

**Questions adapted from Moll et al 2019*

1. Bidell MP. The Lesbian, Gay, Bisexual, and Transgender Development of Clinical Skills Scale (LGBT-DOCSS): Establishing a New Interdisciplinary Self-Assessment for Health Providers. *J Homosex.* 2017;64(10):1432-1460.

2. Moll J, Krieger P, Heron SL, Joyce C, Moreno-Walton L. Attitudes, Behavior, and Comfort of Emergency Medicine Residents in Caring for LGBT Patients: What Do We Know? *AEM Educ Train.* 2019;3(2):129-135.
